# Immunogenicity of Del19 EGFR mutations in Chinese patients affected by lung adenocarcinoma

**DOI:** 10.1186/s12865-019-0320-1

**Published:** 2019-11-13

**Authors:** Deng Pan, Dapeng Zhou, Weijing Cai, Weibo Wu, Wen Ling Tan, Caicun Zhou, Yanyan Lou

**Affiliations:** 1 Shanghai Pudong New Area Mental Health Center affiliated with Tongji University School of Medicine, Shanghai, 200092 China; 2 Shanghai Pulmonary Hospital affiliated with Tongji University School of Medicine, Shanghai, 200092 China; 3 Mayo Clinic, Jacksonville, FL 32224 USA

**Keywords:** Lung cancer, Neo-antigen, INDEL, Immunotherapy, PD1 checkpoint blocking antibody

## Abstract

**Background:**

Mutant peptides presented by cancer cells are superior vaccine candidates than self peptides. The efficacy of mutant K-Ras, P53 and EGFR (Epidermal Growth Factor Receptor) peptides have been tested as cancer vaccines in pancreatic, colorectal, and lung cancers. The immunogenicity of EGFR Del19 mutations, frequent in Chinese lung adenocarcinoma patients, remains unclear.

**Results:**

We predicted the HLA binding epitopes of Del19 mutations of EGFR in Chinese lung adenocarcinoma patients with NetMHC software. Enzyme-linked immunosorbent assay (ELISA) was performed to detect the EGFR-reactive IgG in lung cancer patients. Del19 mutations may be presented by multiple HLA Class I molecules, with delE746_A750 presented by 37.5% of Chinese population. For HLA Class II molecules, Del19 mutations of EGFR may be presented by multiple HLA-DRB1 molecules, with delE746_A750 presented by 58.1% of Chinese population. Serum reactivity to wild type EGFR protein was significantly higher in patients with Del19 EGFR mutations than those with EGFR L858R point mutation or with EGFR wild type genotype.

**Conclusions:**

These findings suggest that Del19 mutations of EGFR, with an estimated frequency of 40% in Chinese lung adenocarcinoma patients, may serve as unique targets for immunotherapy in Chinese lung cancer patients.

## Background

Vaccine therapy for cancer has gained very limited success, with the example of Provenge (Sipuleucel-T), a dendritic cell based vaccine loaded with a fusion protein, PA2024, composed of recombinant PAP fused to granulocyte–macrophage colony-stimulating factor (GM-CSF), a cytokine that stimulates antigen presenting cells [1, 2]. Sipuleucel-T has extended survival of metastatic prostate cancer patients by median 4.1 months (IMPACT Phase III trial data). Other cancer vaccines based on self-proteins have shown very limited success in improving overall survival, which is not surprising in view of the expression of such self-proteins in healthy tissues and organs. For example, MUC1, a widely studied vaccine candidate, is expressed at similar abundance in healthy tissues as lung cancer according to mRNA array data of 442 lung adenocarcinoma patients deposited in the Cancer Genome Atlas [3, 4].

Mutant vaccines for cancer therapy was proposed decades ago, and have been focus of current immunotherapeutic studies after a series of findings that neoantigens in chronic lymphocytic leukemia, melanoma, and multiple cancer types are functional [5–12]. Neoantigens identified by exon sequencing and predicted by bioinformatics studies could stimulate autologous T cells [13, 14]. Furthermore, dendritic cell (DC) vaccines loaded with predicted neoantigen peptides increased the breadth and diversity of melanoma neoantigen-specific T cells, proving that such neoantigens are functional to stimulate CD8 T cells in vivo [15]. More excitingly, recent clinical data showed that colorectal cancer with a large number of somatic mutations due to mismatch-repair defects are more susceptible to immune checkpoint blockade by anti-PD-1 antibody therapy [16]. Resistance to immune checkpoint blockade therapy by anti-PD1 or anti-PD1/anti-CTLA-4 therapy is associated with loss of neoantigens [17].

Both point mutations and insertion or deletion (INDEL) type of mutations have been found in lung cancer patients. According to the Catalog Of Somatic Mutations In Cancer (COSMIC), 594 types of EGFR mutations have been reported, with 93% of mutations are present in the gene encoding tyrosine kinase domain (exons 18 to 21). East Asian lung cancer patients (40%) showed much higher EGFR mutation frequency than Caucasian patients (20%). Exon 19 deletions constitute 44.8% of EGFR mutations in East Asian patients [18].

In this study, we studied whether the Del19 mutations of EGFR may serve as targets for immunotherapy. We studied the MHC binding capacity of neoantigen peptides by NetMHC [19]. We also measured the serum antibody responses to EGFR protein (wild type) in lung cancer patients with different EGFR mutations.

## Results

High-affinity candidate T cell epitopes were identified in silico by scanning of the mutant peptides (Table 1). We focused on identified HLA class I gene alleles with high expression levels in humans [20]. 66 neo-peptides were identified for multiple HLA Class I molecules (Additional file 1: Table S1, Additional file 2: Table S2, Additional file 3: Table S3, Additional file 4: Table S4, Additional file 5: Table S5, Additional file 6: Table S6, Additional file 7: Table S7, Additional file 8: Table S8, Additional file 9: Table S9, Additional file 10: Table S10 and Additional file 11: Table S11). Del19 mutations may be presented by HLA Class I molecules with a frequency ranging from 11.2 to 82.1%, with delE746_A750 presented by 37.5% of Chinese population.

**Table 1:**
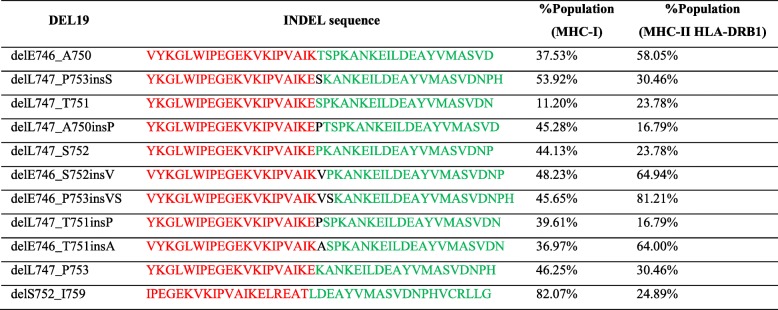
Frequency of EGFR Del19 presentation by Chinese NSCLC patients as predicted by NetMHC4.0

The percentages are the total frequencies of HLA alleles which may present a mutant EGFR. Threshold of binding affinity for potential neoantigens was defined as IC50 < 500 nM. Both MHC I and MHC II were listed. HLA alleles (HLA-A, HLA-B, HLA-C, HLA-DRB1) with population frequency greater than 1% of Chinese population were selected for neoantigen analysis

Since the MHC Class II subtypes are extremely complex due to polymorphisms of both alpha and beta chains, we only studied the binding of EGFR deletions to HLA DRB1 molecules. 378 neo-peptides were identified for HLA Class II HLA DRB1 molecules (Additional file 1: Table S1, Additional file 2: Table S2, Additional file 3: Table S3, Additional file 4: Table S4, Additional file 5: Table S5, Additional file 6: Table S6, Additional file 7: Table S7, Additional file 8: Table S8, Additional file 9: Table S9, Additional file 10: Table S10 and Additional file 11: Table S11). Del19 mutations of EGFR may be presented by HLA DRB1 molecules with a frequency ranging from 16.7 to 81.2%, with delE746_A750 presented by 58.1% of Chinese population.

**Fig. 1:**
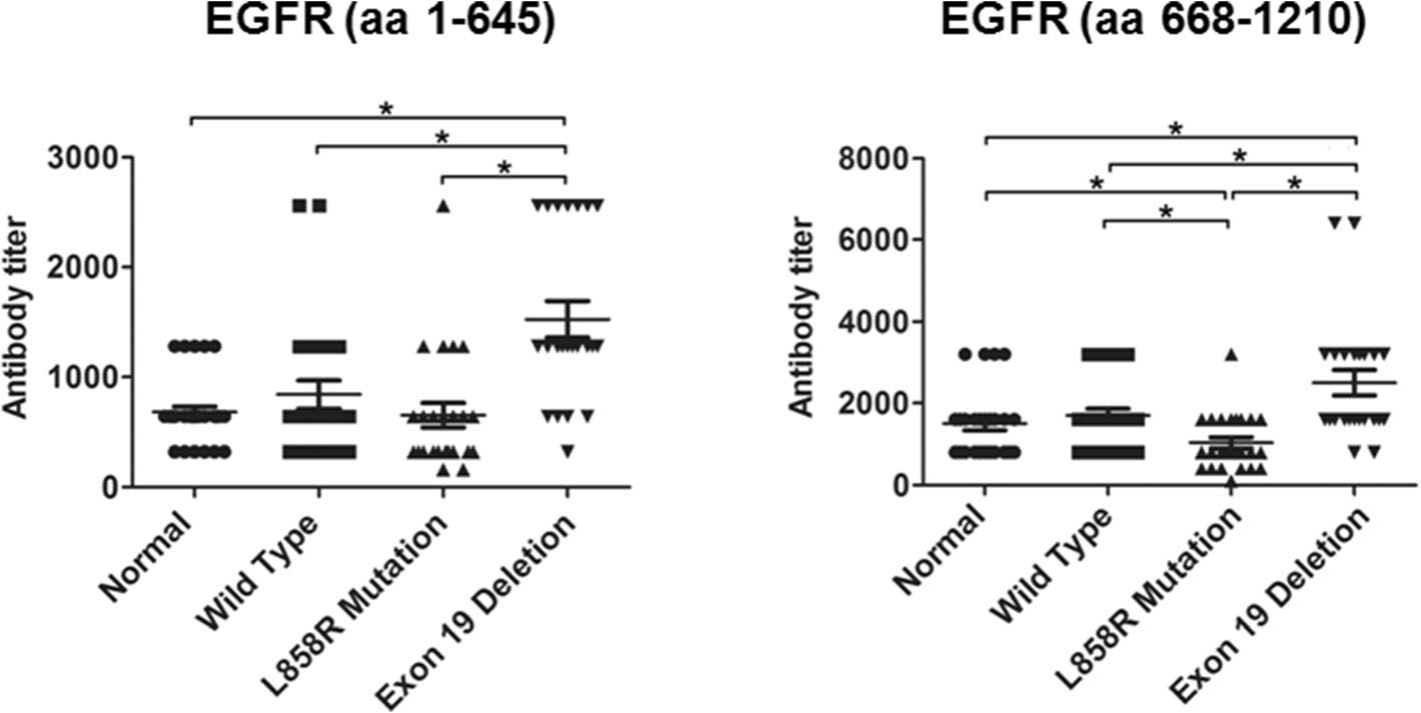
Antibody response to mutant EGFR in lung adenocarcinoma patients. Anti-EGFR antibody titer in serum from lung adenocarcinoma cancer patients was measured by ELISA. Antibody titer was compared among patients with EGFR Exon 19 deletion, EGFR L858 point mutation, EGF wild type lung adenocarcinoma, and healthy individuals. Data were representative of 3 independent experiments. * means *p* < 0.05

Since the serum cross-reactivity to wild type EGFR protein may reflect the immunogenicity of mutant EGFRs, we compared the antibody responses in lung adenocarcinoma patients with EGFR Del19 as that with EGFR L858R point mutation (Table 2). The results showed that the EGFR Del19 mutations triggered significant higher autoantibody response as compared to EGFR L858R point mutation and wild type genotypes. The medium titer of Del19 patients was about 1 fold higher than that of patient with L858R point mutation (Fig. 1).

**Table 2:** Demographics of de-identified patients enrolled in this study

EGFR	Age-ranges (years)	Pathologic subtype	TNM stage	Smoking
L858R mutation	50–60	adenocarcinoma	IV	0
L858R mutation	50–60	adenocarcinoma	IV	300, 30
L858R mutation	70–80	adenocarcinoma	IB	0
L858R mutation	70–80	adenocarcinoma	IV	0
L858R mutation	70–80	adenocarcinoma	IV	1600, 10
L858R mutation	70–80	adenocarcinoma	IB	0
L858R mutation	70–80	adenocarcinoma	IV	450
L858R mutation	60–70	adenocarcinoma	IV	200
L858R mutation	60–70	adenocarcinoma	IV	0
L858R mutation	60–70	adenocarcinoma	IV	0
L858R mutation	50–60	adenocarcinoma	IV	0
L858R mutation	60–70	adenocarcinoma	IV	0
L858R mutation	50–60	adenocarcinoma	IV	0
L858R mutation	80–90	adenocarcinoma	IV	0
L858R mutation	60–70	adenocarcinoma	IV	0
L858R mutation	70–80	adenocarcinoma	IV	0
L858R mutation	40–50	adenocarcinoma	IIA	0
L858R mutation	50–60	adenocarcinoma	IV	0
L858R mutation	60–70	adenocarcinoma	IV	0
L858R mutation	60–70	adenocarcinoma	IV	0
Exon 19 deletion	30–50	adenocarcinoma	IIIB	100
Exon 19 deletion	50–60	adenocarcinoma	IV	0
Exon 19 deletion	60–70	adenocarcinoma	IV	0
Exon 19 deletion	40–50	adenocarcinoma	p-IIIB	0
Exon 19 deletion	50–60	adenocarcinoma	IV	450, 10
Exon 19 deletion	50–60	adenocarcinoma	IV	0
Exon 19 deletion	60–70	adenocarcinoma	IV	300
Exon 19 deletion	60–70	non-small cell lung cancer	IV	140
Exon 19 deletion	60–70	adenocarcinoma	IV	800
Exon 19 deletion	70–80	adenocarcinoma	IV	0
Exon 19 deletion	80–90	adenocarcinoma	IV	0
Exon 19 deletion	60–70	adenocarcinoma	IV	0
Exon 19 deletion	60–70	adenocarcinoma	IV	0
Exon 19 deletion	50–60	adenocarcinoma	IV	0
Exon 19 deletion	70–80	adenocarcinoma	IV	0
Exon 19 deletion	60–70	adenocarcinoma	IV	800, 1
Exon 19 deletion	70–80	adenocarcinoma	IV	1800
Exon 19 deletion	60–70	adenocarcinoma	IV	0
Exon 19 deletion	60–70	adenocarcinoma	cT4N3Mx	0
Exon 19 deletion	40–50	adenocarcinoma	IV	0
Exon 19 deletion	40–50	adenocarcinoma	IV	200
Wild type	60–70	adenocarcinoma	IV	600
Wild type	60–70	adenocarcinoma	IV	2400
Wild type	50–60	mediastinal malignant tumor	?	600
Wild type	60–70	non-small cell lung cancer	IIIB	0
Wild type	50–60	adenocarcinoma	IV	600
Wild type	60–70	small cell lung cancer	IIIB	1000
Wild type	50–60	non-small cell lung cancer	IIIA	0
Wild type	60–70	adenocarcinoma	IV	800
Wild type	60–70	adenocarcinoma	p-IIA	800, 7
Wild type	60–70	small cell lung cancer	IV	800
Wild type	60–70	squamous cell carcinoma	cT4N2Mx	0
Wild type	60–70	small cell lung cancer	IV	0
Wild type	70–80	adenocarcinoma	IIIB	0
Wild type	60–70	non-small cell lung cancer	IIIB	1600
Wild type	60–70	non-small cell lung cancer	cT3N3Mx	1200
Wild type	60–70	adenocarcinoma	IIB	1600, 2
Wild type	60–70	neuroendocrine carcinoma	IV	800
Wild type	70–80	squamous cell carcinoma	IIIA	1200
Wild type	60–70	adenocarcinoma	IV	1200
Wild type	50–60	non-small cell lung cancer	IV	0

*(cigarettes/year), Years after quitting

## Discussion

Immunotherapy targeting PD1 and PDL1 molecules have been approved to treat non-small cell lung carcinoma [21]. Molecular alterations in oncogenes such as EGFR and ALK alterations are associated with a reduced benefit as compared to wild-type patients [22, 23]. EGFR TKI combination with immunotherapy was found to be associated with a high incidence of interstitial lung disease. However, Immunotherapy by PD1 blockade combined with VEGF blockade plus chemotherapy significantly improved progression-free survival (PFS) and overall survival (OS) among patients with metastatic nonsquamous NSCLC with EGFR or ALK genetic alteration status (medium 9.7 months versus 6.1 months), suggesting that immunotherapy is a major area of research for therapy in EGFR-TKI resistant patients [24], especially in East Asian NSCLC patients.

Dong et al. recently analyzed the immunoglical profiles of Chinese NSCLC patients with EGFR mutations [25]. They reported that patients with EGFR mutation showed a lack of T-cell infiltration and shrinking proportion of PD-L1+/CD8+ TIL, suggesting an uninflamed tumor microenvironment. The other finding is that patients with EGFR mutations showed a significantly decreased mutation burden. Thus the induction of tumor-specific T cells is critical for treating Chinese NSCLC patients. Our analysis on Del19 mutations of EGFR, as well as EGFR point mutations, suggest that they may serve as candidate neoantigen peptide vaccines to induce both CD4 and CD8 T cells, a prerequisite for NSCLC patients to benefit from PD1 blockade drugs.

In this study, we found that Del19 mutations triggered significant higher antibody responses as compared to EGFR L858 point mtations in Chinese lung adenocarcinoma patients, suggesting that Del19 mutations of EGFR are expressed at protein level and immunogenic. The presence of autoantibodies toward wild type EGFR was also reported by Azuma et al. in Japanese NSCLC patients treated by EGFR TKI [26]. Together with our results from Chinese NSCLC patients, these data suggest that Del19 mutations of EGFR may serve as excellent candidate targets for immunotherapy as T cell epitopes. Indeed, another type of Del mutation of EGFR, EGFR type III variant (EGFRvIII), which has a deletion in its extracellular domain, has been tested as a vaccine candidate, and triggered both potent antibody responses and T cell responses against tumor cells bearing EGFRvIII mutation [27]. Interestingly, EGFR L858R point mutation did not trigger higher antibody response as compared to EGFR wild type adenocarcinoma patients, nor healthy group. Whether Del19 and L858R mutations may trigger T cell responses remain to be studied. Using X –ray crystallographic analysis, studies have shown that protein conformations of Del19 and L858R are different in both continuous state of kinase activation and conformation upon disruption of dimerization [28]. Other studies also showed Del19 and L858R are different bio-medically such as ability of triggering cell proliferation [29]. Similarly, analysis of clinical data from two large randomized phase III clinical trials of LUX-lung 3 and LUX-lung 6 also demonstrated greater benefit from 2nd generation EGFR-TKI afatinib than standard chemotherapy in patients with EGFR del19 as compared to patients with L858R, indicating potential bio-medical difference between EGFR del19 and L858R [30]. Although the current clinical practice guideline classifies lung adenocarcinoma patients with EGFR del19 and L858R in the same group, our data along with above evidence indicates that these two subtypes may be different not only in biology, but also in immunogenicity. Further studies to understand the difference among these two most common EGFR alteration subtypes are warranted. Although PD1 blockade combined with VEGF blockade and chemotherapy significantly improved PFS and OS among patients with lung adenocarcinomas with EGFR mutations [24, 31], whether or not one subtype benefits more than the other remains un-known. To select the patients who will most likely benefit and avoid the toxicities in patients who are unlikely benefit will be critical.

K-Ras, TP53, and EGFR mutants are well known vaccine candidates which are currently in clinical trials [32–36]. In addition, neo-antigens of passenger mutations are also attractive targets for individualized precision therapy when loaded to dendritic cells or other vaccine formulations. However, it might be technically difficult to determine whether the predicted neo-antigen mutations are presented by MHC Class I molecules. ELISPOT assay by synthetic candidate peptide epitopes may give some clues, but the antigenic epitopes may still show negative results due to loss of neo-antigen, mutations of HLA class I presentation pathway or immune suppression in tumor micro environment. Custom synthesis of pure mutant peptides are required for every patient to perform ELISPOT assay, which is both time consuming and expensive. We are developing ELISPOT assays by 444 neo-peptides identified in Additional file 1: Table S1, Additional file 2: Table S2, Additional file 3: Table S3, Additional file 4: Table S4, Additional file 5: Table S5, Additional file 6: Table S6, Additional file 7: Table S7, Additional file 8: Table S8, Additional file 9: Table S9, Additional file 10: Table S10 and Additional file 11: Table S11, which may be used to predict the function of T cells which destruct tumor cells in cancer patients bearing Del19 mutations of EGFR.

Both CD4 and CD8 epitopes are considered as important for effective anti-tumor immunity. Long peptide vaccines which contain both T cell epitopes for HLA Class I molecules and HLA Class II molecules have shown promise in eliciting both CD4 and CD8 T cell immunity against tumor cells expressing viral or tumor antigens. For example, the long peptide containing E6 and E7 proteins of Human Papilloma virus can be presented by both HLA Class I and II pathways, and showed efficacy in eliciting both CD4 and CD8 T cell responses which reduced tumor burden in cervical cancer patients [37, 38]. The immunogenicity of 40-mer long peptides containing the 11 Del19 mutations of EGFR as a component of cancer vaccine (Table 1) is currently being studied.

During this study, Turajlic et al. reported that neoantigens derived from indel mutations were nine times enriched for mutant specific binding, as compared with non-synonymous SNV derived neoantigens [39]. In the renal clear cell carcinoma cohort, indel mutation was associated with upregulation of antigen presentation genes and T-cell activation as measured by CD8-positive expression. Indel count was significantly associated with checkpoint inhibitor response across three separate melanoma cohorts. The immune responses in patients bearing Del19 mutations of EGFR treated by checkpoint inhibitor remain to be studied, as well as EGFR L858R mutation (Additional file 12).

## Conclusions

Our results showed that serum reactivity to wild type EGFR protein was significantly higher in patients with Del19 EGFR mutations than those with EGFR L858R point mutation or with EGFR wild type genotype. These findings suggest that Del19 mutations of EGFR, with an estimated frequency of 40% in Chinese lung adenocarcinoma patients, may serve as unique targets for immunotherapy in Chinese lung cancer patients.

## Methods

### Neo-peptides prediction

29-mer polypeptides centered on mutated residues were scanned to identify candidate peptides binding to HLA class I or II, i.e., peptide sequences surrounding mutated amino acids resulting from Del19 and L858R EGFR mutations. The affinity of 8–12 mer peptides binding to HLA class I were predicted using the NetMHCPan4.0 binding algorithm [19]. The affinity of 15 mer peptides binding to HLA class II were predicted using the NetMHCIIPan3.1 binding algorithm. Threshold of binding affinity was defined as IC50 < 500 nM. HLA alleles (HLA-A, HLA-B, HLA-C, and HLA-DRB1) with frequency greater than 1% of Chinese population were selected for neoantigen analysis. HLA allele frequency for Chinese population [21] was according to http://www.allelefrequencies.net.

### Elisa

Serum from lung adenocarcinoma cancer patients treated in Shanghai Pulmonary Hospital were banked according to protocols approved by Institutional Review Board (K16–245-1). EGFR mutations were detected by ARMS method using ADx *EGFR* Mutations Detection Kit (Amoy Diagnostics, Xiamen, China) [40].

The recombinant EGFR protein (extracellular part aa 1–645 and intracellular part aa 668–1210) was from Sinobiologicals, China. The EGFR protein was bound to ELISA plates (1 μg/ml) for overnight at 4 °C. 100 μl serum of lung cancer patients were added and incubated for 1 h at room temperature. The plates were washed for 3 times by washing buffer (PBS with 0.05% Tween-20), and incubated with HRP labeled goat anti-human IgG for 1 h, followed by colorimetric detection. PBS 1% BSA was used as blank for determining the cutoff value.

## Supplementary information

**Additional file 1.** Predicted HLA binding epitopes for EGFR delE746_A750.

**Additional file 2.** Predicted HLA binding epitopes for EGFR delL747_P753insS.

**Additional file 3.** Predicted HLA binding epitopes for EGFR delL747_T751.

**Additional file 4.** Predicted HLA binding epitopes for EGFR delL747_A750insP.

**Additional file 5.** Predicted HLA binding epitopes for EGFR delL747_S752.

**Additional file 6.** Predicted HLA binding epitopes for EGFR delE746_S752insV.

**Additional file 7.** Predicted HLA binding epitopes for EGFR delE746_P753insVS.

**Additional file 8.** Predicted HLA binding epitopes for EGFR delL747_T751insP.

**Additional file 9.** Predicted HLA binding epitopes for EGFR delE746_T751insA.

**Additional file 10.** Predicted HLA binding epitopes for EGFR delL747_P753.

**Additional file 11.** Predicted HLA binding epitopes for EGFR delS752_I759.

**Additional file 12.** Comparison between EGFR exon Del 19 and EGFR L858R derived peptides.

## Data Availability

The dataset of the current study is available from the corresponding author at a reasonable request.

## Abbreviations

ARMS: amplification refractory mutation system
COSMIC: Catalog Of Somatic Mutations In Cancer
DC: dendritic cell
EGFR: epidermal growth factor receptor
ELISPOT: Enzyme-linked Immunospot
GM-CSF: granulocyte–macrophage colony-stimulating factor
HLA: human leukocyte antigen
HRP: horse radish peroxidase
MHC: major histocompatibility complex
