# Supplementary material for: Immunogenicity of Del19 EGFR mutations in Chinese patients affected by lung adenocarcinoma
Source: BMC Immunol. 2019 Nov 13;20:43. doi: 10.1186/s12865-019-0320-1 (PMC6854806; doi:10.1186/s12865-019-0320-1)
Supplement: Supplementary file 1 — Additional file 1. Predicted HLA binding epitopes for EGFR delE746_A750. [file 12865_2019_320_MOESM1_ESM.doc]

**Supplemental Table 1, Predicted HLA binding epitopes for EGFR delE746_A750 by Chinese NSCLC patients as predicted by NetMHC4.0.** The percentages are the total frequencies of HLA alleles which may present a mutant EGFR.

| Class I | | | Class II | | |
| --- | --- | --- | --- | --- | --- |
| Neopeptide | HLA alleles | Frequency | Neopeptide | HLA alleles | Frequency |
| IPVAIKTSPK | HLA-B*55 | 0.00% | KIPVAIKTSPKANK | DRB1_01 | 6.04% |
| IPVAIKTSPK | HLA-A*68 | 0.85% | KIPVAIKTSPKANK | DRB1_03 | 0.00% |
| IPVAIKTSPK | HLA-A*34 | 0.00% | KIPVAIKTSPKANK | DRB1_04 | 0.00% |
| IPVAIKTSPK | HLA-A*30 | 0.00% | KIPVAIKTSPKANK | DRB1_08 | 16.05% |
| IPVAIKTSPK | HLA-A*11 | 25.85% | KIPVAIKTSPKANK | DRB1_09 | 0.00% |
| IPVAIKTSPK | HLA-A*03 | 3.27% | KIPVAIKTSPKANK | DRB1_11 | 8.11% |
| AIKTSPKANK | HLA-A*68 | 0.00% | KIPVAIKTSPKANK | DRB1_12 | 1.90% |
| AIKTSPKANK | HLA-A*30 | 7.56% | KIPVAIKTSPKANK | DRB1_13 | 0.00% |
| AIKTSPKANK | HLA-A*11 | 24.34% | KIPVAIKTSPKANK | DRB1_14 | 19.14% |
| AIKTSPKANK | HLA-A*03 | 2.88% | KIPVAIKTSPKANK | DRB1_15 | 0.00% |
| KIPVAIKTSPK | HLA-A*30 | 0.00% | KIPVAIKTSPKANK | DRB1_16 | 6.81% |
| KIPVAIKTSPK | HLA-A*11 | 0.66% | IPVAIKTSPKANKE | DRB1_01 | 6.04% |
| KIPVAIKTSPK | HLA-A*03 | 2.88% | IPVAIKTSPKANKE | DRB1_03 | 0.00% |
| KVKIPVAIKT | HLA-A*30 | 7.56% | IPVAIKTSPKANKE | DRB1_04 | 0.00% |
| KVKIPVAIKT | HLA-A*03 | 0.00% | IPVAIKTSPKANKE | DRB1_08 | 16.05% |
| PVAIKTSPK | HLA-A*68 | 0.00% | IPVAIKTSPKANKE | DRB1_09 | 0.00% |
| PVAIKTSPK | HLA-A*34 | 0.00% | IPVAIKTSPKANKE | DRB1_11 | 8.11% |
| PVAIKTSPK | HLA-A*11 | 0.46% | IPVAIKTSPKANKE | DRB1_12 | 1.90% |
| KTSPKANK | HLA-A*30 | 0.00% | IPVAIKTSPKANKE | DRB1_13 | 0.00% |
| KTSPKANK | HLA-A*03 | 0.00% | IPVAIKTSPKANKE | DRB1_14 | 13.50% |
| KTSPKANKEI | HLA-B*15 | 0.00% | IPVAIKTSPKANKE | DRB1_16 | 0.00% |
|  |  |  | IPVAIKTSPKANK | DRB1_01 | 6.04% |
|  |  |  | IPVAIKTSPKANK | DRB1_04 | 0.00% |
|  |  |  | IPVAIKTSPKANK | DRB1_08 | 16.05% |
|  |  |  | IPVAIKTSPKANK | DRB1_09 | 0.00% |
|  |  |  | IPVAIKTSPKANK | DRB1_11 | 8.11% |
|  |  |  | IPVAIKTSPKANK | DRB1_12 | 1.90% |
|  |  |  | IPVAIKTSPKANK | DRB1_13 | 0.00% |
|  |  |  | IPVAIKTSPKANK | DRB1_14 | 13.50% |
|  |  |  | IPVAIKTSPKANK | DRB1_16 | 0.00% |
|  |  |  | VKIPVAIKTSPKAN | DRB1_01 | 6.04% |
|  |  |  | VKIPVAIKTSPKAN | DRB1_04 | 0.00% |
|  |  |  | VKIPVAIKTSPKAN | DRB1_08 | 16.05% |
|  |  |  | VKIPVAIKTSPKAN | DRB1_09 | 0.00% |
|  |  |  | VKIPVAIKTSPKAN | DRB1_11 | 8.11% |
|  |  |  | VKIPVAIKTSPKAN | DRB1_12 | 1.90% |
|  |  |  | VKIPVAIKTSPKAN | DRB1_13 | 0.00% |
|  |  |  | VKIPVAIKTSPKAN | DRB1_14 | 13.50% |
|  |  |  | VKIPVAIKTSPKAN | DRB1_15 | 0.00% |
|  |  |  | VKIPVAIKTSPKAN | DRB1_16 | 0.00% |
|  |  |  | PVAIKTSPKANKEI | DRB1_01 | 2.02% |
|  |  |  | PVAIKTSPKANKEI | DRB1_03 | 0.00% |
|  |  |  | PVAIKTSPKANKEI | DRB1_04 | 0.00% |
|  |  |  | PVAIKTSPKANKEI | DRB1_08 | 16.05% |
|  |  |  | PVAIKTSPKANKEI | DRB1_09 | 0.00% |
|  |  |  | PVAIKTSPKANKEI | DRB1_11 | 8.11% |
|  |  |  | PVAIKTSPKANKEI | DRB1_12 | 1.90% |
|  |  |  | PVAIKTSPKANKEI | DRB1_13 | 0.00% |
|  |  |  | PVAIKTSPKANKEI | DRB1_14 | 13.50% |
|  |  |  | PVAIKTSPKANKEI | DRB1_16 | 0.00% |
|  |  |  | KIPVAIKTSPKAN | DRB1_01 | 6.04% |
|  |  |  | KIPVAIKTSPKAN | DRB1_04 | 0.00% |
|  |  |  | KIPVAIKTSPKAN | DRB1_08 | 16.05% |
|  |  |  | KIPVAIKTSPKAN | DRB1_09 | 0.00% |
|  |  |  | KIPVAIKTSPKAN | DRB1_11 | 8.11% |
|  |  |  | KIPVAIKTSPKAN | DRB1_12 | 1.90% |
|  |  |  | KIPVAIKTSPKAN | DRB1_13 | 0.00% |
|  |  |  | KIPVAIKTSPKAN | DRB1_14 | 13.50% |
|  |  |  | KIPVAIKTSPKAN | DRB1_16 | 0.00% |
|  |  |  | KVKIPVAIKTSPKA | DRB1_01 | 6.04% |
|  |  |  | KVKIPVAIKTSPKA | DRB1_04 | 0.00% |
|  |  |  | KVKIPVAIKTSPKA | DRB1_08 | 4.92% |
|  |  |  | KVKIPVAIKTSPKA | DRB1_09 | 0.00% |
|  |  |  | KVKIPVAIKTSPKA | DRB1_11 | 8.11% |
|  |  |  | KVKIPVAIKTSPKA | DRB1_12 | 1.90% |
|  |  |  | KVKIPVAIKTSPKA | DRB1_13 | 0.00% |
|  |  |  | KVKIPVAIKTSPKA | DRB1_14 | 13.50% |
|  |  |  | KVKIPVAIKTSPKA | DRB1_15 | 0.00% |
|  |  |  | KVKIPVAIKTSPKA | DRB1_16 | 0.00% |
|  |  |  | VAIKTSPKANKEIL | DRB1_01 | 2.02% |
|  |  |  | VAIKTSPKANKEIL | DRB1_03 | 0.00% |
|  |  |  | VAIKTSPKANKEIL | DRB1_04 | 0.00% |
|  |  |  | VAIKTSPKANKEIL | DRB1_08 | 4.92% |
|  |  |  | VAIKTSPKANKEIL | DRB1_09 | 0.00% |
|  |  |  | VAIKTSPKANKEIL | DRB1_11 | 8.11% |
|  |  |  | VAIKTSPKANKEIL | DRB1_12 | 1.90% |
|  |  |  | VAIKTSPKANKEIL | DRB1_13 | 0.00% |
|  |  |  | VAIKTSPKANKEIL | DRB1_14 | 13.50% |
|  |  |  | PVAIKTSPKANKE | DRB1_01 | 2.02% |
|  |  |  | PVAIKTSPKANKE | DRB1_03 | 0.00% |
|  |  |  | PVAIKTSPKANKE | DRB1_04 | 0.00% |
|  |  |  | PVAIKTSPKANKE | DRB1_08 | 4.92% |
|  |  |  | PVAIKTSPKANKE | DRB1_09 | 0.00% |
|  |  |  | PVAIKTSPKANKE | DRB1_11 | 8.11% |
|  |  |  | PVAIKTSPKANKE | DRB1_12 | 1.90% |
|  |  |  | PVAIKTSPKANKE | DRB1_13 | 0.00% |
|  |  |  | PVAIKTSPKANKE | DRB1_14 | 13.50% |
|  |  |  | VAIKTSPKANKEI | DRB1_01 | 2.02% |
|  |  |  | VAIKTSPKANKEI | DRB1_03 | 0.00% |
|  |  |  | VAIKTSPKANKEI | DRB1_08 | 4.92% |
|  |  |  | VAIKTSPKANKEI | DRB1_11 | 8.11% |
|  |  |  | VAIKTSPKANKEI | DRB1_12 | 1.90% |
|  |  |  | VAIKTSPKANKEI | DRB1_13 | 0.00% |
|  |  |  | VAIKTSPKANKEI | DRB1_14 | 13.50% |
|  |  |  | PVAIKTSPKANK | DRB1_01 | 2.02% |
|  |  |  | PVAIKTSPKANK | DRB1_08 | 4.92% |
|  |  |  | PVAIKTSPKANK | DRB1_11 | 8.11% |
|  |  |  | PVAIKTSPKANK | DRB1_12 | 1.90% |
|  |  |  | PVAIKTSPKANK | DRB1_13 | 0.00% |
|  |  |  | PVAIKTSPKANK | DRB1_14 | 13.50% |
|  |  |  | GEKVKIPVAIKTS | DRB1_01 | 6.04% |
|  |  |  | EGEKVKIPVAIKTS | DRB1_01 | 6.04% |
|  |  |  | GEKVKIPVAIKTS | DRB1_08 | 4.92% |
|  |  |  | EGEKVKIPVAIKTS | DRB1_08 | 4.92% |
|  |  |  | GEKVKIPVAIKTS | DRB1_09 | 0.00% |
|  |  |  | EGEKVKIPVAIKTS | DRB1_09 | 0.00% |
|  |  |  | GEKVKIPVAIKTS | DRB1_11 | 8.11% |
|  |  |  | EGEKVKIPVAIKTS | DRB1_11 | 8.11% |
|  |  |  | GEKVKIPVAIKTS | DRB1_12 | 1.90% |
|  |  |  | EGEKVKIPVAIKTS | DRB1_12 | 1.90% |
|  |  |  | GEKVKIPVAIKTS | DRB1_13 | 0.00% |
|  |  |  | EGEKVKIPVAIKTS | DRB1_13 | 0.00% |
|  |  |  | GEKVKIPVAIKTS | DRB1_14 | 13.50% |
|  |  |  | EGEKVKIPVAIKTS | DRB1_14 | 13.50% |
|  |  |  | GEKVKIPVAIKTS | DRB1_16 | 0.00% |
|  |  |  | VKIPVAIKTSPKA | DRB1_01 | 2.02% |
|  |  |  | VKIPVAIKTSPKA | DRB1_08 | 4.92% |
|  |  |  | VKIPVAIKTSPKA | DRB1_09 | 0.00% |
|  |  |  | VKIPVAIKTSPKA | DRB1_11 | 8.11% |
|  |  |  | VKIPVAIKTSPKA | DRB1_12 | 1.90% |
|  |  |  | VKIPVAIKTSPKA | DRB1_13 | 0.00% |
|  |  |  | VKIPVAIKTSPKA | DRB1_14 | 13.50% |
|  |  |  | IPVAIKTSPKAN | DRB1_01 | 2.02% |
|  |  |  | IPVAIKTSPKAN | DRB1_08 | 4.92% |
|  |  |  | IPVAIKTSPKAN | DRB1_11 | 8.11% |
|  |  |  | IPVAIKTSPKAN | DRB1_12 | 1.90% |
|  |  |  | IPVAIKTSPKAN | DRB1_13 | 0.00% |
|  |  |  | IPVAIKTSPKAN | DRB1_14 | 13.50% |
|  |  |  | EKVKIPVAIKTSPK | DRB1_01 | 2.02% |
|  |  |  | EKVKIPVAIKTSPK | DRB1_08 | 4.92% |
|  |  |  | EKVKIPVAIKTSPK | DRB1_09 | 0.00% |
|  |  |  | EKVKIPVAIKTSPK | DRB1_11 | 2.57% |
|  |  |  | EKVKIPVAIKTSPK | DRB1_12 | 1.90% |
|  |  |  | EKVKIPVAIKTSPK | DRB1_13 | 0.00% |
|  |  |  | EKVKIPVAIKTSPK | DRB1_14 | 13.50% |
|  |  |  | GEKVKIPVAIKTSP | DRB1_01 | 2.02% |
|  |  |  | GEKVKIPVAIKTSP | DRB1_08 | 4.92% |
|  |  |  | GEKVKIPVAIKTSP | DRB1_09 | 0.00% |
|  |  |  | GEKVKIPVAIKTSP | DRB1_11 | 2.57% |
|  |  |  | GEKVKIPVAIKTSP | DRB1_12 | 1.90% |
|  |  |  | GEKVKIPVAIKTSP | DRB1_13 | 0.00% |
|  |  |  | GEKVKIPVAIKTSP | DRB1_14 | 13.50% |
|  |  |  | KIPVAIKTSPKA | DRB1_01 | 2.02% |
|  |  |  | KIPVAIKTSPKA | DRB1_08 | 4.92% |
|  |  |  | KIPVAIKTSPKA | DRB1_11 | 2.57% |
|  |  |  | KIPVAIKTSPKA | DRB1_12 | 1.90% |
|  |  |  | KIPVAIKTSPKA | DRB1_13 | 0.00% |
|  |  |  | KIPVAIKTSPKA | DRB1_14 | 13.50% |
|  |  |  | EKVKIPVAIKTS | DRB1_01 | 2.02% |
|  |  |  | EKVKIPVAIKTS | DRB1_08 | 4.92% |
|  |  |  | EKVKIPVAIKTS | DRB1_09 | 0.00% |
|  |  |  | EKVKIPVAIKTS | DRB1_11 | 2.57% |
|  |  |  | EKVKIPVAIKTS | DRB1_12 | 1.90% |
|  |  |  | EKVKIPVAIKTS | DRB1_13 | 0.00% |
|  |  |  | EKVKIPVAIKTS | DRB1_14 | 12.37% |
|  |  |  | VAIKTSPKANKE | DRB1_01 | 2.02% |
|  |  |  | EKVKIPVAIKTSP | DRB1_01 | 2.02% |
|  |  |  | VAIKTSPKANKE | DRB1_08 | 4.92% |
|  |  |  | EKVKIPVAIKTSP | DRB1_08 | 4.92% |
|  |  |  | EKVKIPVAIKTSP | DRB1_09 | 0.00% |
|  |  |  | VAIKTSPKANKE | DRB1_11 | 2.57% |
|  |  |  | EKVKIPVAIKTSP | DRB1_11 | 2.57% |
|  |  |  | VAIKTSPKANKE | DRB1_12 | 1.90% |
|  |  |  | EKVKIPVAIKTSP | DRB1_12 | 1.90% |
|  |  |  | VAIKTSPKANKE | DRB1_13 | 0.00% |
|  |  |  | EKVKIPVAIKTSP | DRB1_13 | 0.00% |
|  |  |  | VAIKTSPKANKE | DRB1_14 | 12.37% |
|  |  |  | EKVKIPVAIKTSP | DRB1_14 | 12.37% |
|  |  |  | KVKIPVAIKTSPK | DRB1_01 | 2.02% |
|  |  |  | KVKIPVAIKTSPK | DRB1_08 | 4.92% |
|  |  |  | KVKIPVAIKTSPK | DRB1_11 | 2.57% |
|  |  |  | KVKIPVAIKTSPK | DRB1_12 | 1.90% |
|  |  |  | KVKIPVAIKTSPK | DRB1_13 | 0.00% |
|  |  |  | KVKIPVAIKTSPK | DRB1_14 | 5.38% |
|  |  |  | KVKIPVAIKTSP | DRB1_01 | 2.02% |
|  |  |  | KVKIPVAIKTSP | DRB1_08 | 4.92% |
|  |  |  | KVKIPVAIKTSP | DRB1_11 | 2.57% |
|  |  |  | KVKIPVAIKTSP | DRB1_12 | 1.90% |
|  |  |  | KVKIPVAIKTSP | DRB1_13 | 0.00% |
|  |  |  | KVKIPVAIKTSP | DRB1_14 | 5.38% |
|  |  |  | VAIKTSPKANK | DRB1_01 | 0.00% |
|  |  |  | AIKTSPKANKEIL | DRB1_03 | 0.00% |
|  |  |  | VAIKTSPKANK | DRB1_08 | 4.92% |
|  |  |  | AIKTSPKANKEIL | DRB1_08 | 4.92% |
|  |  |  | VAIKTSPKANK | DRB1_11 | 2.57% |
|  |  |  | AIKTSPKANKEIL | DRB1_11 | 2.57% |
|  |  |  | VAIKTSPKANK | DRB1_12 | 0.00% |
|  |  |  | AIKTSPKANKEIL | DRB1_12 | 0.00% |
|  |  |  | VAIKTSPKANK | DRB1_13 | 0.00% |
|  |  |  | AIKTSPKANKEIL | DRB1_13 | 0.00% |
|  |  |  | AIKTSPKANKEIL | DRB1_14 | 12.37% |
|  |  |  | VAIKTSPKANK | DRB1_14 | 5.38% |
|  |  |  | KVKIPVAIKTS | DRB1_01 | 2.02% |
|  |  |  | AIKTSPKANKEILD | DRB1_03 | 0.00% |
|  |  |  | KVKIPVAIKTS | DRB1_08 | 3.69% |
|  |  |  | AIKTSPKANKEILD | DRB1_08 | 4.92% |
|  |  |  | KVKIPVAIKTS | DRB1_11 | 2.57% |
|  |  |  | AIKTSPKANKEILD | DRB1_11 | 2.57% |
|  |  |  | KVKIPVAIKTS | DRB1_12 | 1.90% |
|  |  |  | AIKTSPKANKEILD | DRB1_12 | 0.00% |
|  |  |  | AIKTSPKANKEILD | DRB1_13 | 0.00% |
|  |  |  | KVKIPVAIKTS | DRB1_13 | 0.00% |
|  |  |  | AIKTSPKANKEILD | DRB1_14 | 12.37% |
|  |  |  | KVKIPVAIKTS | DRB1_14 | 5.38% |
|  |  |  | PVAIKTSPKAN | DRB1_01 | 2.02% |
|  |  |  | PVAIKTSPKAN | DRB1_08 | 4.92% |
|  |  |  | PVAIKTSPKAN | DRB1_11 | 2.57% |
|  |  |  | PVAIKTSPKAN | DRB1_12 | 0.00% |
|  |  |  | PVAIKTSPKAN | DRB1_13 | 0.00% |
|  |  |  | PVAIKTSPKAN | DRB1_14 | 5.38% |
|  |  |  | IPVAIKTSPKA | DRB1_01 | 0.00% |
|  |  |  | IPVAIKTSPKA | DRB1_08 | 4.92% |
|  |  |  | IPVAIKTSPKA | DRB1_11 | 2.57% |
|  |  |  | IPVAIKTSPKA | DRB1_12 | 0.00% |
|  |  |  | IPVAIKTSPKA | DRB1_13 | 0.00% |
|  |  |  | IPVAIKTSPKA | DRB1_14 | 5.38% |
|  |  |  | AIKTSPKANKEI | DRB1_08 | 1.23% |
|  |  |  | AIKTSPKANKEI | DRB1_11 | 2.57% |
|  |  |  | AIKTSPKANKEI | DRB1_12 | 0.00% |
|  |  |  | AIKTSPKANKEI | DRB1_13 | 0.00% |
|  |  |  | AIKTSPKANKEI | DRB1_14 | 5.38% |
|  |  |  | VKIPVAIKTSPK | DRB1_08 | 2.29% |
|  |  |  | VKIPVAIKTSPK | DRB1_11 | 2.57% |
|  |  |  | VKIPVAIKTSPK | DRB1_12 | 0.00% |
|  |  |  | VKIPVAIKTSPK | DRB1_13 | 0.00% |
|  |  |  | VKIPVAIKTSPK | DRB1_14 | 5.38% |
|  |  |  | AIKTSPKANKE | DRB1_08 | 0.00% |
|  |  |  | AIKTSPKANKE | DRB1_11 | 2.57% |
|  |  |  | AIKTSPKANKE | DRB1_13 | 0.00% |
|  |  |  | AIKTSPKANKE | DRB1_14 | 5.38% |
|  |  |  | PVAIKTSPKA | DRB1_08 | 2.29% |
|  |  |  | PVAIKTSPKA | DRB1_11 | 0.00% |
|  |  |  | PVAIKTSPKA | DRB1_13 | 0.00% |
|  |  |  | PVAIKTSPKA | DRB1_14 | 0.00% |
|  |  |  | IKTSPKANKEIL | DRB1_08 | 0.00% |
|  |  |  | IKTSPKANKEIL | DRB1_11 | 0.00% |
|  |  |  | IKTSPKANKEIL | DRB1_13 | 0.00% |
|  |  |  | IKTSPKANKEIL | DRB1_14 | 3.71% |
|  |  |  | IKTSPKANKEILD | DRB1_08 | 0.00% |
|  |  |  | IKTSPKANKEILD | DRB1_11 | 0.00% |
|  |  |  | IKTSPKANKEILD | DRB1_13 | 0.00% |
|  |  |  | IKTSPKANKEILD | DRB1_14 | 3.71% |
|  |  |  | IKTSPKANKEILDE | DRB1_08 | 0.00% |
|  |  |  | IKTSPKANKEILDE | DRB1_11 | 0.00% |
|  |  |  | IKTSPKANKEILDE | DRB1_13 | 0.00% |
|  |  |  | IKTSPKANKEILDE | DRB1_14 | 3.71% |
|  |  |  | VAIKTSPKAN | DRB1_08 | 0.00% |
|  |  |  | KIPVAIKTSPK | DRB1_08 | 0.00% |
|  |  |  | VAIKTSPKAN | DRB1_13 | 0.00% |
|  |  |  | KIPVAIKTSPK | DRB1_13 | 0.00% |
|  |  |  | VAIKTSPKAN | DRB1_14 | 0.00% |
|  |  |  | KIPVAIKTSPK | DRB1_14 | 0.00% |
|  |  |  | AIKTSPKANK | DRB1_08 | 0.00% |
|  |  |  | AIKTSPKANK | DRB1_13 | 0.00% |
|  |  |  | AIKTSPKANK | DRB1_14 | 0.00% |
|  |  |  | VKIPVAIKTSP | DRB1_08 | 0.00% |
|  |  |  | VKIPVAIKTSP | DRB1_13 | 0.00% |
|  |  |  | VKIPVAIKTSP | DRB1_14 | 0.00% |
|  |  |  | VAIKTSPKA | DRB1_08 | 0.00% |
|  |  |  | VKIPVAIKTS | DRB1_08 | 0.00% |
|  |  |  | IKTSPKANKEI | DRB1_08 | 0.00% |
|  |  |  | IPVAIKTSPK | DRB1_08 | 0.00% |
| Total |  | 37.53% |  |  | 58.05% |
