# Supplementary material for: Immunogenicity of Del19 EGFR mutations in Chinese patients affected by lung adenocarcinoma
Source: BMC Immunol. 2019 Nov 13;20:43. doi: 10.1186/s12865-019-0320-1 (PMC6854806; doi:10.1186/s12865-019-0320-1)
Supplement: Supplementary file 2 — Additional file 2. Predicted HLA binding epitopes for EGFR delL747_P753insS. [file 12865_2019_320_MOESM2_ESM.doc]

**Supplemental Table 2, Predicted HLA binding epitopes for EGFR delL747_P753insS by Chinese NSCLC patients as predicted by NetMHC4.0.** The percentages are the total frequencies of HLA alleles which may present a mutant EGFR.

| Class I | | | Class II | | |
| --- | --- | --- | --- | --- | --- |
| Neopeptide | HLA alleles | Frequency | Neopeptide | HLA alleles | Frequency |
| KIPVAIKESK | HLA-A*68 | 0.10% | GEKVKIPVAIKESK | DRB1_01 | 2.02% |
| KIPVAIKESK | HLA-A*31 | 0.00% | GEKVKIPVAIKESK | DRB1_08 | 4.92% |
| KIPVAIKESK | HLA-A*30 | 0.00% | GEKVKIPVAIKESK | DRB1_11 | 8.11% |
| KIPVAIKESK | HLA-A*11 | 24.34% | GEKVKIPVAIKESK | DRB1_12 | 1.90% |
| KIPVAIKESK | HLA-A*03 | 2.88% | GEKVKIPVAIKESK | DRB1_13 | 0.00% |
| KESKANKEI | HLA-B*44 | 0.00% | GEKVKIPVAIKESK | DRB1_14 | 13.50% |
| KESKANKEI | HLA-B*41 | 0.09% | EKVKIPVAIKESKA | DRB1_01 | 2.02% |
| KESKANKEI | HLA-B*40 | 12.82% | EKVKIPVAIKESKA | DRB1_08 | 4.92% |
| KESKANKEI | HLA-B*407 | 0.00% | EKVKIPVAIKESKA | DRB1_11 | 2.57% |
| KESKANKEI | HLA-B*406 | 0.00% | EKVKIPVAIKESKA | DRB1_12 | 1.90% |
| KESKANKEI | HLA-B*405 | 0.00% | EKVKIPVAIKESKA | DRB1_13 | 0.00% |
| KESKANKEI | HLA-B*401 | 0.00% | EKVKIPVAIKESKA | DRB1_14 | 13.50% |
| KESKANKEI | HLA-B*400 | 0.00% | EKVKIPVAIKESK | DRB1_01 | 2.02% |
| KESKANKEI | HLA-B*409 | 0.00% | EKVKIPVAIKESK | DRB1_08 | 4.92% |
| KESKANKEI | HLA-B*408 | 0.00% | EKVKIPVAIKESK | DRB1_11 | 2.57% |
| KESKANKEI | HLA-B*404 | 0.00% | EKVKIPVAIKESK | DRB1_12 | 1.90% |
| KESKANKEI | HLA-B*403 | 0.00% | EKVKIPVAIKESK | DRB1_13 | 0.00% |
| KESKANKEI | HLA-B*402 | 0.00% | EKVKIPVAIKESK | DRB1_14 | 12.37% |
| AIKESKANK | HLA-A*68 | 0.00% | KVKIPVAIKESKAN | DRB1_01 | 2.02% |
| AIKESKANK | HLA-A*31 | 0.00% | KVKIPVAIKESKAN | DRB1_08 | 4.92% |
| AIKESKANK | HLA-A*30 | 7.56% | KVKIPVAIKESKAN | DRB1_11 | 2.57% |
| AIKESKANK | HLA-A*11 | 0.66% | KVKIPVAIKESKAN | DRB1_12 | 1.90% |
| AIKESKANK | HLA-A*03 | 2.88% | KVKIPVAIKESKAN | DRB1_13 | 0.00% |
| KESKANKEIL | HLA-B*40 | 11.73% | KVKIPVAIKESKAN | DRB1_14 | 5.38% |
| KESKANKEIL | HLA-B*407 | 0.00% | KVKIPVAIKESKA | DRB1_01 | 2.02% |
| KESKANKEIL | HLA-B*406 | 0.00% | KVKIPVAIKESKA | DRB1_08 | 4.92% |
| KESKANKEIL | HLA-B*405 | 0.00% | KVKIPVAIKESKA | DRB1_11 | 2.57% |
| KESKANKEIL | HLA-B*401 | 0.00% | KVKIPVAIKESKA | DRB1_12 | 1.90% |
| KESKANKEIL | HLA-B*400 | 0.00% | KVKIPVAIKESKA | DRB1_13 | 0.00% |
| KESKANKEIL | HLA-B*409 | 0.00% | KVKIPVAIKESKA | DRB1_14 | 5.38% |
| KESKANKEIL | HLA-B*408 | 0.00% | KVKIPVAIKESK | DRB1_01 | 0.00% |
| KESKANKEIL | HLA-B*404 | 0.00% | KVKIPVAIKESK | DRB1_08 | 3.69% |
| KESKANKEIL | HLA-B*403 | 0.00% | KVKIPVAIKESK | DRB1_11 | 2.57% |
| KESKANKEIL | HLA-B*402 | 0.00% | KVKIPVAIKESK | DRB1_12 | 1.90% |
| IPVAIKESKA | HLA-B*55 | 2.97% | KVKIPVAIKESK | DRB1_13 | 0.00% |
| IPVAIKESKA | HLA-B*54 | 3.16% | KVKIPVAIKESK | DRB1_14 | 5.38% |
| VAIKESKANK | HLA-A*68 | 0.10% | PVAIKESKANKEIL | DRB1_01 | 2.02% |
| VAIKESKANK | HLA-A*11 | 0.00% | PVAIKESKANKEIL | DRB1_07 | 0.00% |
| VAIKESKANK | HLA-A*03 | 0.00% | PVAIKESKANKEIL | DRB1_08 | 0.00% |
|  |  |  | PVAIKESKANKEIL | DRB1_09 | 0.00% |
|  |  |  | PVAIKESKANKEIL | DRB1_11 | 2.57% |
|  |  |  | PVAIKESKANKEIL | DRB1_12 | 0.00% |
|  |  |  | PVAIKESKANKEIL | DRB1_13 | 0.00% |
|  |  |  | PVAIKESKANKEIL | DRB1_14 | 5.38% |
|  |  |  | IPVAIKESKANKEI | DRB1_01 | 2.02% |
|  |  |  | IPVAIKESKANKEI | DRB1_08 | 0.00% |
|  |  |  | IPVAIKESKANKEI | DRB1_09 | 0.00% |
|  |  |  | IPVAIKESKANKEI | DRB1_11 | 2.57% |
|  |  |  | IPVAIKESKANKEI | DRB1_12 | 0.00% |
|  |  |  | IPVAIKESKANKEI | DRB1_13 | 0.00% |
|  |  |  | IPVAIKESKANKEI | DRB1_131 | 0.00% |
|  |  |  | IPVAIKESKANKEI | DRB1_14 | 5.38% |
|  |  |  | VAIKESKANKEIL | DRB1_01 | 2.02% |
|  |  |  | VAIKESKANKEIL | DRB1_08 | 0.00% |
|  |  |  | VAIKESKANKEIL | DRB1_09 | 0.00% |
|  |  |  | VAIKESKANKEIL | DRB1_11 | 2.57% |
|  |  |  | VAIKESKANKEIL | DRB1_12 | 0.00% |
|  |  |  | VAIKESKANKEIL | DRB1_13 | 0.00% |
|  |  |  | VAIKESKANKEIL | DRB1_131 | 0.00% |
|  |  |  | VAIKESKANKEIL | DRB1_14 | 5.38% |
|  |  |  | VKIPVAIKESKANK | DRB1_01 | 0.00% |
|  |  |  | VKIPVAIKESKANK | DRB1_08 | 0.00% |
|  |  |  | VKIPVAIKESKANK | DRB1_11 | 2.57% |
|  |  |  | VKIPVAIKESKANK | DRB1_12 | 0.00% |
|  |  |  | VKIPVAIKESKANK | DRB1_13 | 0.00% |
|  |  |  | VKIPVAIKESKANK | DRB1_14 | 5.38% |
|  |  |  | VAIKESKANKEILD | DRB1_01 | 2.02% |
|  |  |  | KIPVAIKESKANKE | DRB1_01 | 0.00% |
|  |  |  | KIPVAIKESKANKE | DRB1_08 | 0.00% |
|  |  |  | VAIKESKANKEILD | DRB1_08 | 0.00% |
|  |  |  | VAIKESKANKEILD | DRB1_09 | 0.00% |
|  |  |  | KIPVAIKESKANKE | DRB1_11 | 2.57% |
|  |  |  | VAIKESKANKEILD | DRB1_11 | 0.00% |
|  |  |  | KIPVAIKESKANKE | DRB1_12 | 0.00% |
|  |  |  | VAIKESKANKEILD | DRB1_12 | 0.00% |
|  |  |  | VAIKESKANKEILD | DRB1_13 | 0.00% |
|  |  |  | VAIKESKANKEILD | DRB1_131 | 0.00% |
|  |  |  | KIPVAIKESKANKE | DRB1_13 | 0.00% |
|  |  |  | KIPVAIKESKANKE | DRB1_14 | 5.38% |
|  |  |  | VAIKESKANKEILD | DRB1_14 | 5.38% |
|  |  |  | PVAIKESKANKEI | DRB1_01 | 0.00% |
|  |  |  | PVAIKESKANKEI | DRB1_08 | 0.00% |
|  |  |  | PVAIKESKANKEI | DRB1_11 | 0.00% |
|  |  |  | PVAIKESKANKEI | DRB1_12 | 0.00% |
|  |  |  | PVAIKESKANKEI | DRB1_13 | 0.00% |
|  |  |  | PVAIKESKANKEI | DRB1_14 | 5.38% |
|  |  |  | IPVAIKESKANKE | DRB1_01 | 0.00% |
|  |  |  | KIPVAIKESKANK | DRB1_08 | 0.00% |
|  |  |  | IPVAIKESKANKE | DRB1_08 | 0.00% |
|  |  |  | KIPVAIKESKANK | DRB1_11 | 0.00% |
|  |  |  | IPVAIKESKANKE | DRB1_11 | 0.00% |
|  |  |  | KIPVAIKESKANK | DRB1_12 | 0.00% |
|  |  |  | IPVAIKESKANKE | DRB1_12 | 0.00% |
|  |  |  | KIPVAIKESKANK | DRB1_13 | 0.00% |
|  |  |  | IPVAIKESKANKE | DRB1_13 | 0.00% |
|  |  |  | KIPVAIKESKANK | DRB1_14 | 5.38% |
|  |  |  | IPVAIKESKANKE | DRB1_14 | 5.38% |
|  |  |  | VAIKESKANKEI | DRB1_01 | 0.00% |
|  |  |  | VAIKESKANKEI | DRB1_08 | 0.00% |
|  |  |  | VAIKESKANKEI | DRB1_11 | 0.00% |
|  |  |  | VAIKESKANKEI | DRB1_13 | 0.00% |
|  |  |  | VAIKESKANKEI | DRB1_14 | 5.38% |
|  |  |  | VKIPVAIKESKAN | DRB1_08 | 0.00% |
|  |  |  | VKIPVAIKESKAN | DRB1_11 | 0.00% |
|  |  |  | VKIPVAIKESKAN | DRB1_12 | 0.00% |
|  |  |  | VKIPVAIKESKAN | DRB1_13 | 0.00% |
|  |  |  | VKIPVAIKESKAN | DRB1_14 | 5.38% |
|  |  |  | AIKESKANKEIL | DRB1_01 | 0.00% |
|  |  |  | AIKESKANKEIL | DRB1_08 | 0.00% |
|  |  |  | AIKESKANKEIL | DRB1_11 | 0.00% |
|  |  |  | AIKESKANKEIL | DRB1_13 | 0.00% |
|  |  |  | AIKESKANKEIL | DRB1_14 | 1.67% |
|  |  |  | AIKESKANKEILD | DRB1_01 | 0.00% |
|  |  |  | AIKESKANKEILDE | DRB1_01 | 0.00% |
|  |  |  | IPVAIKESKANK | DRB1_08 | 0.00% |
|  |  |  | AIKESKANKEILD | DRB1_08 | 0.00% |
|  |  |  | AIKESKANKEILDE | DRB1_08 | 0.00% |
|  |  |  | IPVAIKESKANK | DRB1_11 | 0.00% |
|  |  |  | AIKESKANKEILD | DRB1_11 | 0.00% |
|  |  |  | AIKESKANKEILDE | DRB1_11 | 0.00% |
|  |  |  | IPVAIKESKANK | DRB1_12 | 0.00% |
|  |  |  | IPVAIKESKANK | DRB1_13 | 0.00% |
|  |  |  | AIKESKANKEILD | DRB1_13 | 0.00% |
|  |  |  | AIKESKANKEILDE | DRB1_13 | 0.00% |
|  |  |  | AIKESKANKEILD | DRB1_14 | 1.67% |
|  |  |  | AIKESKANKEILDE | DRB1_14 | 1.67% |
|  |  |  | IPVAIKESKANK | DRB1_14 | 0.00% |
|  |  |  | VKIPVAIKESKA | DRB1_08 | 0.00% |
|  |  |  | VKIPVAIKESKA | DRB1_11 | 0.00% |
|  |  |  | VKIPVAIKESKA | DRB1_12 | 0.00% |
|  |  |  | VKIPVAIKESKA | DRB1_13 | 0.00% |
|  |  |  | VKIPVAIKESKA | DRB1_14 | 0.00% |
|  |  |  | KIPVAIKESKAN | DRB1_08 | 0.00% |
|  |  |  | KIPVAIKESKAN | DRB1_11 | 0.00% |
|  |  |  | KIPVAIKESKAN | DRB1_12 | 0.00% |
|  |  |  | KIPVAIKESKAN | DRB1_13 | 0.00% |
|  |  |  | KIPVAIKESKAN | DRB1_14 | 0.00% |
|  |  |  | PVAIKESKANKE | DRB1_08 | 0.00% |
|  |  |  | PVAIKESKANKE | DRB1_11 | 0.00% |
|  |  |  | PVAIKESKANKE | DRB1_13 | 0.00% |
|  |  |  | PVAIKESKANKE | DRB1_14 | 0.00% |
|  |  |  | VAIKESKANKE | DRB1_08 | 0.00% |
|  |  |  | AIKESKANKEI | DRB1_08 | 0.00% |
|  |  |  | VAIKESKANKE | DRB1_13 | 0.00% |
|  |  |  | AIKESKANKEI | DRB1_13 | 0.00% |
|  |  |  | VKIPVAIKESK | DRB1_08 | 0.00% |
|  |  |  | KIPVAIKESKA | DRB1_08 | 0.00% |
|  |  |  | VKIPVAIKESK | DRB1_13 | 0.00% |
|  |  |  | KIPVAIKESKA | DRB1_13 | 0.00% |
|  |  |  | IPVAIKESKAN | DRB1_08 | 0.00% |
|  |  |  | PVAIKESKANK | DRB1_08 | 0.00% |
|  |  |  | SKANKEILDEAYVM | DRB1_01 | 0.00% |
|  |  |  | IPVAIKESKA | DRB1_08 | 0.00% |
| Total |  | 53.92% |  |  | 30.46% |
