# Supplementary material for: Immunogenicity of Del19 EGFR mutations in Chinese patients affected by lung adenocarcinoma
Source: BMC Immunol. 2019 Nov 13;20:43. doi: 10.1186/s12865-019-0320-1 (PMC6854806; doi:10.1186/s12865-019-0320-1)
Supplement: Supplementary file 3 — Additional file 3. Predicted HLA binding epitopes for EGFR delL747_T751. [file 12865_2019_320_MOESM3_ESM.doc]

**Supplemental Table 3, Predicted HLA binding epitopes for EGFR delL747_T751** **by Chinese NSCLC patients as predicted by NetMHC4.0.** The percentages are the total frequencies of HLA alleles which may present a mutant EGFR.

| Class I | | | Class II | | |
| --- | --- | --- | --- | --- | --- |
| Neopeptide | HLA alleles | Frequency | Neopeptide | HLA alleles | Frequency |
| AIKESPKANK | HLA-A*03 | 2.88% | GEKVKIPVAIKESP | DRB1_14 | 12.37% |
| AIKESPKANK | HLA-A*11 | 0.66% | GEKVKIPVAIKESP | DRB1_13 | 0.00% |
| AIKESPKANK | HLA-A*30 | 7.56% | GEKVKIPVAIKESP | DRB1_12 | 1.90% |
| IPVAIKESPK | HLA-A*03 | 0.00% | GEKVKIPVAIKESP | DRB1_11 | 2.57% |
| IPVAIKESPK | HLA-A*11 | 0.46% | GEKVKIPVAIKESP | DRB1_08 | 3.69% |
| IPVAIKESPK | HLA-A*68 | 0.10% | GEKVKIPVAIKESP | DRB1_01 | 2.02% |
| KIPVAIKESPK | HLA-A*03 | 0.00% | KIPVAIKESPKANK | DRB1_14 | 5.38% |
| KIPVAIKESPK | HLA-A*11 | 0.46% | KIPVAIKESPKANK | DRB1_13 | 0.00% |
|  |  |  | KIPVAIKESPKANK | DRB1_12 | 1.90% |
|  |  |  | KIPVAIKESPKANK | DRB1_11 | 2.57% |
|  |  |  | KIPVAIKESPKANK | DRB1_08 | 4.92% |
|  |  |  | KIPVAIKESPKANK | DRB1_01 | 2.02% |
|  |  |  | KVKIPVAIKESPKA | DRB1_14 | 5.38% |
|  |  |  | KVKIPVAIKESPKA | DRB1_13 | 0.00% |
|  |  |  | KVKIPVAIKESPKA | DRB1_12 | 1.90% |
|  |  |  | KVKIPVAIKESPKA | DRB1_11 | 2.57% |
|  |  |  | KVKIPVAIKESPKA | DRB1_08 | 4.92% |
|  |  |  | KVKIPVAIKESPKA | DRB1_01 | 0.00% |
|  |  |  | EKVKIPVAIKESPK | DRB1_14 | 5.38% |
|  |  |  | EKVKIPVAIKESPK | DRB1_13 | 0.00% |
|  |  |  | EKVKIPVAIKESPK | DRB1_12 | 1.90% |
|  |  |  | EKVKIPVAIKESPK | DRB1_11 | 2.57% |
|  |  |  | EKVKIPVAIKESPK | DRB1_08 | 3.69% |
|  |  |  | EKVKIPVAIKESPK | DRB1_01 | 2.02% |
|  |  |  | EKVKIPVAIKESP | DRB1_14 | 5.38% |
|  |  |  | EKVKIPVAIKESP | DRB1_13 | 0.00% |
|  |  |  | EKVKIPVAIKESP | DRB1_12 | 1.90% |
|  |  |  | EKVKIPVAIKESP | DRB1_11 | 2.57% |
|  |  |  | EKVKIPVAIKESP | DRB1_08 | 3.69% |
|  |  |  | EKVKIPVAIKESP | DRB1_01 | 2.02% |
|  |  |  | IPVAIKESPKANKE | DRB1_14 | 5.38% |
|  |  |  | IPVAIKESPKANKE | DRB1_13 | 0.00% |
|  |  |  | IPVAIKESPKANKE | DRB1_12 | 0.00% |
|  |  |  | IPVAIKESPKANKE | DRB1_11 | 2.57% |
|  |  |  | IPVAIKESPKANKE | DRB1_08 | 4.92% |
|  |  |  | IPVAIKESPKANKE | DRB1_01 | 2.02% |
|  |  |  | IPVAIKESPKANK | DRB1_14 | 5.38% |
|  |  |  | PVAIKESPKANKEI | DRB1_14 | 5.38% |
|  |  |  | IPVAIKESPKANK | DRB1_13 | 0.00% |
|  |  |  | PVAIKESPKANKEI | DRB1_13 | 0.00% |
|  |  |  | IPVAIKESPKANK | DRB1_12 | 0.00% |
|  |  |  | PVAIKESPKANKEI | DRB1_12 | 0.00% |
|  |  |  | IPVAIKESPKANK | DRB1_11 | 2.57% |
|  |  |  | PVAIKESPKANKEI | DRB1_11 | 2.57% |
|  |  |  | IPVAIKESPKANK | DRB1_08 | 3.52% |
|  |  |  | PVAIKESPKANKEI | DRB1_08 | 3.52% |
|  |  |  | IPVAIKESPKANK | DRB1_01 | 2.02% |
|  |  |  | PVAIKESPKANKEI | DRB1_01 | 0.00% |
|  |  |  | VKIPVAIKESPKAN | DRB1_14 | 5.38% |
|  |  |  | VKIPVAIKESPKAN | DRB1_13 | 0.00% |
|  |  |  | VKIPVAIKESPKAN | DRB1_12 | 0.00% |
|  |  |  | VKIPVAIKESPKAN | DRB1_11 | 2.57% |
|  |  |  | VKIPVAIKESPKAN | DRB1_08 | 2.29% |
|  |  |  | VKIPVAIKESPKAN | DRB1_01 | 2.02% |
|  |  |  | KVKIPVAIKESPK | DRB1_14 | 5.38% |
|  |  |  | KIPVAIKESPKAN | DRB1_14 | 5.38% |
|  |  |  | KVKIPVAIKESPK | DRB1_13 | 0.00% |
|  |  |  | KIPVAIKESPKAN | DRB1_13 | 0.00% |
|  |  |  | KVKIPVAIKESPK | DRB1_12 | 0.00% |
|  |  |  | KIPVAIKESPKAN | DRB1_12 | 0.00% |
|  |  |  | KVKIPVAIKESPK | DRB1_11 | 2.57% |
|  |  |  | KIPVAIKESPKAN | DRB1_11 | 2.57% |
|  |  |  | KVKIPVAIKESPK | DRB1_08 | 0.00% |
|  |  |  | KIPVAIKESPKAN | DRB1_08 | 0.00% |
|  |  |  | KIPVAIKESPKAN | DRB1_01 | 2.02% |
|  |  |  | VAIKESPKANKEIL | DRB1_14 | 5.38% |
|  |  |  | VAIKESPKANKEIL | DRB1_13 | 0.00% |
|  |  |  | VAIKESPKANKEIL | DRB1_12 | 0.00% |
|  |  |  | VAIKESPKANKEIL | DRB1_11 | 2.57% |
|  |  |  | VAIKESPKANKEIL | DRB1_08 | 0.00% |
|  |  |  | PVAIKESPKANKE | DRB1_14 | 5.38% |
|  |  |  | PVAIKESPKANKE | DRB1_13 | 0.00% |
|  |  |  | PVAIKESPKANKE | DRB1_12 | 0.00% |
|  |  |  | PVAIKESPKANKE | DRB1_11 | 2.57% |
|  |  |  | PVAIKESPKANKE | DRB1_08 | 0.00% |
|  |  |  | PVAIKESPKANKE | DRB1_01 | 0.00% |
|  |  |  | VAIKESPKANKEI | DRB1_14 | 5.38% |
|  |  |  | VAIKESPKANKEI | DRB1_13 | 0.00% |
|  |  |  | VAIKESPKANKEI | DRB1_12 | 0.00% |
|  |  |  | VAIKESPKANKEI | DRB1_11 | 2.57% |
|  |  |  | VAIKESPKANKEI | DRB1_08 | 0.00% |
|  |  |  | KVKIPVAIKESP | DRB1_14 | 5.38% |
|  |  |  | KVKIPVAIKESP | DRB1_13 | 0.00% |
|  |  |  | KVKIPVAIKESP | DRB1_12 | 0.00% |
|  |  |  | KVKIPVAIKESP | DRB1_11 | 2.57% |
|  |  |  | KVKIPVAIKESP | DRB1_08 | 0.00% |
|  |  |  | PVAIKESPKANK | DRB1_14 | 5.38% |
|  |  |  | PVAIKESPKANK | DRB1_13 | 0.00% |
|  |  |  | PVAIKESPKANK | DRB1_12 | 0.00% |
|  |  |  | PVAIKESPKANK | DRB1_11 | 0.00% |
|  |  |  | PVAIKESPKANK | DRB1_08 | 0.00% |
|  |  |  | PVAIKESPKANK | DRB1_01 | 0.00% |
|  |  |  | VKIPVAIKESPKA | DRB1_14 | 1.67% |
|  |  |  | VKIPVAIKESPKA | DRB1_13 | 0.00% |
|  |  |  | VKIPVAIKESPKA | DRB1_12 | 0.00% |
|  |  |  | VKIPVAIKESPKA | DRB1_11 | 0.00% |
|  |  |  | VKIPVAIKESPKA | DRB1_08 | 0.00% |
|  |  |  | VAIKESPKANKE | DRB1_14 | 3.71% |
|  |  |  | IPVAIKESPKAN | DRB1_14 | 0.00% |
|  |  |  | IPVAIKESPKAN | DRB1_13 | 0.00% |
|  |  |  | VAIKESPKANKE | DRB1_13 | 0.00% |
|  |  |  | IPVAIKESPKAN | DRB1_12 | 0.00% |
|  |  |  | IPVAIKESPKAN | DRB1_11 | 0.00% |
|  |  |  | VAIKESPKANKE | DRB1_11 | 0.00% |
|  |  |  | IPVAIKESPKAN | DRB1_08 | 0.00% |
|  |  |  | VAIKESPKANKE | DRB1_08 | 0.00% |
|  |  |  | IPVAIKESPKAN | DRB1_01 | 0.00% |
|  |  |  | KIPVAIKESPKA | DRB1_14 | 0.00% |
|  |  |  | KIPVAIKESPKA | DRB1_13 | 0.00% |
|  |  |  | KIPVAIKESPKA | DRB1_12 | 0.00% |
|  |  |  | KIPVAIKESPKA | DRB1_11 | 0.00% |
|  |  |  | KIPVAIKESPKA | DRB1_08 | 0.00% |
|  |  |  | AIKESPKANKEIL | DRB1_14 | 3.71% |
|  |  |  | AIKESPKANKEILD | DRB1_14 | 3.71% |
|  |  |  | AIKESPKANKEILD | DRB1_13 | 0.00% |
|  |  |  | AIKESPKANKEIL | DRB1_13 | 0.00% |
|  |  |  | AIKESPKANKEIL | DRB1_11 | 0.00% |
|  |  |  | AIKESPKANKEILD | DRB1_11 | 0.00% |
|  |  |  | AIKESPKANKEIL | DRB1_08 | 0.00% |
|  |  |  | AIKESPKANKEILD | DRB1_08 | 0.00% |
|  |  |  | PVAIKESPKAN | DRB1_14 | 0.00% |
|  |  |  | VAIKESPKANK | DRB1_14 | 0.00% |
|  |  |  | PVAIKESPKAN | DRB1_13 | 0.00% |
|  |  |  | VAIKESPKANK | DRB1_13 | 0.00% |
|  |  |  | PVAIKESPKAN | DRB1_08 | 0.00% |
|  |  |  | VAIKESPKANK | DRB1_08 | 0.00% |
|  |  |  | IPVAIKESPKA | DRB1_14 | 0.00% |
|  |  |  | IPVAIKESPKA | DRB1_13 | 0.00% |
|  |  |  | IPVAIKESPKA | DRB1_08 | 0.00% |
|  |  |  | AIKESPKANKEI | DRB1_14 | 0.00% |
|  |  |  | AIKESPKANKEI | DRB1_13 | 0.00% |
|  |  |  | AIKESPKANKEI | DRB1_08 | 0.00% |
|  |  |  | VKIPVAIKESPK | DRB1_13 | 0.00% |
|  |  |  | VKIPVAIKESPK | DRB1_08 | 0.00% |
|  |  |  | PVAIKESPKA | DRB1_08 | 0.00% |
| Total |  | 11.20% |  |  | 23.78% |
