# Supplementary material for: Immunogenicity of Del19 EGFR mutations in Chinese patients affected by lung adenocarcinoma
Source: BMC Immunol. 2019 Nov 13;20:43. doi: 10.1186/s12865-019-0320-1 (PMC6854806; doi:10.1186/s12865-019-0320-1)
Supplement: Supplementary file 4 — Additional file 4. Predicted HLA binding epitopes for EGFR delL747_A750insP. [file 12865_2019_320_MOESM4_ESM.doc]

**Supplemental Table 4, Predicted HLA binding epitopes for EGFR delL747_A750insP by Chinese NSCLC patients as predicted by NetMHC4.0.** The percentages are the total frequencies of HLA alleles which may present a mutant EGFR.

| Class I | | | Class II | | |
| --- | --- | --- | --- | --- | --- |
| Neopeptide | HLA alleles | Frequency | Neopeptide | HLA alleles | Frequency |
| VAIKEPTSPK | HLA-A*68 | 0.85% | GEKVKIPVAIKEPT | DRB1_01 | 2.02% |
| VAIKEPTSPK | HLA-A*34 | 0.00% | GEKVKIPVAIKEPT | DRB1_08 | 3.69% |
| VAIKEPTSPK | HLA-A*30 | 7.56% | GEKVKIPVAIKEPT | DRB1_11 | 2.57% |
| VAIKEPTSPK | HLA-A*11 | 26.45% | GEKVKIPVAIKEPT | DRB1_12 | 1.90% |
| VAIKEPTSPK | HLA-A*03 | 2.88% | GEKVKIPVAIKEPT | DRB1_13 | 0.00% |
| AIKEPTSPK | HLA-A*68 | 0.10% | GEKVKIPVAIKEPT | DRB1_14 | 5.38% |
| AIKEPTSPK | HLA-A*34 | 0.00% | EKVKIPVAIKEPTS | DRB1_01 | 2.02% |
| AIKEPTSPK | HLA-A*31 | 0.32% | EKVKIPVAIKEPTS | DRB1_08 | 4.92% |
| AIKEPTSPK | HLA-A*30 | 7.56% | EKVKIPVAIKEPTS | DRB1_11 | 2.57% |
| AIKEPTSPK | HLA-A*11 | 26.71% | EKVKIPVAIKEPTS | DRB1_12 | 1.90% |
| AIKEPTSPK | HLA-A*03 | 3.64% | EKVKIPVAIKEPTS | DRB1_13 | 0.00% |
| IPVAIKEPT | HLA-B*56 | 0.00% | EKVKIPVAIKEPTS | DRB1_14 | 5.38% |
| IPVAIKEPT | HLA-B*55 | 3.04% | EKVKIPVAIKEPT | DRB1_01 | 2.02% |
| IPVAIKEPT | HLA-B*54 | 3.16% | EKVKIPVAIKEPT | DRB1_08 | 3.69% |
| IPVAIKEPT | HLA-B*39 | 0.00% | EKVKIPVAIKEPT | DRB1_11 | 2.57% |
| IPVAIKEPT | HLA-B*07 | 0.00% | EKVKIPVAIKEPT | DRB1_12 | 1.90% |
| AIKEPTSPKA | HLA-A*30 | 7.56% | EKVKIPVAIKEPT | DRB1_13 | 0.00% |
| AIKEPTSPKA | HLA-A*11 | 0.46% | EKVKIPVAIKEPT | DRB1_14 | 5.38% |
| AIKEPTSPKA | HLA-A*03 | 0.00% | KVKIPVAIKEPTS | DRB1_08 | 2.29% |
| PVAIKEPTSPK | HLA-A*03 | 0.00% | KVKIPVAIKEPTSP | DRB1_08 | 2.29% |
|  |  |  | KVKIPVAIKEPTS | DRB1_11 | 2.57% |
|  |  |  | KVKIPVAIKEPTSP | DRB1_11 | 2.57% |
|  |  |  | KVKIPVAIKEPTS | DRB1_12 | 0.00% |
|  |  |  | KVKIPVAIKEPTSP | DRB1_12 | 0.00% |
|  |  |  | KVKIPVAIKEPTS | DRB1_13 | 0.00% |
|  |  |  | KVKIPVAIKEPTSP | DRB1_13 | 0.00% |
|  |  |  | KVKIPVAIKEPTS | DRB1_14 | 5.38% |
|  |  |  | KVKIPVAIKEPTSP | DRB1_14 | 5.38% |
|  |  |  | KVKIPVAIKEPT | DRB1_08 | 0.00% |
|  |  |  | KVKIPVAIKEPT | DRB1_11 | 2.57% |
|  |  |  | KVKIPVAIKEPT | DRB1_12 | 0.00% |
|  |  |  | KVKIPVAIKEPT | DRB1_13 | 0.00% |
|  |  |  | KVKIPVAIKEPT | DRB1_14 | 5.38% |
|  |  |  | KIPVAIKEPTSPKA | DRB1_08 | 0.00% |
|  |  |  | KIPVAIKEPTSPKA | DRB1_12 | 0.00% |
|  |  |  | KIPVAIKEPTSPKA | DRB1_13 | 0.00% |
|  |  |  | KIPVAIKEPTSPKA | DRB1_14 | 0.00% |
|  |  |  | VKIPVAIKEPTSPK | DRB1_08 | 0.00% |
|  |  |  | IPVAIKEPTSPKAN | DRB1_08 | 0.00% |
|  |  |  | VKIPVAIKEPTSPK | DRB1_12 | 0.00% |
|  |  |  | VKIPVAIKEPTSPK | DRB1_13 | 0.00% |
|  |  |  | IPVAIKEPTSPKAN | DRB1_13 | 0.00% |
|  |  |  | VKIPVAIKEPTSPK | DRB1_14 | 0.00% |
|  |  |  | IPVAIKEPTSPKAN | DRB1_14 | 0.00% |
|  |  |  | IPVAIKEPTSPKA | DRB1_08 | 0.00% |
|  |  |  | PVAIKEPTSPKANK | DRB1_08 | 0.00% |
|  |  |  | IPVAIKEPTSPKA | DRB1_13 | 0.00% |
|  |  |  | PVAIKEPTSPKANK | DRB1_13 | 0.00% |
|  |  |  | VKIPVAIKEPTSP | DRB1_08 | 0.00% |
|  |  |  | KIPVAIKEPTSPK | DRB1_08 | 0.00% |
|  |  |  | VKIPVAIKEPTSP | DRB1_13 | 0.00% |
|  |  |  | KIPVAIKEPTSPK | DRB1_13 | 0.00% |
|  |  |  | VKIPVAIKEPTS | DRB1_08 | 0.00% |
|  |  |  | VKIPVAIKEPTS | DRB1_13 | 0.00% |
|  |  |  | PVAIKEPTSPKAN | DRB1_08 | 0.00% |
|  |  |  | VKIPVAIKEPT | DRB1_08 | 0.00% |
|  |  |  | KIPVAIKEPTSP | DRB1_08 | 0.00% |
|  |  |  | KIPVAIKEPTS | DRB1_08 | 0.00% |
| Total |  | 45.28% |  |  | 16.79% |
