# Supplementary material for: Immunogenicity of Del19 EGFR mutations in Chinese patients affected by lung adenocarcinoma
Source: BMC Immunol. 2019 Nov 13;20:43. doi: 10.1186/s12865-019-0320-1 (PMC6854806; doi:10.1186/s12865-019-0320-1)
Supplement: Supplementary file 5 — Additional file 5. Predicted HLA binding epitopes for EGFR delL747_S752. [file 12865_2019_320_MOESM5_ESM.doc]

**Supplemental Table 5, Predicted HLA binding epitopes for EGFR delL747_S752 by Chinese NSCLC patients as predicted by NetMHC4.0.** The percentages are the total frequencies of HLA alleles which may present a mutant EGFR.

| Class I | | | Class II | | |
| --- | --- | --- | --- | --- | --- |
| Neopeptide | HLA alleles | Frequency | Neopeptide | HLA alleles | Frequency |
| AIKEPKANK | HLA-A*68 | 0.10% | EKVKIPVAIKEPKA | DRB1_01 | 2.02% |
| AIKEPKANK | HLA-A*34 | 0.00% | EKVKIPVAIKEPKA | DRB1_08 | 4.92% |
| AIKEPKANK | HLA-A*31 | 0.32% | EKVKIPVAIKEPKA | DRB1_11 | 2.57% |
| AIKEPKANK | HLA-A*30 | 7.56% | EKVKIPVAIKEPKA | DRB1_12 | 1.90% |
| AIKEPKANK | HLA-A*11 | 24.34% | EKVKIPVAIKEPKA | DRB1_13 | 0.00% |
| AIKEPKANK | HLA-A*03 | 2.88% | EKVKIPVAIKEPKA | DRB1_14 | 12.37% |
| KIPVAIKEPK | HLA-A*68 | 0.10% | GEKVKIPVAIKEPK | DRB1_01 | 2.02% |
| KIPVAIKEPK | HLA-A*31 | 0.32% | GEKVKIPVAIKEPK | DRB1_08 | 4.92% |
| KIPVAIKEPK | HLA-A*30 | 0.00% | GEKVKIPVAIKEPK | DRB1_11 | 2.57% |
| KIPVAIKEPK | HLA-A*11 | 25.75% | GEKVKIPVAIKEPK | DRB1_12 | 1.90% |
| KIPVAIKEPK | HLA-A*03 | 3.25% | GEKVKIPVAIKEPK | DRB1_13 | 0.00% |
| IPVAIKEPKA | HLA-B*56 | 0.84% | GEKVKIPVAIKEPK | DRB1_14 | 12.37% |
| IPVAIKEPKA | HLA-B*55 | 3.15% | EKVKIPVAIKEPK | DRB1_01 | 2.02% |
| IPVAIKEPKA | HLA-B*54 | 3.16% | EKVKIPVAIKEPK | DRB1_08 | 4.92% |
| VAIKEPKANK | HLA-A*68 | 0.10% | EKVKIPVAIKEPK | DRB1_11 | 2.57% |
| VAIKEPKANK | HLA-A*34 | 0.00% | EKVKIPVAIKEPK | DRB1_12 | 1.90% |
| VAIKEPKANK | HLA-A*11 | 0.46% | EKVKIPVAIKEPK | DRB1_13 | 0.00% |
| VAIKEPKANK | HLA-A*03 | 0.00% | EKVKIPVAIKEPK | DRB1_14 | 12.37% |
| IPVAIKEPK | HLA-B*55 | 0.00% | KVKIPVAIKEPKAN | DRB1_01 | 2.02% |
| IPVAIKEPK | HLA-A*68 | 0.00% | KVKIPVAIKEPKAN | DRB1_08 | 4.92% |
|  |  |  | KVKIPVAIKEPKAN | DRB1_11 | 2.57% |
|  |  |  | KVKIPVAIKEPKAN | DRB1_12 | 1.90% |
|  |  |  | KVKIPVAIKEPKAN | DRB1_13 | 0.00% |
|  |  |  | KVKIPVAIKEPKAN | DRB1_14 | 5.38% |
|  |  |  | KVKIPVAIKEPKA | DRB1_01 | 0.00% |
|  |  |  | KVKIPVAIKEPKA | DRB1_08 | 4.92% |
|  |  |  | KVKIPVAIKEPKA | DRB1_11 | 2.57% |
|  |  |  | KVKIPVAIKEPKA | DRB1_12 | 1.90% |
|  |  |  | KVKIPVAIKEPKA | DRB1_13 | 0.00% |
|  |  |  | KVKIPVAIKEPKA | DRB1_14 | 5.38% |
|  |  |  | KVKIPVAIKEPK | DRB1_08 | 3.69% |
|  |  |  | KVKIPVAIKEPK | DRB1_11 | 2.57% |
|  |  |  | KVKIPVAIKEPK | DRB1_12 | 0.00% |
|  |  |  | KVKIPVAIKEPK | DRB1_13 | 0.00% |
|  |  |  | KVKIPVAIKEPK | DRB1_14 | 5.38% |
|  |  |  | VKIPVAIKEPKANK | DRB1_01 | 0.00% |
|  |  |  | VKIPVAIKEPKANK | DRB1_08 | 0.00% |
|  |  |  | VKIPVAIKEPKANK | DRB1_11 | 2.57% |
|  |  |  | VKIPVAIKEPKANK | DRB1_12 | 0.00% |
|  |  |  | VKIPVAIKEPKANK | DRB1_13 | 0.00% |
|  |  |  | VKIPVAIKEPKANK | DRB1_14 | 5.38% |
|  |  |  | KIPVAIKEPKANKE | DRB1_08 | 0.00% |
|  |  |  | KIPVAIKEPKANKE | DRB1_11 | 2.57% |
|  |  |  | KIPVAIKEPKANKE | DRB1_12 | 0.00% |
|  |  |  | KIPVAIKEPKANKE | DRB1_13 | 0.00% |
|  |  |  | KIPVAIKEPKANKE | DRB1_14 | 5.38% |
|  |  |  | KIPVAIKEPKANK | DRB1_08 | 0.00% |
|  |  |  | KIPVAIKEPKANK | DRB1_11 | 2.57% |
|  |  |  | KIPVAIKEPKANK | DRB1_12 | 0.00% |
|  |  |  | KIPVAIKEPKANK | DRB1_13 | 0.00% |
|  |  |  | KIPVAIKEPKANK | DRB1_14 | 5.38% |
|  |  |  | IPVAIKEPKANKEI | DRB1_08 | 0.00% |
|  |  |  | IPVAIKEPKANKEI | DRB1_11 | 2.57% |
|  |  |  | IPVAIKEPKANKEI | DRB1_12 | 0.00% |
|  |  |  | IPVAIKEPKANKEI | DRB1_13 | 0.00% |
|  |  |  | IPVAIKEPKANKEI | DRB1_14 | 5.38% |
|  |  |  | VKIPVAIKEPKAN | DRB1_08 | 0.00% |
|  |  |  | VKIPVAIKEPKAN | DRB1_11 | 0.00% |
|  |  |  | VKIPVAIKEPKAN | DRB1_12 | 0.00% |
|  |  |  | VKIPVAIKEPKAN | DRB1_13 | 0.00% |
|  |  |  | VKIPVAIKEPKAN | DRB1_14 | 5.38% |
|  |  |  | VKIPVAIKEPKA | DRB1_08 | 0.00% |
|  |  |  | KIPVAIKEPKAN | DRB1_08 | 0.00% |
|  |  |  | IPVAIKEPKANKE | DRB1_08 | 0.00% |
|  |  |  | VKIPVAIKEPKA | DRB1_11 | 0.00% |
|  |  |  | KIPVAIKEPKAN | DRB1_11 | 0.00% |
|  |  |  | IPVAIKEPKANKE | DRB1_11 | 0.00% |
|  |  |  | VKIPVAIKEPKA | DRB1_12 | 0.00% |
|  |  |  | KIPVAIKEPKAN | DRB1_12 | 0.00% |
|  |  |  | IPVAIKEPKANKE | DRB1_12 | 0.00% |
|  |  |  | VKIPVAIKEPKA | DRB1_13 | 0.00% |
|  |  |  | KIPVAIKEPKAN | DRB1_13 | 0.00% |
|  |  |  | IPVAIKEPKANKE | DRB1_13 | 0.00% |
|  |  |  | VKIPVAIKEPKA | DRB1_14 | 0.00% |
|  |  |  | KIPVAIKEPKAN | DRB1_14 | 0.00% |
|  |  |  | IPVAIKEPKANKE | DRB1_14 | 0.00% |
|  |  |  | IPVAIKEPKANK | DRB1_08 | 0.00% |
|  |  |  | IPVAIKEPKANK | DRB1_11 | 0.00% |
|  |  |  | IPVAIKEPKANK | DRB1_12 | 0.00% |
|  |  |  | IPVAIKEPKANK | DRB1_13 | 0.00% |
|  |  |  | IPVAIKEPKANK | DRB1_14 | 0.00% |
|  |  |  | PVAIKEPKANKEIL | DRB1_08 | 0.00% |
|  |  |  | PVAIKEPKANKEIL | DRB1_11 | 0.00% |
|  |  |  | PVAIKEPKANKEIL | DRB1_13 | 0.00% |
|  |  |  | PVAIKEPKANKEIL | DRB1_14 | 0.00% |
|  |  |  | PVAIKEPKANKEI | DRB1_08 | 0.00% |
|  |  |  | PVAIKEPKANKEI | DRB1_13 | 0.00% |
|  |  |  | PVAIKEPKANKEI | DRB1_14 | 0.00% |
|  |  |  | VAIKEPKANKEIL | DRB1_08 | 0.00% |
|  |  |  | VAIKEPKANKEILD | DRB1_08 | 0.00% |
|  |  |  | VAIKEPKANKEIL | DRB1_13 | 0.00% |
|  |  |  | VAIKEPKANKEILD | DRB1_13 | 0.00% |
|  |  |  | KIPVAIKEPKA | DRB1_08 | 0.00% |
|  |  |  | KIPVAIKEPKA | DRB1_13 | 0.00% |
|  |  |  | KIPVAIKEPKA | DRB1_14 | 0.00% |
|  |  |  | VKIPVAIKEPK | DRB1_08 | 0.00% |
|  |  |  | IPVAIKEPKAN | DRB1_08 | 0.00% |
|  |  |  | PVAIKEPKANK | DRB1_08 | 0.00% |
|  |  |  | PVAIKEPKANKE | DRB1_08 | 0.00% |
|  |  |  | VAIKEPKANKEI | DRB1_08 | 0.00% |
|  |  |  | IPVAIKEPKA | DRB1_08 | 0.00% |
| Total |  | 44.13% |  |  | 23.78% |
