# Supplementary material for: Immunogenicity of Del19 EGFR mutations in Chinese patients affected by lung adenocarcinoma
Source: BMC Immunol. 2019 Nov 13;20:43. doi: 10.1186/s12865-019-0320-1 (PMC6854806; doi:10.1186/s12865-019-0320-1)
Supplement: Supplementary file 6 — Additional file 6. Predicted HLA binding epitopes for EGFR delE746_S752insV. [file 12865_2019_320_MOESM6_ESM.doc]

**Supplemental Table 6, Predicted HLA binding epitopes for EGFR delE746_S752insV by Chinese NSCLC patients as predicted by NetMHC4.0.** The percentages are the total frequencies of HLA alleles which may present a mutant EGFR.

| Class I | | | Class II | | |
| --- | --- | --- | --- | --- | --- |
| Neopeptide | HLA alleles | Frequency | Neopeptide | HLA alleles | Frequency |
| KIPVAIKVPK | HLA-A*03 | 3.64% | EKVKIPVAIKVPKA | DRB1_01 | 6.04% |
| KIPVAIKVPK | HLA-A*11 | 25.85% | EKVKIPVAIKVPKA | DRB1_07 | 8.50% |
| KIPVAIKVPK | HLA-A*30 | 7.56% | EKVKIPVAIKVPKA | DRB1_08 | 4.92% |
| KIPVAIKVPK | HLA-A*31 | 3.64% | EKVKIPVAIKVPKA | DRB1_09 | 0.00% |
| KIPVAIKVPK | HLA-A*33 | 0.00% | EKVKIPVAIKVPKA | DRB1_11 | 8.11% |
| KIPVAIKVPK | HLA-A*68 | 0.10% | EKVKIPVAIKVPKA | DRB1_12 | 1.90% |
| KIPVAIKVPK | HLA-A*74 | 0.00% | EKVKIPVAIKVPKA | DRB1_13 | 4.03% |
| AIKVPKANK | HLA-A*03 | 2.88% | EKVKIPVAIKVPKA | DRB1_14 | 13.50% |
| AIKVPKANK | HLA-A*11 | 0.66% | EKVKIPVAIKVPKA | DRB1_15 | 0.00% |
| AIKVPKANK | HLA-A*30 | 7.56% | EKVKIPVAIKVPKA | DRB1_16 | 6.81% |
| AIKVPKANK | HLA-A*31 | 3.64% | KVKIPVAIKVPKAN | DRB1_01 | 6.04% |
| AIKVPKANK | HLA-A*68 | 0.00% | KVKIPVAIKVPKAN | DRB1_07 | 0.00% |
| VPKANKEIL | HLA-B*07 | 3.51% | KVKIPVAIKVPKAN | DRB1_08 | 16.05% |
| VPKANKEIL | HLA-B*071 | 0.00% | KVKIPVAIKVPKAN | DRB1_09 | 0.00% |
| VPKANKEIL | HLA-B*072 | 0.00% | KVKIPVAIKVPKAN | DRB1_11 | 8.11% |
| VPKANKEIL | HLA-B*073 | 0.00% | KVKIPVAIKVPKAN | DRB1_12 | 1.90% |
| VPKANKEIL | HLA-B*074 | 0.00% | KVKIPVAIKVPKAN | DRB1_13 | 0.00% |
| VPKANKEIL | HLA-B*075 | 0.00% | KVKIPVAIKVPKAN | DRB1_14 | 13.50% |
| VPKANKEIL | HLA-B*077 | 0.00% | KVKIPVAIKVPKAN | DRB1_15 | 0.00% |
| VPKANKEIL | HLA-B*078 | 0.00% | KVKIPVAIKVPKAN | DRB1_16 | 6.81% |
| VPKANKEIL | HLA-B*079 | 0.00% | GEKVKIPVAIKVPK | DRB1_01 | 6.04% |
| VPKANKEIL | HLA-B*070 | 0.00% | GEKVKIPVAIKVPK | DRB1_07 | 8.50% |
| VPKANKEIL | HLA-B*08 | 0.00% | GEKVKIPVAIKVPK | DRB1_08 | 4.92% |
| VPKANKEIL | HLA-B*42 | 0.05% | GEKVKIPVAIKVPK | DRB1_09 | 0.00% |
| VPKANKEIL | HLA-B*55 | 0.18% | GEKVKIPVAIKVPK | DRB1_11 | 8.11% |
| IPVAIKVPKA | HLA-B*07 | 0.00% | GEKVKIPVAIKVPK | DRB1_12 | 1.90% |
| IPVAIKVPKA | HLA-B*42 | 0.00% | GEKVKIPVAIKVPK | DRB1_13 | 0.00% |
| IPVAIKVPKA | HLA-B*54 | 3.16% | GEKVKIPVAIKVPK | DRB1_14 | 13.50% |
| IPVAIKVPKA | HLA-B*55 | 3.44% | GEKVKIPVAIKVPK | DRB1_15 | 0.00% |
| IPVAIKVPKA | HLA-B*56 | 0.84% | GEKVKIPVAIKVPK | DRB1_16 | 6.81% |
| KVKIPVAIKV | HLA-A*02 | 9.17% | KVKIPVAIKVPKA | DRB1_01 | 6.04% |
| KVKIPVAIKV | HLA-A*025 | 0.00% | KVKIPVAIKVPKA | DRB1_07 | 0.00% |
| KVKIPVAIKV | HLA-A*026 | 0.00% | KVKIPVAIKVPKA | DRB1_08 | 4.92% |
| KVKIPVAIKV | HLA-A*027 | 0.00% | KVKIPVAIKVPKA | DRB1_09 | 0.00% |
| KVKIPVAIKV | HLA-A*028 | 0.00% | KVKIPVAIKVPKA | DRB1_11 | 8.11% |
| KVKIPVAIKV | HLA-A*022 | 0.00% | KVKIPVAIKVPKA | DRB1_12 | 1.90% |
| KVKIPVAIKV | HLA-A*024 | 0.00% | KVKIPVAIKVPKA | DRB1_13 | 0.00% |
| KVKIPVAIKV | HLA-A*029 | 0.00% | KVKIPVAIKVPKA | DRB1_14 | 13.50% |
| KVKIPVAIKV | HLA-A*020 | 0.00% | KVKIPVAIKVPKA | DRB1_15 | 0.00% |
| KVKIPVAIKV | HLA-A*023 | 0.00% | KVKIPVAIKVPKA | DRB1_16 | 6.81% |
| KVKIPVAIKV | HLA-A*03 | 0.00% | VKIPVAIKVPKANK | DRB1_01 | 6.04% |
| KVKIPVAIKV | HLA-A*30 | 7.56% | VKIPVAIKVPKANK | DRB1_08 | 16.05% |
| KVKIPVAIKV | HLA-B*27 | 0.00% | VKIPVAIKVPKANK | DRB1_09 | 0.00% |
| VAIKVPKANK | HLA-A*03 | 0.00% | VKIPVAIKVPKANK | DRB1_11 | 8.11% |
| VAIKVPKANK | HLA-A*11 | 24.14% | VKIPVAIKVPKANK | DRB1_12 | 1.90% |
| VAIKVPKANK | HLA-A*30 | 0.00% | VKIPVAIKVPKANK | DRB1_13 | 0.00% |
| VAIKVPKANK | HLA-A*34 | 0.00% | VKIPVAIKVPKANK | DRB1_14 | 13.50% |
| VAIKVPKANK | HLA-A*68 | 0.10% | VKIPVAIKVPKANK | DRB1_15 | 0.00% |
| IPVAIKVPK | HLA-A*68 | 0.00% | VKIPVAIKVPKANK | DRB1_16 | 6.81% |
| IPVAIKVPK | HLA-B*54 | 0.00% | EKVKIPVAIKVPK | DRB1_01 | 6.04% |
| IPVAIKVPK | HLA-B*55 | 0.00% | EKVKIPVAIKVPK | DRB1_07 | 0.00% |
| IPVAIKVPK | HLA-B*56 | 0.00% | EKVKIPVAIKVPK | DRB1_08 | 4.92% |
|  |  |  | EKVKIPVAIKVPK | DRB1_09 | 0.00% |
|  |  |  | EKVKIPVAIKVPK | DRB1_11 | 8.11% |
|  |  |  | EKVKIPVAIKVPK | DRB1_12 | 1.90% |
|  |  |  | EKVKIPVAIKVPK | DRB1_13 | 0.00% |
|  |  |  | EKVKIPVAIKVPK | DRB1_14 | 13.50% |
|  |  |  | EKVKIPVAIKVPK | DRB1_15 | 0.00% |
|  |  |  | EKVKIPVAIKVPK | DRB1_16 | 0.00% |
|  |  |  | VKIPVAIKVPKAN | DRB1_01 | 6.04% |
|  |  |  | VKIPVAIKVPKAN | DRB1_08 | 4.92% |
|  |  |  | VKIPVAIKVPKAN | DRB1_09 | 0.00% |
|  |  |  | VKIPVAIKVPKAN | DRB1_11 | 8.11% |
|  |  |  | VKIPVAIKVPKAN | DRB1_12 | 1.90% |
|  |  |  | VKIPVAIKVPKAN | DRB1_13 | 0.00% |
|  |  |  | VKIPVAIKVPKAN | DRB1_14 | 13.50% |
|  |  |  | VKIPVAIKVPKAN | DRB1_15 | 0.00% |
|  |  |  | VKIPVAIKVPKAN | DRB1_16 | 0.00% |
|  |  |  | GEKVKIPVAIKVP | DRB1_01 | 6.04% |
|  |  |  | GEKVKIPVAIKVP | DRB1_07 | 0.00% |
|  |  |  | GEKVKIPVAIKVP | DRB1_08 | 4.92% |
|  |  |  | GEKVKIPVAIKVP | DRB1_09 | 0.00% |
|  |  |  | GEKVKIPVAIKVP | DRB1_11 | 8.11% |
|  |  |  | GEKVKIPVAIKVP | DRB1_12 | 1.90% |
|  |  |  | GEKVKIPVAIKVP | DRB1_13 | 0.00% |
|  |  |  | GEKVKIPVAIKVP | DRB1_14 | 13.50% |
|  |  |  | GEKVKIPVAIKVP | DRB1_15 | 0.00% |
|  |  |  | KIPVAIKVPKANKE | DRB1_01 | 6.04% |
|  |  |  | KIPVAIKVPKANKE | DRB1_08 | 4.92% |
|  |  |  | KIPVAIKVPKANKE | DRB1_09 | 0.00% |
|  |  |  | KIPVAIKVPKANKE | DRB1_11 | 8.11% |
|  |  |  | KIPVAIKVPKANKE | DRB1_12 | 1.90% |
|  |  |  | KIPVAIKVPKANKE | DRB1_13 | 0.00% |
|  |  |  | KIPVAIKVPKANKE | DRB1_14 | 13.50% |
|  |  |  | KIPVAIKVPKANKE | DRB1_15 | 0.00% |
|  |  |  | KIPVAIKVPKANKE | DRB1_16 | 0.00% |
|  |  |  | KIPVAIKVPKANK | DRB1_01 | 6.04% |
|  |  |  | KIPVAIKVPKANK | DRB1_08 | 4.92% |
|  |  |  | KIPVAIKVPKANK | DRB1_09 | 0.00% |
|  |  |  | KIPVAIKVPKANK | DRB1_11 | 8.11% |
|  |  |  | KIPVAIKVPKANK | DRB1_12 | 1.90% |
|  |  |  | KIPVAIKVPKANK | DRB1_13 | 0.00% |
|  |  |  | KIPVAIKVPKANK | DRB1_14 | 13.50% |
|  |  |  | KIPVAIKVPKANK | DRB1_16 | 0.00% |
|  |  |  | KVKIPVAIKVPK | DRB1_01 | 6.04% |
|  |  |  | KVKIPVAIKVPK | DRB1_08 | 4.92% |
|  |  |  | KVKIPVAIKVPK | DRB1_09 | 0.00% |
|  |  |  | KVKIPVAIKVPK | DRB1_11 | 8.11% |
|  |  |  | KVKIPVAIKVPK | DRB1_12 | 1.90% |
|  |  |  | KVKIPVAIKVPK | DRB1_13 | 0.00% |
|  |  |  | KVKIPVAIKVPK | DRB1_14 | 13.50% |
|  |  |  | KVKIPVAIKVPK | DRB1_15 | 0.00% |
|  |  |  | KVKIPVAIKVPK | DRB1_16 | 0.00% |
|  |  |  | EGEKVKIPVAIKVP | DRB1_01 | 6.04% |
|  |  |  | EGEKVKIPVAIKVP | DRB1_07 | 0.00% |
|  |  |  | EGEKVKIPVAIKVP | DRB1_08 | 4.92% |
|  |  |  | EGEKVKIPVAIKVP | DRB1_09 | 0.00% |
|  |  |  | EGEKVKIPVAIKVP | DRB1_11 | 2.57% |
|  |  |  | EGEKVKIPVAIKVP | DRB1_12 | 1.90% |
|  |  |  | EGEKVKIPVAIKVP | DRB1_13 | 0.00% |
|  |  |  | EGEKVKIPVAIKVP | DRB1_14 | 13.50% |
|  |  |  | EGEKVKIPVAIKVP | DRB1_15 | 0.00% |
|  |  |  | IPVAIKVPKANKEI | DRB1_01 | 2.02% |
|  |  |  | IPVAIKVPKANKEI | DRB1_08 | 4.92% |
|  |  |  | IPVAIKVPKANKEI | DRB1_09 | 0.00% |
|  |  |  | IPVAIKVPKANKEI | DRB1_11 | 8.11% |
|  |  |  | IPVAIKVPKANKEI | DRB1_12 | 1.90% |
|  |  |  | IPVAIKVPKANKEI | DRB1_13 | 0.00% |
|  |  |  | IPVAIKVPKANKEI | DRB1_14 | 13.50% |
|  |  |  | IPVAIKVPKANKEI | DRB1_16 | 0.00% |
|  |  |  | PVAIKVPKANKEIL | DRB1_01 | 2.02% |
|  |  |  | PVAIKVPKANKEIL | DRB1_08 | 4.92% |
|  |  |  | PVAIKVPKANKEIL | DRB1_09 | 0.00% |
|  |  |  | PVAIKVPKANKEIL | DRB1_11 | 8.11% |
|  |  |  | PVAIKVPKANKEIL | DRB1_12 | 1.90% |
|  |  |  | PVAIKVPKANKEIL | DRB1_13 | 0.00% |
|  |  |  | PVAIKVPKANKEIL | DRB1_14 | 13.50% |
|  |  |  | EKVKIPVAIKVP | DRB1_01 | 6.04% |
|  |  |  | EKVKIPVAIKVP | DRB1_08 | 4.92% |
|  |  |  | EKVKIPVAIKVP | DRB1_09 | 0.00% |
|  |  |  | EKVKIPVAIKVP | DRB1_11 | 2.57% |
|  |  |  | EKVKIPVAIKVP | DRB1_12 | 1.90% |
|  |  |  | EKVKIPVAIKVP | DRB1_13 | 0.00% |
|  |  |  | EKVKIPVAIKVP | DRB1_14 | 12.37% |
|  |  |  | EKVKIPVAIKVP | DRB1_15 | 0.00% |
|  |  |  | IPVAIKVPKANKE | DRB1_01 | 2.02% |
|  |  |  | IPVAIKVPKANKE | DRB1_08 | 4.92% |
|  |  |  | IPVAIKVPKANKE | DRB1_11 | 8.11% |
|  |  |  | IPVAIKVPKANKE | DRB1_12 | 1.90% |
|  |  |  | IPVAIKVPKANKE | DRB1_13 | 0.00% |
|  |  |  | IPVAIKVPKANKE | DRB1_14 | 12.37% |
|  |  |  | KIPVAIKVPKAN | DRB1_01 | 2.02% |
|  |  |  | KIPVAIKVPKAN | DRB1_08 | 4.92% |
|  |  |  | KIPVAIKVPKAN | DRB1_11 | 8.11% |
|  |  |  | KIPVAIKVPKAN | DRB1_12 | 1.90% |
|  |  |  | KIPVAIKVPKAN | DRB1_13 | 0.00% |
|  |  |  | KIPVAIKVPKAN | DRB1_14 | 5.38% |
|  |  |  | IPVAIKVPKANK | DRB1_01 | 2.02% |
|  |  |  | IPVAIKVPKANK | DRB1_08 | 4.92% |
|  |  |  | IPVAIKVPKANK | DRB1_11 | 8.11% |
|  |  |  | IPVAIKVPKANK | DRB1_12 | 1.90% |
|  |  |  | IPVAIKVPKANK | DRB1_13 | 0.00% |
|  |  |  | IPVAIKVPKANK | DRB1_14 | 5.38% |
|  |  |  | VKIPVAIKVPKA | DRB1_01 | 2.02% |
|  |  |  | VKIPVAIKVPKA | DRB1_08 | 4.92% |
|  |  |  | VKIPVAIKVPKA | DRB1_11 | 2.57% |
|  |  |  | VKIPVAIKVPKA | DRB1_12 | 1.90% |
|  |  |  | VKIPVAIKVPKA | DRB1_13 | 0.00% |
|  |  |  | VKIPVAIKVPKA | DRB1_14 | 5.38% |
|  |  |  | KVKIPVAIKVP | DRB1_01 | 2.02% |
|  |  |  | PVAIKVPKANKEI | DRB1_01 | 2.02% |
|  |  |  | KVKIPVAIKVP | DRB1_08 | 4.92% |
|  |  |  | PVAIKVPKANKEI | DRB1_08 | 4.92% |
|  |  |  | KVKIPVAIKVP | DRB1_09 | 0.00% |
|  |  |  | KVKIPVAIKVP | DRB1_11 | 2.57% |
|  |  |  | PVAIKVPKANKEI | DRB1_11 | 2.57% |
|  |  |  | KVKIPVAIKVP | DRB1_12 | 1.90% |
|  |  |  | PVAIKVPKANKEI | DRB1_12 | 1.90% |
|  |  |  | KVKIPVAIKVP | DRB1_13 | 0.00% |
|  |  |  | PVAIKVPKANKEI | DRB1_13 | 0.00% |
|  |  |  | KVKIPVAIKVP | DRB1_14 | 5.38% |
|  |  |  | PVAIKVPKANKEI | DRB1_14 | 5.38% |
|  |  |  | VAIKVPKANKEILD | DRB1_01 | 2.02% |
|  |  |  | VAIKVPKANKEILD | DRB1_08 | 4.92% |
|  |  |  | VAIKVPKANKEILD | DRB1_11 | 2.57% |
|  |  |  | VAIKVPKANKEILD | DRB1_12 | 1.90% |
|  |  |  | VAIKVPKANKEILD | DRB1_13 | 0.00% |
|  |  |  | VAIKVPKANKEILD | DRB1_14 | 5.38% |
|  |  |  | VAIKVPKANKEIL | DRB1_01 | 2.02% |
|  |  |  | VAIKVPKANKEIL | DRB1_08 | 4.92% |
|  |  |  | VAIKVPKANKEIL | DRB1_11 | 2.57% |
|  |  |  | VAIKVPKANKEIL | DRB1_12 | 1.90% |
|  |  |  | VAIKVPKANKEIL | DRB1_13 | 0.00% |
|  |  |  | VAIKVPKANKEIL | DRB1_14 | 5.38% |
|  |  |  | KIPVAIKVPKA | DRB1_01 | 2.02% |
|  |  |  | KIPVAIKVPKA | DRB1_08 | 4.92% |
|  |  |  | KIPVAIKVPKA | DRB1_11 | 2.57% |
|  |  |  | KIPVAIKVPKA | DRB1_12 | 1.90% |
|  |  |  | KIPVAIKVPKA | DRB1_13 | 0.00% |
|  |  |  | KIPVAIKVPKA | DRB1_14 | 5.38% |
|  |  |  | IPVAIKVPKAN | DRB1_01 | 2.02% |
|  |  |  | IPVAIKVPKAN | DRB1_08 | 4.92% |
|  |  |  | IPVAIKVPKAN | DRB1_11 | 2.57% |
|  |  |  | IPVAIKVPKAN | DRB1_12 | 1.90% |
|  |  |  | IPVAIKVPKAN | DRB1_13 | 0.00% |
|  |  |  | IPVAIKVPKAN | DRB1_14 | 5.38% |
|  |  |  | PVAIKVPKANKE | DRB1_01 | 0.00% |
|  |  |  | PVAIKVPKANKE | DRB1_08 | 4.92% |
|  |  |  | PVAIKVPKANKE | DRB1_11 | 2.57% |
|  |  |  | PVAIKVPKANKE | DRB1_12 | 0.00% |
|  |  |  | PVAIKVPKANKE | DRB1_13 | 0.00% |
|  |  |  | PVAIKVPKANKE | DRB1_14 | 5.38% |
|  |  |  | VKIPVAIKVPK | DRB1_01 | 0.00% |
|  |  |  | VKIPVAIKVPK | DRB1_08 | 2.29% |
|  |  |  | VKIPVAIKVPK | DRB1_11 | 2.57% |
|  |  |  | VKIPVAIKVPK | DRB1_12 | 1.90% |
|  |  |  | VKIPVAIKVPK | DRB1_13 | 0.00% |
|  |  |  | VKIPVAIKVPK | DRB1_14 | 5.38% |
|  |  |  | VAIKVPKANKEI | DRB1_01 | 0.00% |
|  |  |  | VAIKVPKANKEI | DRB1_08 | 4.92% |
|  |  |  | VAIKVPKANKEI | DRB1_11 | 2.57% |
|  |  |  | VAIKVPKANKEI | DRB1_12 | 0.00% |
|  |  |  | VAIKVPKANKEI | DRB1_13 | 0.00% |
|  |  |  | VAIKVPKANKEI | DRB1_14 | 5.38% |
|  |  |  | PVAIKVPKANK | DRB1_01 | 0.00% |
|  |  |  | PVAIKVPKANK | DRB1_08 | 4.92% |
|  |  |  | PVAIKVPKANK | DRB1_11 | 2.57% |
|  |  |  | PVAIKVPKANK | DRB1_12 | 0.00% |
|  |  |  | PVAIKVPKANK | DRB1_13 | 0.00% |
|  |  |  | PVAIKVPKANK | DRB1_14 | 5.38% |
|  |  |  | VAIKVPKANKE | DRB1_08 | 0.00% |
|  |  |  | VAIKVPKANKE | DRB1_11 | 2.57% |
|  |  |  | VAIKVPKANKE | DRB1_13 | 0.00% |
|  |  |  | VAIKVPKANKE | DRB1_14 | 5.38% |
|  |  |  | IPVAIKVPKA | DRB1_08 | 2.63% |
|  |  |  | IPVAIKVPKA | DRB1_11 | 0.00% |
|  |  |  | IPVAIKVPKA | DRB1_12 | 0.00% |
|  |  |  | IPVAIKVPKA | DRB1_13 | 0.00% |
|  |  |  | IPVAIKVPKA | DRB1_14 | 5.38% |
|  |  |  | AIKVPKANKEIL | DRB1_08 | 0.00% |
|  |  |  | AIKVPKANKEILD | DRB1_08 | 0.00% |
|  |  |  | AIKVPKANKEIL | DRB1_11 | 0.00% |
|  |  |  | AIKVPKANKEILD | DRB1_11 | 0.00% |
|  |  |  | AIKVPKANKEIL | DRB1_12 | 0.00% |
|  |  |  | AIKVPKANKEILD | DRB1_12 | 0.00% |
|  |  |  | AIKVPKANKEIL | DRB1_13 | 0.00% |
|  |  |  | AIKVPKANKEILD | DRB1_13 | 0.00% |
|  |  |  | AIKVPKANKEIL | DRB1_14 | 5.38% |
|  |  |  | AIKVPKANKEILD | DRB1_14 | 5.38% |
|  |  |  | AIKVPKANKEILDE | DRB1_08 | 0.00% |
|  |  |  | AIKVPKANKEILDE | DRB1_11 | 0.00% |
|  |  |  | AIKVPKANKEILDE | DRB1_12 | 0.00% |
|  |  |  | AIKVPKANKEILDE | DRB1_13 | 0.00% |
|  |  |  | AIKVPKANKEILDE | DRB1_14 | 5.38% |
|  |  |  | AIKVPKANKEI | DRB1_08 | 0.00% |
|  |  |  | AIKVPKANKEI | DRB1_13 | 0.00% |
|  |  |  | AIKVPKANKEI | DRB1_14 | 0.00% |
|  |  |  | PVAIKVPKAN | DRB1_08 | 0.00% |
|  |  |  | PVAIKVPKAN | DRB1_13 | 0.00% |
|  |  |  | PVAIKVPKAN | DRB1_14 | 0.00% |
|  |  |  | KIPVAIKVPK | DRB1_08 | 0.00% |
|  |  |  | VAIKVPKANK | DRB1_08 | 0.00% |
|  |  |  | KIPVAIKVPK | DRB1_13 | 0.00% |
|  |  |  | VAIKVPKANK | DRB1_13 | 0.00% |
|  |  |  | KIPVAIKVPK | DRB1_14 | 0.00% |
|  |  |  | VAIKVPKANK | DRB1_14 | 0.00% |
|  |  |  | VKIPVAIKVP | DRB1_08 | 0.00% |
|  |  |  | VKIPVAIKVP | DRB1_13 | 0.00% |
|  |  |  | IKVPKANKEILDEA | DRB1_08 | 0.00% |
|  |  |  | IKVPKANKEILDEA | DRB1_13 | 0.00% |
|  |  |  | AIKVPKANKE | DRB1_08 | 0.00% |
|  |  |  | IKVPKANKEILD | DRB1_08 | 0.00% |
|  |  |  | IKVPKANKEILDE | DRB1_08 | 0.00% |
|  |  |  | IKVPKANKEILD | DRB1_13 | 0.00% |
|  |  |  | IKVPKANKEILDE | DRB1_13 | 0.00% |
|  |  |  | IKVPKANKEIL | DRB1_08 | 0.00% |
| Total |  | 60.96% |  |  | 64.94% |
