# Supplementary material for: Immunogenicity of Del19 EGFR mutations in Chinese patients affected by lung adenocarcinoma
Source: BMC Immunol. 2019 Nov 13;20:43. doi: 10.1186/s12865-019-0320-1 (PMC6854806; doi:10.1186/s12865-019-0320-1)
Supplement: Supplementary file 7 — Additional file 7. Predicted HLA binding epitopes for EGFR delE746_P753insVS. [file 12865_2019_320_MOESM7_ESM.doc]

**Supplemental Table 7, Predicted HLA binding epitopes for EGFR delE746_P753insVS by Chinese NSCLC patients as predicted by NetMHC4.0.** The percentages are the total frequencies of HLA alleles which may present a mutant EGFR.

| Class I | | | Class II | | |
| --- | --- | --- | --- | --- | --- |
| Neopeptide | HLA alleles | Frequency | Neopeptide | HLA alleles | Frequency |
| KIPVAIKVSK | HLA-A*74 | 0.00% | PVAIKVSKANKEIL | DRB1_01 | 6.04% |
| KIPVAIKVSK | HLA-A*68 | 0.10% | PVAIKVSKANKEIL | DRB1_03 | 0.00% |
| KIPVAIKVSK | HLA-A*31 | 0.32% | PVAIKVSKANKEIL | DRB1_04 | 2.25% |
| KIPVAIKVSK | HLA-A*30 | 7.56% | PVAIKVSKANKEIL | DRB1_07 | 8.50% |
| KIPVAIKVSK | HLA-A*11 | 25.85% | PVAIKVSKANKEIL | DRB1_08 | 4.92% |
| KIPVAIKVSK | HLA-A*03 | 3.64% | PVAIKVSKANKEIL | DRB1_09 | 2.82% |
| AIKVSKANK | HLA-A*68 | 0.00% | PVAIKVSKANKEIL | DRB1_10 | 0.00% |
| AIKVSKANK | HLA-A*31 | 0.18% | PVAIKVSKANKEIL | DRB1_11 | 8.11% |
| AIKVSKANK | HLA-A*30 | 7.56% | PVAIKVSKANKEIL | DRB1_12 | 1.90% |
| AIKVSKANK | HLA-A*11 | 0.66% | PVAIKVSKANKEIL | DRB1_13 | 6.83% |
| AIKVSKANK | HLA-A*03 | 2.88% | PVAIKVSKANKEIL | DRB1_14 | 21.90% |
| VAIKVSKANK | HLA-A*68 | 0.85% | PVAIKVSKANKEIL | DRB1_15 | 0.00% |
| VAIKVSKANK | HLA-A*34 | 0.00% | PVAIKVSKANKEIL | DRB1_16 | 6.81% |
| VAIKVSKANK | HLA-A*30 | 0.00% | VAIKVSKANKEIL | DRB1_01 | 6.04% |
| VAIKVSKANK | HLA-A*11 | 25.55% | VAIKVSKANKEIL | DRB1_03 | 0.00% |
| VAIKVSKANK | HLA-A*03 | 0.00% | VAIKVSKANKEIL | DRB1_04 | 2.25% |
| IPVAIKVSKA | HLA-B*56 | 0.84% | VAIKVSKANKEIL | DRB1_07 | 8.50% |
| IPVAIKVSKA | HLA-B*55 | 3.44% | VAIKVSKANKEIL | DRB1_08 | 4.92% |
| IPVAIKVSKA | HLA-B*54 | 3.16% | VAIKVSKANKEIL | DRB1_09 | 0.00% |
| IPVAIKVSKA | HLA-B*42 | 0.00% | VAIKVSKANKEIL | DRB1_10 | 0.00% |
| IPVAIKVSKA | HLA-B*07 | 0.00% | VAIKVSKANKEIL | DRB1_11 | 8.11% |
| KVKIPVAIKV | HLA-B*27 | 0.00% | VAIKVSKANKEIL | DRB1_12 | 1.90% |
| KVKIPVAIKV | HLA-A*30 | 7.56% | VAIKVSKANKEIL | DRB1_13 | 6.83% |
| KVKIPVAIKV | HLA-A*03 | 0.00% | VAIKVSKANKEIL | DRB1_14 | 21.90% |
| KVKIPVAIKV | HLA-A*02 | 9.17% | VAIKVSKANKEIL | DRB1_15 | 0.00% |
| KVKIPVAIKV | HLA-A*024 | 0.00% | VAIKVSKANKEIL | DRB1_16 | 0.00% |
| KVKIPVAIKV | HLA-A*029 | 0.00% | VAIKVSKANKEILD | DRB1_01 | 6.04% |
| KVKIPVAIKV | HLA-A*028 | 0.00% | VAIKVSKANKEILD | DRB1_03 | 0.00% |
| KVKIPVAIKV | HLA-A*023 | 0.00% | VAIKVSKANKEILD | DRB1_04 | 2.25% |
| KVKIPVAIKV | HLA-A*020 | 0.00% | VAIKVSKANKEILD | DRB1_07 | 8.50% |
| KVKIPVAIKV | HLA-A*022 | 0.00% | VAIKVSKANKEILD | DRB1_08 | 4.92% |
| KVKIPVAIKV | HLA-A*027 | 0.00% | VAIKVSKANKEILD | DRB1_09 | 0.00% |
| KVKIPVAIKV | HLA-A*026 | 0.00% | VAIKVSKANKEILD | DRB1_10 | 0.00% |
| KVKIPVAIKV | HLA-A*025 | 0.00% | VAIKVSKANKEILD | DRB1_11 | 8.11% |
| IPVAIKVSK | HLA-B*55 | 0.00% | VAIKVSKANKEILD | DRB1_12 | 1.90% |
|  |  |  | VAIKVSKANKEILD | DRB1_13 | 6.83% |
|  |  |  | VAIKVSKANKEILD | DRB1_14 | 21.90% |
|  |  |  | VAIKVSKANKEILD | DRB1_15 | 0.00% |
|  |  |  | VAIKVSKANKEILD | DRB1_16 | 0.00% |
|  |  |  | IPVAIKVSKANKEI | DRB1_01 | 6.04% |
|  |  |  | IPVAIKVSKANKEI | DRB1_03 | 0.00% |
|  |  |  | IPVAIKVSKANKEI | DRB1_04 | 2.25% |
|  |  |  | IPVAIKVSKANKEI | DRB1_07 | 8.50% |
|  |  |  | IPVAIKVSKANKEI | DRB1_08 | 16.05% |
|  |  |  | IPVAIKVSKANKEI | DRB1_09 | 0.00% |
|  |  |  | IPVAIKVSKANKEI | DRB1_11 | 8.11% |
|  |  |  | IPVAIKVSKANKEI | DRB1_12 | 1.90% |
|  |  |  | IPVAIKVSKANKEI | DRB1_13 | 6.83% |
|  |  |  | IPVAIKVSKANKEI | DRB1_14 | 19.14% |
|  |  |  | IPVAIKVSKANKEI | DRB1_15 | 0.00% |
|  |  |  | IPVAIKVSKANKEI | DRB1_16 | 6.81% |
|  |  |  | EKVKIPVAIKVSKA | DRB1_01 | 6.04% |
|  |  |  | EKVKIPVAIKVSKA | DRB1_04 | 0.00% |
|  |  |  | EKVKIPVAIKVSKA | DRB1_07 | 8.50% |
|  |  |  | EKVKIPVAIKVSKA | DRB1_08 | 4.92% |
|  |  |  | EKVKIPVAIKVSKA | DRB1_09 | 2.82% |
|  |  |  | EKVKIPVAIKVSKA | DRB1_11 | 8.11% |
|  |  |  | EKVKIPVAIKVSKA | DRB1_12 | 1.90% |
|  |  |  | EKVKIPVAIKVSKA | DRB1_13 | 4.03% |
|  |  |  | EKVKIPVAIKVSKA | DRB1_14 | 13.50% |
|  |  |  | EKVKIPVAIKVSKA | DRB1_15 | 0.00% |
|  |  |  | EKVKIPVAIKVSKA | DRB1_16 | 6.81% |
|  |  |  | GEKVKIPVAIKVSK | DRB1_01 | 6.04% |
|  |  |  | GEKVKIPVAIKVSK | DRB1_07 | 8.50% |
|  |  |  | GEKVKIPVAIKVSK | DRB1_08 | 4.92% |
|  |  |  | GEKVKIPVAIKVSK | DRB1_09 | 2.82% |
|  |  |  | GEKVKIPVAIKVSK | DRB1_11 | 8.11% |
|  |  |  | GEKVKIPVAIKVSK | DRB1_12 | 1.90% |
|  |  |  | GEKVKIPVAIKVSK | DRB1_13 | 4.03% |
|  |  |  | GEKVKIPVAIKVSK | DRB1_14 | 13.50% |
|  |  |  | GEKVKIPVAIKVSK | DRB1_15 | 0.00% |
|  |  |  | GEKVKIPVAIKVSK | DRB1_16 | 6.81% |
|  |  |  | PVAIKVSKANKEI | DRB1_01 | 6.04% |
|  |  |  | PVAIKVSKANKEI | DRB1_03 | 0.00% |
|  |  |  | PVAIKVSKANKEI | DRB1_04 | 2.25% |
|  |  |  | PVAIKVSKANKEI | DRB1_07 | 8.50% |
|  |  |  | PVAIKVSKANKEI | DRB1_08 | 4.92% |
|  |  |  | PVAIKVSKANKEI | DRB1_09 | 0.00% |
|  |  |  | PVAIKVSKANKEI | DRB1_11 | 8.11% |
|  |  |  | PVAIKVSKANKEI | DRB1_12 | 1.90% |
|  |  |  | PVAIKVSKANKEI | DRB1_13 | 4.03% |
|  |  |  | PVAIKVSKANKEI | DRB1_14 | 13.50% |
|  |  |  | PVAIKVSKANKEI | DRB1_15 | 0.00% |
|  |  |  | PVAIKVSKANKEI | DRB1_16 | 0.00% |
|  |  |  | KVKIPVAIKVSKAN | DRB1_01 | 6.04% |
|  |  |  | KVKIPVAIKVSKAN | DRB1_04 | 0.00% |
|  |  |  | KVKIPVAIKVSKAN | DRB1_07 | 8.50% |
|  |  |  | KVKIPVAIKVSKAN | DRB1_08 | 16.05% |
|  |  |  | KVKIPVAIKVSKAN | DRB1_09 | 0.00% |
|  |  |  | KVKIPVAIKVSKAN | DRB1_11 | 8.11% |
|  |  |  | KVKIPVAIKVSKAN | DRB1_12 | 1.90% |
|  |  |  | KVKIPVAIKVSKAN | DRB1_13 | 0.00% |
|  |  |  | KVKIPVAIKVSKAN | DRB1_14 | 13.50% |
|  |  |  | KVKIPVAIKVSKAN | DRB1_15 | 0.00% |
|  |  |  | KVKIPVAIKVSKAN | DRB1_16 | 6.81% |
|  |  |  | KVKIPVAIKVSKA | DRB1_01 | 6.04% |
|  |  |  | KVKIPVAIKVSKA | DRB1_07 | 8.50% |
|  |  |  | KVKIPVAIKVSKA | DRB1_08 | 4.92% |
|  |  |  | KVKIPVAIKVSKA | DRB1_09 | 0.00% |
|  |  |  | KVKIPVAIKVSKA | DRB1_11 | 8.11% |
|  |  |  | KVKIPVAIKVSKA | DRB1_12 | 1.90% |
|  |  |  | KVKIPVAIKVSKA | DRB1_13 | 0.00% |
|  |  |  | KVKIPVAIKVSKA | DRB1_14 | 13.50% |
|  |  |  | KVKIPVAIKVSKA | DRB1_15 | 0.00% |
|  |  |  | KVKIPVAIKVSKA | DRB1_16 | 6.81% |
|  |  |  | EKVKIPVAIKVSK | DRB1_01 | 6.04% |
|  |  |  | EKVKIPVAIKVSK | DRB1_07 | 8.50% |
|  |  |  | EKVKIPVAIKVSK | DRB1_08 | 4.92% |
|  |  |  | EKVKIPVAIKVSK | DRB1_09 | 0.00% |
|  |  |  | EKVKIPVAIKVSK | DRB1_11 | 8.11% |
|  |  |  | EKVKIPVAIKVSK | DRB1_12 | 1.90% |
|  |  |  | EKVKIPVAIKVSK | DRB1_13 | 0.00% |
|  |  |  | EKVKIPVAIKVSK | DRB1_14 | 13.50% |
|  |  |  | EKVKIPVAIKVSK | DRB1_15 | 0.00% |
|  |  |  | EKVKIPVAIKVSK | DRB1_16 | 6.81% |
|  |  |  | VAIKVSKANKEI | DRB1_01 | 6.04% |
|  |  |  | AIKVSKANKEIL | DRB1_01 | 6.04% |
|  |  |  | VAIKVSKANKEI | DRB1_03 | 0.00% |
|  |  |  | AIKVSKANKEIL | DRB1_03 | 0.00% |
|  |  |  | VAIKVSKANKEI | DRB1_04 | 0.00% |
|  |  |  | AIKVSKANKEIL | DRB1_04 | 0.00% |
|  |  |  | VAIKVSKANKEI | DRB1_07 | 0.00% |
|  |  |  | AIKVSKANKEIL | DRB1_07 | 0.00% |
|  |  |  | VAIKVSKANKEI | DRB1_08 | 4.92% |
|  |  |  | AIKVSKANKEIL | DRB1_08 | 4.92% |
|  |  |  | VAIKVSKANKEI | DRB1_09 | 0.00% |
|  |  |  | AIKVSKANKEIL | DRB1_09 | 0.00% |
|  |  |  | VAIKVSKANKEI | DRB1_11 | 8.11% |
|  |  |  | AIKVSKANKEIL | DRB1_11 | 8.11% |
|  |  |  | VAIKVSKANKEI | DRB1_12 | 1.90% |
|  |  |  | AIKVSKANKEIL | DRB1_12 | 1.90% |
|  |  |  | VAIKVSKANKEI | DRB1_13 | 4.03% |
|  |  |  | AIKVSKANKEIL | DRB1_13 | 4.03% |
|  |  |  | VAIKVSKANKEI | DRB1_14 | 13.50% |
|  |  |  | AIKVSKANKEIL | DRB1_14 | 13.50% |
|  |  |  | VAIKVSKANKEI | DRB1_15 | 0.00% |
|  |  |  | AIKVSKANKEIL | DRB1_15 | 0.00% |
|  |  |  | KIPVAIKVSKANKE | DRB1_01 | 6.04% |
|  |  |  | KIPVAIKVSKANKE | DRB1_04 | 0.00% |
|  |  |  | KIPVAIKVSKANKE | DRB1_07 | 0.00% |
|  |  |  | KIPVAIKVSKANKE | DRB1_08 | 16.05% |
|  |  |  | KIPVAIKVSKANKE | DRB1_09 | 0.00% |
|  |  |  | KIPVAIKVSKANKE | DRB1_11 | 8.11% |
|  |  |  | KIPVAIKVSKANKE | DRB1_12 | 1.90% |
|  |  |  | KIPVAIKVSKANKE | DRB1_13 | 0.00% |
|  |  |  | KIPVAIKVSKANKE | DRB1_14 | 13.50% |
|  |  |  | KIPVAIKVSKANKE | DRB1_15 | 0.00% |
|  |  |  | KIPVAIKVSKANKE | DRB1_16 | 6.81% |
|  |  |  | VKIPVAIKVSKANK | DRB1_01 | 6.04% |
|  |  |  | VKIPVAIKVSKANK | DRB1_04 | 0.00% |
|  |  |  | VKIPVAIKVSKANK | DRB1_07 | 0.00% |
|  |  |  | VKIPVAIKVSKANK | DRB1_08 | 16.05% |
|  |  |  | VKIPVAIKVSKANK | DRB1_09 | 0.00% |
|  |  |  | VKIPVAIKVSKANK | DRB1_11 | 8.11% |
|  |  |  | VKIPVAIKVSKANK | DRB1_12 | 1.90% |
|  |  |  | VKIPVAIKVSKANK | DRB1_13 | 0.00% |
|  |  |  | VKIPVAIKVSKANK | DRB1_14 | 13.50% |
|  |  |  | VKIPVAIKVSKANK | DRB1_15 | 0.00% |
|  |  |  | VKIPVAIKVSKANK | DRB1_16 | 6.81% |
|  |  |  | GEKVKIPVAIKVS | DRB1_01 | 6.04% |
|  |  |  | GEKVKIPVAIKVS | DRB1_07 | 8.50% |
|  |  |  | GEKVKIPVAIKVS | DRB1_08 | 4.92% |
|  |  |  | GEKVKIPVAIKVS | DRB1_09 | 0.00% |
|  |  |  | GEKVKIPVAIKVS | DRB1_11 | 8.11% |
|  |  |  | GEKVKIPVAIKVS | DRB1_12 | 1.90% |
|  |  |  | GEKVKIPVAIKVS | DRB1_13 | 0.00% |
|  |  |  | GEKVKIPVAIKVS | DRB1_14 | 13.50% |
|  |  |  | GEKVKIPVAIKVS | DRB1_15 | 0.00% |
|  |  |  | GEKVKIPVAIKVS | DRB1_16 | 0.00% |
|  |  |  | EGEKVKIPVAIKVS | DRB1_01 | 6.04% |
|  |  |  | EGEKVKIPVAIKVS | DRB1_07 | 8.50% |
|  |  |  | EGEKVKIPVAIKVS | DRB1_08 | 4.92% |
|  |  |  | EGEKVKIPVAIKVS | DRB1_09 | 0.00% |
|  |  |  | EGEKVKIPVAIKVS | DRB1_11 | 8.11% |
|  |  |  | EGEKVKIPVAIKVS | DRB1_12 | 1.90% |
|  |  |  | EGEKVKIPVAIKVS | DRB1_13 | 0.00% |
|  |  |  | EGEKVKIPVAIKVS | DRB1_14 | 13.50% |
|  |  |  | EGEKVKIPVAIKVS | DRB1_15 | 0.00% |
|  |  |  | EGEKVKIPVAIKVS | DRB1_16 | 0.00% |
|  |  |  | AIKVSKANKEILD | DRB1_01 | 6.04% |
|  |  |  | AIKVSKANKEILD | DRB1_03 | 0.00% |
|  |  |  | AIKVSKANKEILD | DRB1_04 | 0.00% |
|  |  |  | AIKVSKANKEILD | DRB1_07 | 0.00% |
|  |  |  | AIKVSKANKEILD | DRB1_08 | 4.92% |
|  |  |  | AIKVSKANKEILD | DRB1_09 | 0.00% |
|  |  |  | AIKVSKANKEILD | DRB1_11 | 2.57% |
|  |  |  | AIKVSKANKEILD | DRB1_12 | 1.90% |
|  |  |  | AIKVSKANKEILD | DRB1_13 | 4.03% |
|  |  |  | AIKVSKANKEILD | DRB1_14 | 13.50% |
|  |  |  | AIKVSKANKEILD | DRB1_15 | 0.00% |
|  |  |  | IPVAIKVSKANKE | DRB1_01 | 6.04% |
|  |  |  | IPVAIKVSKANKE | DRB1_04 | 0.00% |
|  |  |  | IPVAIKVSKANKE | DRB1_07 | 0.00% |
|  |  |  | IPVAIKVSKANKE | DRB1_08 | 4.92% |
|  |  |  | IPVAIKVSKANKE | DRB1_09 | 0.00% |
|  |  |  | IPVAIKVSKANKE | DRB1_11 | 8.11% |
|  |  |  | IPVAIKVSKANKE | DRB1_12 | 1.90% |
|  |  |  | IPVAIKVSKANKE | DRB1_13 | 0.00% |
|  |  |  | IPVAIKVSKANKE | DRB1_14 | 13.50% |
|  |  |  | IPVAIKVSKANKE | DRB1_15 | 0.00% |
|  |  |  | IPVAIKVSKANKE | DRB1_16 | 0.00% |
|  |  |  | AIKVSKANKEILDE | DRB1_01 | 6.04% |
|  |  |  | AIKVSKANKEILDE | DRB1_03 | 0.00% |
|  |  |  | AIKVSKANKEILDE | DRB1_04 | 0.00% |
|  |  |  | AIKVSKANKEILDE | DRB1_07 | 0.00% |
|  |  |  | AIKVSKANKEILDE | DRB1_08 | 4.92% |
|  |  |  | AIKVSKANKEILDE | DRB1_09 | 0.00% |
|  |  |  | AIKVSKANKEILDE | DRB1_11 | 2.57% |
|  |  |  | AIKVSKANKEILDE | DRB1_12 | 1.90% |
|  |  |  | AIKVSKANKEILDE | DRB1_13 | 4.03% |
|  |  |  | AIKVSKANKEILDE | DRB1_14 | 13.50% |
|  |  |  | AIKVSKANKEILDE | DRB1_15 | 0.00% |
|  |  |  | KIPVAIKVSKANK | DRB1_01 | 6.04% |
|  |  |  | KIPVAIKVSKANK | DRB1_08 | 16.05% |
|  |  |  | KIPVAIKVSKANK | DRB1_09 | 0.00% |
|  |  |  | KIPVAIKVSKANK | DRB1_11 | 8.11% |
|  |  |  | KIPVAIKVSKANK | DRB1_12 | 1.90% |
|  |  |  | KIPVAIKVSKANK | DRB1_13 | 0.00% |
|  |  |  | KIPVAIKVSKANK | DRB1_14 | 13.50% |
|  |  |  | KIPVAIKVSKANK | DRB1_16 | 6.81% |
|  |  |  | KVKIPVAIKVSK | DRB1_01 | 6.04% |
|  |  |  | KVKIPVAIKVSK | DRB1_07 | 0.00% |
|  |  |  | KVKIPVAIKVSK | DRB1_08 | 4.92% |
|  |  |  | KVKIPVAIKVSK | DRB1_09 | 0.00% |
|  |  |  | KVKIPVAIKVSK | DRB1_11 | 8.11% |
|  |  |  | KVKIPVAIKVSK | DRB1_12 | 1.90% |
|  |  |  | KVKIPVAIKVSK | DRB1_13 | 0.00% |
|  |  |  | KVKIPVAIKVSK | DRB1_14 | 13.50% |
|  |  |  | KVKIPVAIKVSK | DRB1_15 | 0.00% |
|  |  |  | KVKIPVAIKVSK | DRB1_16 | 0.00% |
|  |  |  | EKVKIPVAIKVS | DRB1_01 | 6.04% |
|  |  |  | EKVKIPVAIKVS | DRB1_07 | 0.00% |
|  |  |  | EKVKIPVAIKVS | DRB1_08 | 4.92% |
|  |  |  | EKVKIPVAIKVS | DRB1_09 | 0.00% |
|  |  |  | EKVKIPVAIKVS | DRB1_11 | 8.11% |
|  |  |  | EKVKIPVAIKVS | DRB1_12 | 1.90% |
|  |  |  | EKVKIPVAIKVS | DRB1_13 | 0.00% |
|  |  |  | EKVKIPVAIKVS | DRB1_14 | 13.50% |
|  |  |  | EKVKIPVAIKVS | DRB1_15 | 0.00% |
|  |  |  | EKVKIPVAIKVS | DRB1_16 | 0.00% |
|  |  |  | VKIPVAIKVSKAN | DRB1_01 | 6.04% |
|  |  |  | VKIPVAIKVSKAN | DRB1_08 | 4.92% |
|  |  |  | VKIPVAIKVSKAN | DRB1_09 | 0.00% |
|  |  |  | VKIPVAIKVSKAN | DRB1_11 | 8.11% |
|  |  |  | VKIPVAIKVSKAN | DRB1_12 | 1.90% |
|  |  |  | VKIPVAIKVSKAN | DRB1_13 | 0.00% |
|  |  |  | VKIPVAIKVSKAN | DRB1_14 | 13.50% |
|  |  |  | VKIPVAIKVSKAN | DRB1_15 | 0.00% |
|  |  |  | VKIPVAIKVSKAN | DRB1_16 | 0.00% |
|  |  |  | PVAIKVSKANKE | DRB1_01 | 2.02% |
|  |  |  | PVAIKVSKANKE | DRB1_08 | 4.92% |
|  |  |  | PVAIKVSKANKE | DRB1_09 | 0.00% |
|  |  |  | PVAIKVSKANKE | DRB1_11 | 8.11% |
|  |  |  | PVAIKVSKANKE | DRB1_12 | 1.90% |
|  |  |  | PVAIKVSKANKE | DRB1_13 | 0.00% |
|  |  |  | PVAIKVSKANKE | DRB1_14 | 13.50% |
|  |  |  | VKIPVAIKVSKA | DRB1_01 | 6.04% |
|  |  |  | VKIPVAIKVSKA | DRB1_08 | 4.92% |
|  |  |  | VKIPVAIKVSKA | DRB1_09 | 0.00% |
|  |  |  | VKIPVAIKVSKA | DRB1_11 | 8.11% |
|  |  |  | VKIPVAIKVSKA | DRB1_12 | 1.90% |
|  |  |  | VKIPVAIKVSKA | DRB1_13 | 0.00% |
|  |  |  | VKIPVAIKVSKA | DRB1_14 | 12.37% |
|  |  |  | IPVAIKVSKANK | DRB1_01 | 2.02% |
|  |  |  | IPVAIKVSKANK | DRB1_08 | 4.92% |
|  |  |  | IPVAIKVSKANK | DRB1_09 | 0.00% |
|  |  |  | IPVAIKVSKANK | DRB1_11 | 8.11% |
|  |  |  | IPVAIKVSKANK | DRB1_12 | 1.90% |
|  |  |  | IPVAIKVSKANK | DRB1_13 | 0.00% |
|  |  |  | IPVAIKVSKANK | DRB1_14 | 13.50% |
|  |  |  | KIPVAIKVSKAN | DRB1_01 | 2.02% |
|  |  |  | KIPVAIKVSKAN | DRB1_08 | 4.92% |
|  |  |  | KIPVAIKVSKAN | DRB1_11 | 8.11% |
|  |  |  | KIPVAIKVSKAN | DRB1_12 | 1.90% |
|  |  |  | KIPVAIKVSKAN | DRB1_13 | 0.00% |
|  |  |  | KIPVAIKVSKAN | DRB1_14 | 12.37% |
|  |  |  | KVKIPVAIKVS | DRB1_01 | 6.04% |
|  |  |  | KVKIPVAIKVS | DRB1_08 | 4.92% |
|  |  |  | KVKIPVAIKVS | DRB1_09 | 0.00% |
|  |  |  | KVKIPVAIKVS | DRB1_11 | 2.57% |
|  |  |  | KVKIPVAIKVS | DRB1_12 | 1.90% |
|  |  |  | KVKIPVAIKVS | DRB1_13 | 0.00% |
|  |  |  | KVKIPVAIKVS | DRB1_14 | 5.38% |
|  |  |  | AIKVSKANKEI | DRB1_01 | 2.02% |
|  |  |  | AIKVSKANKEI | DRB1_08 | 4.92% |
|  |  |  | AIKVSKANKEI | DRB1_09 | 0.00% |
|  |  |  | AIKVSKANKEI | DRB1_11 | 2.57% |
|  |  |  | AIKVSKANKEI | DRB1_12 | 1.90% |
|  |  |  | AIKVSKANKEI | DRB1_13 | 0.00% |
|  |  |  | AIKVSKANKEI | DRB1_14 | 5.38% |
|  |  |  | VAIKVSKANKE | DRB1_01 | 2.02% |
|  |  |  | VAIKVSKANKE | DRB1_08 | 4.92% |
|  |  |  | VAIKVSKANKE | DRB1_11 | 2.57% |
|  |  |  | VAIKVSKANKE | DRB1_12 | 1.90% |
|  |  |  | VAIKVSKANKE | DRB1_13 | 0.00% |
|  |  |  | VAIKVSKANKE | DRB1_14 | 5.38% |
|  |  |  | KIPVAIKVSKA | DRB1_01 | 2.02% |
|  |  |  | KIPVAIKVSKA | DRB1_08 | 4.92% |
|  |  |  | KIPVAIKVSKA | DRB1_11 | 2.57% |
|  |  |  | KIPVAIKVSKA | DRB1_12 | 1.90% |
|  |  |  | KIPVAIKVSKA | DRB1_13 | 0.00% |
|  |  |  | KIPVAIKVSKA | DRB1_14 | 5.38% |
|  |  |  | IPVAIKVSKAN | DRB1_01 | 2.02% |
|  |  |  | IPVAIKVSKAN | DRB1_08 | 4.92% |
|  |  |  | IPVAIKVSKAN | DRB1_11 | 2.57% |
|  |  |  | IPVAIKVSKAN | DRB1_12 | 1.90% |
|  |  |  | IPVAIKVSKAN | DRB1_13 | 0.00% |
|  |  |  | IPVAIKVSKAN | DRB1_14 | 5.38% |
|  |  |  | VKIPVAIKVSK | DRB1_01 | 2.02% |
|  |  |  | VKIPVAIKVSK | DRB1_08 | 4.92% |
|  |  |  | VKIPVAIKVSK | DRB1_11 | 2.57% |
|  |  |  | VKIPVAIKVSK | DRB1_12 | 1.90% |
|  |  |  | VKIPVAIKVSK | DRB1_13 | 0.00% |
|  |  |  | VKIPVAIKVSK | DRB1_14 | 5.38% |
|  |  |  | PVAIKVSKANK | DRB1_01 | 0.00% |
|  |  |  | PVAIKVSKANK | DRB1_08 | 4.92% |
|  |  |  | PVAIKVSKANK | DRB1_11 | 2.57% |
|  |  |  | PVAIKVSKANK | DRB1_12 | 0.00% |
|  |  |  | PVAIKVSKANK | DRB1_13 | 0.00% |
|  |  |  | PVAIKVSKANK | DRB1_14 | 5.38% |
|  |  |  | IKVSKANKEILDEA | DRB1_01 | 0.00% |
|  |  |  | IKVSKANKEILDEA | DRB1_08 | 0.00% |
|  |  |  | IKVSKANKEILDEA | DRB1_11 | 0.00% |
|  |  |  | IKVSKANKEILDEA | DRB1_12 | 1.90% |
|  |  |  | IKVSKANKEILDEA | DRB1_13 | 0.00% |
|  |  |  | IKVSKANKEILDEA | DRB1_14 | 5.38% |
|  |  |  | IKVSKANKEILDE | DRB1_01 | 0.00% |
|  |  |  | IKVSKANKEILDE | DRB1_08 | 0.00% |
|  |  |  | IKVSKANKEILDE | DRB1_11 | 0.00% |
|  |  |  | IKVSKANKEILDE | DRB1_12 | 1.90% |
|  |  |  | IKVSKANKEILDE | DRB1_13 | 0.00% |
|  |  |  | IKVSKANKEILDE | DRB1_14 | 5.38% |
|  |  |  | IKVSKANKEILD | DRB1_01 | 0.00% |
|  |  |  | IKVSKANKEILD | DRB1_08 | 0.00% |
|  |  |  | IKVSKANKEILD | DRB1_11 | 0.00% |
|  |  |  | IKVSKANKEILD | DRB1_12 | 1.90% |
|  |  |  | IKVSKANKEILD | DRB1_13 | 0.00% |
|  |  |  | IKVSKANKEILD | DRB1_14 | 5.38% |
|  |  |  | IKVSKANKEIL | DRB1_01 | 0.00% |
|  |  |  | IKVSKANKEIL | DRB1_08 | 0.00% |
|  |  |  | IKVSKANKEIL | DRB1_11 | 0.00% |
|  |  |  | IKVSKANKEIL | DRB1_12 | 1.90% |
|  |  |  | IKVSKANKEIL | DRB1_13 | 0.00% |
|  |  |  | IKVSKANKEIL | DRB1_14 | 5.38% |
|  |  |  | VAIKVSKANK | DRB1_08 | 0.00% |
|  |  |  | VAIKVSKANK | DRB1_11 | 2.57% |
|  |  |  | VAIKVSKANK | DRB1_13 | 0.00% |
|  |  |  | VAIKVSKANK | DRB1_14 | 5.38% |
|  |  |  | AIKVSKANKE | DRB1_08 | 0.00% |
|  |  |  | AIKVSKANKE | DRB1_11 | 0.00% |
|  |  |  | AIKVSKANKE | DRB1_12 | 0.00% |
|  |  |  | AIKVSKANKE | DRB1_13 | 0.00% |
|  |  |  | AIKVSKANKE | DRB1_14 | 5.38% |
|  |  |  | IPVAIKVSKA | DRB1_08 | 1.40% |
|  |  |  | IPVAIKVSKA | DRB1_11 | 0.00% |
|  |  |  | IPVAIKVSKA | DRB1_12 | 0.00% |
|  |  |  | IPVAIKVSKA | DRB1_13 | 0.00% |
|  |  |  | IPVAIKVSKA | DRB1_14 | 5.38% |
|  |  |  | VKIPVAIKVS | DRB1_08 | 0.00% |
|  |  |  | VKIPVAIKVS | DRB1_12 | 0.00% |
|  |  |  | VKIPVAIKVS | DRB1_13 | 0.00% |
|  |  |  | VKIPVAIKVS | DRB1_14 | 0.00% |
|  |  |  | IKVSKANKEI | DRB1_08 | 0.00% |
|  |  |  | KVSKANKEILDE | DRB1_08 | 0.00% |
|  |  |  | KVSKANKEILDEA | DRB1_08 | 0.00% |
|  |  |  | KVSKANKEILDEAY | DRB1_08 | 0.00% |
|  |  |  | KVSKANKEILDE | DRB1_12 | 0.00% |
|  |  |  | KVSKANKEILDEA | DRB1_12 | 0.00% |
|  |  |  | KVSKANKEILDEAY | DRB1_12 | 0.00% |
|  |  |  | IKVSKANKEI | DRB1_13 | 0.00% |
|  |  |  | KVSKANKEILDE | DRB1_13 | 0.00% |
|  |  |  | KVSKANKEILDEA | DRB1_13 | 0.00% |
|  |  |  | KVSKANKEILDEAY | DRB1_13 | 0.00% |
|  |  |  | IKVSKANKEI | DRB1_14 | 0.00% |
|  |  |  | KVSKANKEILDE | DRB1_14 | 0.00% |
|  |  |  | KVSKANKEILDEA | DRB1_14 | 0.00% |
|  |  |  | KVSKANKEILDEAY | DRB1_14 | 0.00% |
|  |  |  | KIPVAIKVSK | DRB1_08 | 0.00% |
|  |  |  | KVSKANKEILD | DRB1_08 | 0.00% |
|  |  |  | KIPVAIKVSK | DRB1_11 | 0.00% |
|  |  |  | KVSKANKEILD | DRB1_12 | 0.00% |
|  |  |  | KIPVAIKVSK | DRB1_13 | 0.00% |
|  |  |  | KVSKANKEILD | DRB1_13 | 0.00% |
|  |  |  | KIPVAIKVSK | DRB1_14 | 0.00% |
|  |  |  | KVSKANKEILD | DRB1_14 | 0.00% |
|  |  |  | PVAIKVSKAN | DRB1_08 | 0.00% |
|  |  |  | PVAIKVSKAN | DRB1_13 | 0.00% |
|  |  |  | PVAIKVSKAN | DRB1_14 | 0.00% |
|  |  |  | KVSKANKEIL | DRB1_08 | 0.00% |
|  |  |  | KVSKANKEIL | DRB1_13 | 0.00% |
|  |  |  | AIKVSKANK | DRB1_08 | 0.00% |
|  |  |  | SKANKEILDEAYVM | DRB1_01 | 0.00% |
| Total |  | 54.83% |  |  | 81.21% |
