# Supplementary material for: Immunogenicity of Del19 EGFR mutations in Chinese patients affected by lung adenocarcinoma
Source: BMC Immunol. 2019 Nov 13;20:43. doi: 10.1186/s12865-019-0320-1 (PMC6854806; doi:10.1186/s12865-019-0320-1)
Supplement: Supplementary file 8 — Additional file 8. Predicted HLA binding epitopes for EGFR delL747_T751insP. [file 12865_2019_320_MOESM8_ESM.doc]

**Supplemental Table 8, Predicted HLA binding epitopes for EGFR delL747_T751insP by Chinese NSCLC patients as predicted by NetMHC4.0.** The percentages are the total frequencies of HLA alleles which may present a mutant EGFR.

| Class I | | | Class II | | |
| --- | --- | --- | --- | --- | --- |
| Neopeptide | HLA alleles | Frequency | Neopeptide | HLA alleles | Frequency |
| VAIKEPSPK | HLA-A*68 | 0.10% | GEKVKIPVAIKEPS | DRB1_01 | 2.02% |
| VAIKEPSPK | HLA-A*34 | 0.00% | GEKVKIPVAIKEPS | DRB1_08 | 4.92% |
| VAIKEPSPK | HLA-A*30 | 0.00% | GEKVKIPVAIKEPS | DRB1_11 | 2.57% |
| VAIKEPSPK | HLA-A*11 | 25.75% | GEKVKIPVAIKEPS | DRB1_12 | 1.90% |
| VAIKEPSPK | HLA-A*03 | 0.00% | GEKVKIPVAIKEPS | DRB1_13 | 0.00% |
| IPVAIKEPS | HLA-B*56 | 0.00% | GEKVKIPVAIKEPS | DRB1_14 | 5.38% |
| IPVAIKEPS | HLA-B*55 | 3.04% | EKVKIPVAIKEPS | DRB1_01 | 2.02% |
| IPVAIKEPS | HLA-B*54 | 3.16% | EKVKIPVAIKEPSP | DRB1_01 | 2.02% |
| AIKEPSPKA | HLA-A*30 | 7.56% | EKVKIPVAIKEPS | DRB1_08 | 3.69% |
| AIKEPSPKANK | HLA-A*30 | 0.00% | EKVKIPVAIKEPSP | DRB1_08 | 3.69% |
| AIKEPSPKANK | HLA-A*11 | 0.46% | EKVKIPVAIKEPS | DRB1_11 | 2.57% |
| AIKEPSPKANK | HLA-A*03 | 0.00% | EKVKIPVAIKEPSP | DRB1_11 | 2.57% |
|  |  |  | EKVKIPVAIKEPS | DRB1_12 | 1.90% |
|  |  |  | EKVKIPVAIKEPSP | DRB1_12 | 1.90% |
|  |  |  | EKVKIPVAIKEPS | DRB1_13 | 0.00% |
|  |  |  | EKVKIPVAIKEPSP | DRB1_13 | 0.00% |
|  |  |  | EKVKIPVAIKEPS | DRB1_14 | 5.38% |
|  |  |  | EKVKIPVAIKEPSP | DRB1_14 | 5.38% |
|  |  |  | KVKIPVAIKEPSPK | DRB1_08 | 3.69% |
|  |  |  | KVKIPVAIKEPSPK | DRB1_11 | 2.57% |
|  |  |  | KVKIPVAIKEPSPK | DRB1_12 | 1.90% |
|  |  |  | KVKIPVAIKEPSPK | DRB1_13 | 0.00% |
|  |  |  | KVKIPVAIKEPSPK | DRB1_14 | 5.38% |
|  |  |  | KVKIPVAIKEPSP | DRB1_08 | 2.29% |
|  |  |  | KVKIPVAIKEPSP | DRB1_11 | 2.57% |
|  |  |  | KVKIPVAIKEPSP | DRB1_12 | 0.00% |
|  |  |  | KVKIPVAIKEPSP | DRB1_13 | 0.00% |
|  |  |  | KVKIPVAIKEPSP | DRB1_14 | 5.38% |
|  |  |  | KVKIPVAIKEPS | DRB1_08 | 0.00% |
|  |  |  | KVKIPVAIKEPS | DRB1_11 | 2.57% |
|  |  |  | KVKIPVAIKEPS | DRB1_12 | 0.00% |
|  |  |  | KVKIPVAIKEPS | DRB1_13 | 0.00% |
|  |  |  | KVKIPVAIKEPS | DRB1_14 | 5.38% |
|  |  |  | IPVAIKEPSPKANK | DRB1_01 | 2.02% |
|  |  |  | IPVAIKEPSPKANK | DRB1_08 | 0.00% |
|  |  |  | IPVAIKEPSPKANK | DRB1_11 | 0.00% |
|  |  |  | IPVAIKEPSPKANK | DRB1_12 | 0.00% |
|  |  |  | IPVAIKEPSPKANK | DRB1_13 | 0.00% |
|  |  |  | IPVAIKEPSPKANK | DRB1_14 | 5.38% |
|  |  |  | KIPVAIKEPSPKAN | DRB1_01 | 0.00% |
|  |  |  | KIPVAIKEPSPKAN | DRB1_08 | 0.00% |
|  |  |  | KIPVAIKEPSPKAN | DRB1_11 | 0.00% |
|  |  |  | KIPVAIKEPSPKAN | DRB1_12 | 0.00% |
|  |  |  | KIPVAIKEPSPKAN | DRB1_13 | 0.00% |
|  |  |  | KIPVAIKEPSPKAN | DRB1_14 | 5.38% |
|  |  |  | VKIPVAIKEPSPKA | DRB1_08 | 0.00% |
|  |  |  | VKIPVAIKEPSPKA | DRB1_11 | 0.00% |
|  |  |  | VKIPVAIKEPSPKA | DRB1_12 | 0.00% |
|  |  |  | VKIPVAIKEPSPKA | DRB1_13 | 0.00% |
|  |  |  | VKIPVAIKEPSPKA | DRB1_14 | 5.38% |
|  |  |  | KIPVAIKEPSPKA | DRB1_08 | 0.00% |
|  |  |  | KIPVAIKEPSPKA | DRB1_11 | 0.00% |
|  |  |  | KIPVAIKEPSPKA | DRB1_12 | 0.00% |
|  |  |  | KIPVAIKEPSPKA | DRB1_13 | 0.00% |
|  |  |  | KIPVAIKEPSPKA | DRB1_14 | 3.71% |
|  |  |  | IPVAIKEPSPKAN | DRB1_01 | 0.00% |
|  |  |  | IPVAIKEPSPKAN | DRB1_08 | 0.00% |
|  |  |  | IPVAIKEPSPKAN | DRB1_11 | 0.00% |
|  |  |  | IPVAIKEPSPKAN | DRB1_12 | 0.00% |
|  |  |  | IPVAIKEPSPKAN | DRB1_13 | 0.00% |
|  |  |  | IPVAIKEPSPKAN | DRB1_14 | 0.00% |
|  |  |  | PVAIKEPSPKANK | DRB1_01 | 0.00% |
|  |  |  | PVAIKEPSPKANK | DRB1_08 | 0.00% |
|  |  |  | PVAIKEPSPKANK | DRB1_13 | 0.00% |
|  |  |  | PVAIKEPSPKANK | DRB1_14 | 0.00% |
|  |  |  | PVAIKEPSPKANKE | DRB1_01 | 0.00% |
|  |  |  | PVAIKEPSPKANKE | DRB1_08 | 0.00% |
|  |  |  | PVAIKEPSPKANKE | DRB1_13 | 0.00% |
|  |  |  | PVAIKEPSPKANKE | DRB1_14 | 0.00% |
|  |  |  | IPVAIKEPSPKA | DRB1_08 | 0.00% |
|  |  |  | PVAIKEPSPKAN | DRB1_08 | 0.00% |
|  |  |  | IPVAIKEPSPKA | DRB1_13 | 0.00% |
|  |  |  | PVAIKEPSPKAN | DRB1_13 | 0.00% |
|  |  |  | IPVAIKEPSPKA | DRB1_14 | 0.00% |
|  |  |  | PVAIKEPSPKAN | DRB1_14 | 0.00% |
|  |  |  | VKIPVAIKEPSPK | DRB1_08 | 0.00% |
|  |  |  | VKIPVAIKEPSPK | DRB1_13 | 0.00% |
|  |  |  | VKIPVAIKEPSPK | DRB1_14 | 0.00% |
|  |  |  | KIPVAIKEPSPK | DRB1_08 | 0.00% |
|  |  |  | VAIKEPSPKANK | DRB1_08 | 0.00% |
|  |  |  | VAIKEPSPKANKE | DRB1_08 | 0.00% |
|  |  |  | VAIKEPSPKANKEI | DRB1_08 | 0.00% |
|  |  |  | KIPVAIKEPSPK | DRB1_13 | 0.00% |
|  |  |  | VAIKEPSPKANK | DRB1_13 | 0.00% |
|  |  |  | VAIKEPSPKANKE | DRB1_13 | 0.00% |
|  |  |  | VAIKEPSPKANKEI | DRB1_13 | 0.00% |
|  |  |  | VKIPVAIKEPS | DRB1_08 | 0.00% |
|  |  |  | PVAIKEPSPKA | DRB1_08 | 0.00% |
|  |  |  | VKIPVAIKEPSP | DRB1_08 | 0.00% |
|  |  |  | IPVAIKEPSPK | DRB1_08 | 0.00% |
| Total |  | 39.61% |  |  | 16.79% |
