# Supplementary material for: Immunogenicity of Del19 EGFR mutations in Chinese patients affected by lung adenocarcinoma
Source: BMC Immunol. 2019 Nov 13;20:43. doi: 10.1186/s12865-019-0320-1 (PMC6854806; doi:10.1186/s12865-019-0320-1)
Supplement: Supplementary file 9 — Additional file 9. Predicted HLA binding epitopes for EGFR delE746_T751insA. [file 12865_2019_320_MOESM9_ESM.doc]

**Supplemental Table 9, Predicted HLA binding epitopes for EGFR delE746_T751insA by Chinese NSCLC patients as predicted by NetMHC4.0.** The percentages are the total frequencies of HLA alleles which may present a mutant EGFR.

| Class I | | | Class II | | |
| --- | --- | --- | --- | --- | --- |
| Neopeptide | HLA alleles | Frequency | Neopeptide | HLA alleles | Frequency |
| AIKASPKANK | HLA-A*03 | 3.56% | VAIKASPKANKEIL | DRB1_01 | 2.02% |
| AIKASPKANK | HLA-A*11 | 25.75% | VAIKASPKANKEIL | DRB1_03 | 5.95% |
| AIKASPKANK | HLA-A*30 | 7.56% | VAIKASPKANKEIL | DRB1_04 | 0.00% |
| AIKASPKANK | HLA-A*31 | 0.00% | VAIKASPKANKEIL | DRB1_08 | 16.05% |
| AIKASPKANK | HLA-A*34 | 0.00% | VAIKASPKANKEIL | DRB1_09 | 0.00% |
| AIKASPKANK | HLA-A*68 | 0.10% | VAIKASPKANKEIL | DRB1_11 | 8.11% |
| IPVAIKASPK | HLA-A*03 | 2.88% | VAIKASPKANKEIL | DRB1_12 | 1.90% |
| IPVAIKASPK | HLA-A*11 | 25.75% | VAIKASPKANKEIL | DRB1_13 | 0.00% |
| IPVAIKASPK | HLA-A*34 | 0.00% | VAIKASPKANKEIL | DRB1_14 | 19.14% |
| IPVAIKASPK | HLA-A*68 | 0.10% | VAIKASPKANKEIL | DRB1_16 | 0.00% |
| KIPVAIKASPK | HLA-A*03 | 2.88% | PVAIKASPKANKEI | DRB1_01 | 6.04% |
| KIPVAIKASPK | HLA-A*11 | 0.66% | PVAIKASPKANKEI | DRB1_03 | 0.00% |
| KIPVAIKASPK | HLA-A*30 | 0.00% | PVAIKASPKANKEI | DRB1_04 | 0.00% |
| KVKIPVAIKA | HLA-A*03 | 0.00% | PVAIKASPKANKEI | DRB1_08 | 16.05% |
| KVKIPVAIKA | HLA-A*30 | 7.56% | PVAIKASPKANKEI | DRB1_09 | 0.00% |
|  |  |  | PVAIKASPKANKEI | DRB1_11 | 8.11% |
|  |  |  | PVAIKASPKANKEI | DRB1_12 | 1.90% |
|  |  |  | PVAIKASPKANKEI | DRB1_13 | 0.00% |
|  |  |  | PVAIKASPKANKEI | DRB1_14 | 19.14% |
|  |  |  | PVAIKASPKANKEI | DRB1_16 | 0.00% |
|  |  |  | KIPVAIKASPKANK | DRB1_01 | 6.04% |
|  |  |  | KIPVAIKASPKANK | DRB1_03 | 0.00% |
|  |  |  | KIPVAIKASPKANK | DRB1_04 | 0.00% |
|  |  |  | KIPVAIKASPKANK | DRB1_07 | 0.00% |
|  |  |  | KIPVAIKASPKANK | DRB1_08 | 16.05% |
|  |  |  | KIPVAIKASPKANK | DRB1_09 | 0.00% |
|  |  |  | KIPVAIKASPKANK | DRB1_11 | 8.11% |
|  |  |  | KIPVAIKASPKANK | DRB1_12 | 1.90% |
|  |  |  | KIPVAIKASPKANK | DRB1_13 | 0.00% |
|  |  |  | KIPVAIKASPKANK | DRB1_14 | 19.14% |
|  |  |  | KIPVAIKASPKANK | DRB1_15 | 0.00% |
|  |  |  | KIPVAIKASPKANK | DRB1_16 | 6.81% |
|  |  |  | IPVAIKASPKANKE | DRB1_01 | 6.04% |
|  |  |  | IPVAIKASPKANKE | DRB1_03 | 0.00% |
|  |  |  | IPVAIKASPKANKE | DRB1_04 | 0.00% |
|  |  |  | IPVAIKASPKANKE | DRB1_08 | 16.05% |
|  |  |  | IPVAIKASPKANKE | DRB1_09 | 0.00% |
|  |  |  | IPVAIKASPKANKE | DRB1_11 | 8.11% |
|  |  |  | IPVAIKASPKANKE | DRB1_12 | 1.90% |
|  |  |  | IPVAIKASPKANKE | DRB1_13 | 0.00% |
|  |  |  | IPVAIKASPKANKE | DRB1_14 | 13.50% |
|  |  |  | IPVAIKASPKANKE | DRB1_15 | 0.00% |
|  |  |  | IPVAIKASPKANKE | DRB1_16 | 6.81% |
|  |  |  | IPVAIKASPKANK | DRB1_01 | 6.04% |
|  |  |  | IPVAIKASPKANK | DRB1_03 | 0.00% |
|  |  |  | IPVAIKASPKANK | DRB1_04 | 0.00% |
|  |  |  | IPVAIKASPKANK | DRB1_08 | 16.05% |
|  |  |  | IPVAIKASPKANK | DRB1_09 | 0.00% |
|  |  |  | IPVAIKASPKANK | DRB1_11 | 8.11% |
|  |  |  | IPVAIKASPKANK | DRB1_12 | 1.90% |
|  |  |  | IPVAIKASPKANK | DRB1_13 | 0.00% |
|  |  |  | IPVAIKASPKANK | DRB1_14 | 13.50% |
|  |  |  | IPVAIKASPKANK | DRB1_16 | 0.00% |
|  |  |  | KVKIPVAIKASPKA | DRB1_01 | 6.04% |
|  |  |  | VKIPVAIKASPKAN | DRB1_01 | 6.04% |
|  |  |  | KVKIPVAIKASPKA | DRB1_04 | 0.00% |
|  |  |  | VKIPVAIKASPKAN | DRB1_04 | 0.00% |
|  |  |  | KVKIPVAIKASPKA | DRB1_07 | 0.00% |
|  |  |  | KVKIPVAIKASPKA | DRB1_08 | 16.05% |
|  |  |  | VKIPVAIKASPKAN | DRB1_08 | 16.05% |
|  |  |  | KVKIPVAIKASPKA | DRB1_09 | 0.00% |
|  |  |  | VKIPVAIKASPKAN | DRB1_09 | 0.00% |
|  |  |  | KVKIPVAIKASPKA | DRB1_11 | 8.11% |
|  |  |  | VKIPVAIKASPKAN | DRB1_11 | 8.11% |
|  |  |  | KVKIPVAIKASPKA | DRB1_12 | 1.90% |
|  |  |  | VKIPVAIKASPKAN | DRB1_12 | 1.90% |
|  |  |  | KVKIPVAIKASPKA | DRB1_13 | 0.00% |
|  |  |  | VKIPVAIKASPKAN | DRB1_13 | 0.00% |
|  |  |  | KVKIPVAIKASPKA | DRB1_14 | 13.50% |
|  |  |  | VKIPVAIKASPKAN | DRB1_14 | 13.50% |
|  |  |  | KVKIPVAIKASPKA | DRB1_15 | 0.00% |
|  |  |  | VKIPVAIKASPKAN | DRB1_15 | 0.00% |
|  |  |  | KVKIPVAIKASPKA | DRB1_16 | 6.81% |
|  |  |  | VKIPVAIKASPKAN | DRB1_16 | 6.81% |
|  |  |  | PVAIKASPKANKE | DRB1_01 | 6.04% |
|  |  |  | PVAIKASPKANKE | DRB1_03 | 0.00% |
|  |  |  | PVAIKASPKANKE | DRB1_04 | 0.00% |
|  |  |  | PVAIKASPKANKE | DRB1_08 | 16.05% |
|  |  |  | PVAIKASPKANKE | DRB1_09 | 0.00% |
|  |  |  | PVAIKASPKANKE | DRB1_11 | 8.11% |
|  |  |  | PVAIKASPKANKE | DRB1_12 | 1.90% |
|  |  |  | PVAIKASPKANKE | DRB1_13 | 0.00% |
|  |  |  | PVAIKASPKANKE | DRB1_14 | 13.50% |
|  |  |  | PVAIKASPKANKE | DRB1_16 | 0.00% |
|  |  |  | KIPVAIKASPKAN | DRB1_01 | 6.04% |
|  |  |  | KIPVAIKASPKAN | DRB1_04 | 0.00% |
|  |  |  | KIPVAIKASPKAN | DRB1_08 | 16.05% |
|  |  |  | KIPVAIKASPKAN | DRB1_09 | 0.00% |
|  |  |  | KIPVAIKASPKAN | DRB1_11 | 8.11% |
|  |  |  | KIPVAIKASPKAN | DRB1_12 | 1.90% |
|  |  |  | KIPVAIKASPKAN | DRB1_13 | 0.00% |
|  |  |  | KIPVAIKASPKAN | DRB1_14 | 13.50% |
|  |  |  | KIPVAIKASPKAN | DRB1_16 | 0.00% |
|  |  |  | VAIKASPKANKEI | DRB1_01 | 2.02% |
|  |  |  | VAIKASPKANKEI | DRB1_03 | 0.00% |
|  |  |  | VAIKASPKANKEI | DRB1_04 | 0.00% |
|  |  |  | VAIKASPKANKEI | DRB1_08 | 4.92% |
|  |  |  | VAIKASPKANKEI | DRB1_09 | 0.00% |
|  |  |  | VAIKASPKANKEI | DRB1_11 | 8.11% |
|  |  |  | VAIKASPKANKEI | DRB1_12 | 1.90% |
|  |  |  | VAIKASPKANKEI | DRB1_13 | 0.00% |
|  |  |  | VAIKASPKANKEI | DRB1_14 | 13.50% |
|  |  |  | VAIKASPKANKEI | DRB1_16 | 0.00% |
|  |  |  | EKVKIPVAIKASPK | DRB1_01 | 6.04% |
|  |  |  | EKVKIPVAIKASPK | DRB1_08 | 4.92% |
|  |  |  | EKVKIPVAIKASPK | DRB1_09 | 0.00% |
|  |  |  | EKVKIPVAIKASPK | DRB1_11 | 8.11% |
|  |  |  | EKVKIPVAIKASPK | DRB1_12 | 1.90% |
|  |  |  | EKVKIPVAIKASPK | DRB1_13 | 0.00% |
|  |  |  | EKVKIPVAIKASPK | DRB1_14 | 13.50% |
|  |  |  | EKVKIPVAIKASPK | DRB1_15 | 0.00% |
|  |  |  | EKVKIPVAIKASPK | DRB1_16 | 0.00% |
|  |  |  | GEKVKIPVAIKASP | DRB1_01 | 6.04% |
|  |  |  | GEKVKIPVAIKASP | DRB1_08 | 4.92% |
|  |  |  | GEKVKIPVAIKASP | DRB1_09 | 0.00% |
|  |  |  | GEKVKIPVAIKASP | DRB1_11 | 8.11% |
|  |  |  | GEKVKIPVAIKASP | DRB1_12 | 1.90% |
|  |  |  | GEKVKIPVAIKASP | DRB1_13 | 0.00% |
|  |  |  | GEKVKIPVAIKASP | DRB1_14 | 13.50% |
|  |  |  | GEKVKIPVAIKASP | DRB1_15 | 0.00% |
|  |  |  | GEKVKIPVAIKASP | DRB1_16 | 0.00% |
|  |  |  | GEKVKIPVAIKAS | DRB1_01 | 6.04% |
|  |  |  | EGEKVKIPVAIKAS | DRB1_01 | 6.04% |
|  |  |  | GEKVKIPVAIKAS | DRB1_08 | 4.92% |
|  |  |  | EGEKVKIPVAIKAS | DRB1_08 | 4.92% |
|  |  |  | GEKVKIPVAIKAS | DRB1_09 | 0.00% |
|  |  |  | EGEKVKIPVAIKAS | DRB1_09 | 0.00% |
|  |  |  | GEKVKIPVAIKAS | DRB1_11 | 8.11% |
|  |  |  | EGEKVKIPVAIKAS | DRB1_11 | 8.11% |
|  |  |  | GEKVKIPVAIKAS | DRB1_12 | 1.90% |
|  |  |  | EGEKVKIPVAIKAS | DRB1_12 | 1.90% |
|  |  |  | GEKVKIPVAIKAS | DRB1_13 | 0.00% |
|  |  |  | EGEKVKIPVAIKAS | DRB1_13 | 0.00% |
|  |  |  | GEKVKIPVAIKAS | DRB1_14 | 13.50% |
|  |  |  | EGEKVKIPVAIKAS | DRB1_14 | 13.50% |
|  |  |  | GEKVKIPVAIKAS | DRB1_15 | 0.00% |
|  |  |  | EGEKVKIPVAIKAS | DRB1_15 | 0.00% |
|  |  |  | GEKVKIPVAIKAS | DRB1_16 | 0.00% |
|  |  |  | EGEKVKIPVAIKAS | DRB1_16 | 0.00% |
|  |  |  | EKVKIPVAIKASP | DRB1_01 | 6.04% |
|  |  |  | EKVKIPVAIKASP | DRB1_08 | 4.92% |
|  |  |  | EKVKIPVAIKASP | DRB1_09 | 0.00% |
|  |  |  | EKVKIPVAIKASP | DRB1_11 | 8.11% |
|  |  |  | EKVKIPVAIKASP | DRB1_12 | 1.90% |
|  |  |  | EKVKIPVAIKASP | DRB1_13 | 0.00% |
|  |  |  | EKVKIPVAIKASP | DRB1_14 | 13.50% |
|  |  |  | EKVKIPVAIKASP | DRB1_16 | 0.00% |
|  |  |  | PVAIKASPKANK | DRB1_01 | 2.02% |
|  |  |  | PVAIKASPKANK | DRB1_08 | 4.92% |
|  |  |  | PVAIKASPKANK | DRB1_09 | 0.00% |
|  |  |  | PVAIKASPKANK | DRB1_11 | 8.11% |
|  |  |  | PVAIKASPKANK | DRB1_12 | 1.90% |
|  |  |  | PVAIKASPKANK | DRB1_13 | 0.00% |
|  |  |  | PVAIKASPKANK | DRB1_14 | 13.50% |
|  |  |  | EKVKIPVAIKAS | DRB1_01 | 6.04% |
|  |  |  | EKVKIPVAIKAS | DRB1_08 | 4.92% |
|  |  |  | EKVKIPVAIKAS | DRB1_09 | 0.00% |
|  |  |  | EKVKIPVAIKAS | DRB1_11 | 8.11% |
|  |  |  | EKVKIPVAIKAS | DRB1_12 | 1.90% |
|  |  |  | EKVKIPVAIKAS | DRB1_13 | 0.00% |
|  |  |  | EKVKIPVAIKAS | DRB1_14 | 13.50% |
|  |  |  | EKVKIPVAIKAS | DRB1_16 | 0.00% |
|  |  |  | KVKIPVAIKASPK | DRB1_01 | 6.04% |
|  |  |  | VKIPVAIKASPKA | DRB1_01 | 6.04% |
|  |  |  | KVKIPVAIKASPK | DRB1_08 | 4.92% |
|  |  |  | VKIPVAIKASPKA | DRB1_08 | 4.92% |
|  |  |  | KVKIPVAIKASPK | DRB1_09 | 0.00% |
|  |  |  | VKIPVAIKASPKA | DRB1_09 | 0.00% |
|  |  |  | KVKIPVAIKASPK | DRB1_11 | 8.11% |
|  |  |  | VKIPVAIKASPKA | DRB1_11 | 8.11% |
|  |  |  | KVKIPVAIKASPK | DRB1_12 | 1.90% |
|  |  |  | VKIPVAIKASPKA | DRB1_12 | 1.90% |
|  |  |  | KVKIPVAIKASPK | DRB1_13 | 0.00% |
|  |  |  | VKIPVAIKASPKA | DRB1_13 | 0.00% |
|  |  |  | KVKIPVAIKASPK | DRB1_14 | 13.50% |
|  |  |  | VKIPVAIKASPKA | DRB1_14 | 13.50% |
|  |  |  | KVKIPVAIKASPK | DRB1_16 | 0.00% |
|  |  |  | VKIPVAIKASPKA | DRB1_16 | 0.00% |
|  |  |  | AIKASPKANKEILD | DRB1_01 | 2.02% |
|  |  |  | AIKASPKANKEILD | DRB1_03 | 0.00% |
|  |  |  | AIKASPKANKEILD | DRB1_08 | 4.92% |
|  |  |  | AIKASPKANKEILD | DRB1_09 | 0.00% |
|  |  |  | AIKASPKANKEILD | DRB1_11 | 8.11% |
|  |  |  | AIKASPKANKEILD | DRB1_12 | 1.90% |
|  |  |  | AIKASPKANKEILD | DRB1_13 | 0.00% |
|  |  |  | AIKASPKANKEILD | DRB1_14 | 13.50% |
|  |  |  | AIKASPKANKEIL | DRB1_01 | 2.02% |
|  |  |  | AIKASPKANKEIL | DRB1_03 | 0.00% |
|  |  |  | AIKASPKANKEIL | DRB1_08 | 4.92% |
|  |  |  | AIKASPKANKEIL | DRB1_09 | 0.00% |
|  |  |  | AIKASPKANKEIL | DRB1_11 | 8.11% |
|  |  |  | AIKASPKANKEIL | DRB1_12 | 1.90% |
|  |  |  | AIKASPKANKEIL | DRB1_13 | 0.00% |
|  |  |  | AIKASPKANKEIL | DRB1_14 | 13.50% |
|  |  |  | VAIKASPKANKE | DRB1_01 | 2.02% |
|  |  |  | VAIKASPKANKE | DRB1_03 | 0.00% |
|  |  |  | VAIKASPKANKE | DRB1_08 | 4.92% |
|  |  |  | VAIKASPKANKE | DRB1_09 | 0.00% |
|  |  |  | VAIKASPKANKE | DRB1_11 | 8.11% |
|  |  |  | VAIKASPKANKE | DRB1_12 | 1.90% |
|  |  |  | VAIKASPKANKE | DRB1_13 | 0.00% |
|  |  |  | VAIKASPKANKE | DRB1_14 | 13.50% |
|  |  |  | IPVAIKASPKAN | DRB1_01 | 6.04% |
|  |  |  | IPVAIKASPKAN | DRB1_08 | 4.92% |
|  |  |  | IPVAIKASPKAN | DRB1_09 | 0.00% |
|  |  |  | IPVAIKASPKAN | DRB1_11 | 2.57% |
|  |  |  | IPVAIKASPKAN | DRB1_12 | 1.90% |
|  |  |  | IPVAIKASPKAN | DRB1_13 | 0.00% |
|  |  |  | IPVAIKASPKAN | DRB1_14 | 5.38% |
|  |  |  | KIPVAIKASPKA | DRB1_01 | 2.02% |
|  |  |  | KIPVAIKASPKA | DRB1_08 | 4.92% |
|  |  |  | KIPVAIKASPKA | DRB1_09 | 0.00% |
|  |  |  | KIPVAIKASPKA | DRB1_11 | 2.57% |
|  |  |  | KIPVAIKASPKA | DRB1_12 | 1.90% |
|  |  |  | KIPVAIKASPKA | DRB1_13 | 0.00% |
|  |  |  | KIPVAIKASPKA | DRB1_14 | 5.38% |
|  |  |  | KVKIPVAIKASP | DRB1_01 | 2.02% |
|  |  |  | KVKIPVAIKASP | DRB1_08 | 4.92% |
|  |  |  | KVKIPVAIKASP | DRB1_09 | 0.00% |
|  |  |  | KVKIPVAIKASP | DRB1_11 | 2.57% |
|  |  |  | KVKIPVAIKASP | DRB1_12 | 1.90% |
|  |  |  | KVKIPVAIKASP | DRB1_13 | 0.00% |
|  |  |  | KVKIPVAIKASP | DRB1_14 | 5.38% |
|  |  |  | KVKIPVAIKAS | DRB1_01 | 2.02% |
|  |  |  | KVKIPVAIKAS | DRB1_08 | 4.92% |
|  |  |  | KVKIPVAIKAS | DRB1_11 | 2.57% |
|  |  |  | KVKIPVAIKAS | DRB1_12 | 1.90% |
|  |  |  | KVKIPVAIKAS | DRB1_13 | 0.00% |
|  |  |  | KVKIPVAIKAS | DRB1_14 | 5.38% |
|  |  |  | VAIKASPKANK | DRB1_01 | 2.02% |
|  |  |  | VAIKASPKANK | DRB1_08 | 4.92% |
|  |  |  | VAIKASPKANK | DRB1_11 | 2.57% |
|  |  |  | VAIKASPKANK | DRB1_12 | 0.00% |
|  |  |  | VAIKASPKANK | DRB1_13 | 0.00% |
|  |  |  | VAIKASPKANK | DRB1_14 | 5.38% |
|  |  |  | AIKASPKANKEI | DRB1_01 | 0.00% |
|  |  |  | AIKASPKANKEI | DRB1_03 | 0.00% |
|  |  |  | AIKASPKANKEI | DRB1_08 | 4.92% |
|  |  |  | AIKASPKANKEI | DRB1_11 | 2.57% |
|  |  |  | AIKASPKANKEI | DRB1_12 | 0.00% |
|  |  |  | AIKASPKANKEI | DRB1_13 | 0.00% |
|  |  |  | AIKASPKANKEI | DRB1_14 | 12.37% |
|  |  |  | VKIPVAIKASPK | DRB1_01 | 2.02% |
|  |  |  | VKIPVAIKASPK | DRB1_08 | 4.92% |
|  |  |  | VKIPVAIKASPK | DRB1_11 | 2.57% |
|  |  |  | VKIPVAIKASPK | DRB1_12 | 0.00% |
|  |  |  | VKIPVAIKASPK | DRB1_13 | 0.00% |
|  |  |  | VKIPVAIKASPK | DRB1_14 | 5.38% |
|  |  |  | IPVAIKASPKA | DRB1_01 | 2.02% |
|  |  |  | IPVAIKASPKA | DRB1_08 | 4.92% |
|  |  |  | IPVAIKASPKA | DRB1_11 | 2.57% |
|  |  |  | IPVAIKASPKA | DRB1_12 | 0.00% |
|  |  |  | IPVAIKASPKA | DRB1_13 | 0.00% |
|  |  |  | IPVAIKASPKA | DRB1_14 | 5.38% |
|  |  |  | PVAIKASPKAN | DRB1_01 | 2.02% |
|  |  |  | PVAIKASPKAN | DRB1_08 | 4.92% |
|  |  |  | PVAIKASPKAN | DRB1_11 | 2.57% |
|  |  |  | PVAIKASPKAN | DRB1_12 | 0.00% |
|  |  |  | PVAIKASPKAN | DRB1_13 | 0.00% |
|  |  |  | PVAIKASPKAN | DRB1_14 | 5.38% |
|  |  |  | AIKASPKANKE | DRB1_08 | 1.23% |
|  |  |  | AIKASPKANKE | DRB1_11 | 2.57% |
|  |  |  | AIKASPKANKE | DRB1_13 | 0.00% |
|  |  |  | AIKASPKANKE | DRB1_14 | 5.38% |
|  |  |  | IKASPKANKEILD | DRB1_08 | 0.00% |
|  |  |  | IKASPKANKEILDE | DRB1_08 | 0.00% |
|  |  |  | IKASPKANKEILD | DRB1_11 | 2.57% |
|  |  |  | IKASPKANKEILDE | DRB1_11 | 2.57% |
|  |  |  | IKASPKANKEILD | DRB1_13 | 0.00% |
|  |  |  | IKASPKANKEILDE | DRB1_13 | 0.00% |
|  |  |  | IKASPKANKEILD | DRB1_14 | 5.38% |
|  |  |  | IKASPKANKEILDE | DRB1_14 | 5.38% |
|  |  |  | IKASPKANKEIL | DRB1_08 | 0.00% |
|  |  |  | IKASPKANKEIL | DRB1_11 | 0.00% |
|  |  |  | IKASPKANKEIL | DRB1_13 | 0.00% |
|  |  |  | IKASPKANKEIL | DRB1_14 | 5.38% |
|  |  |  | AIKASPKANK | DRB1_08 | 0.00% |
|  |  |  | AIKASPKANK | DRB1_11 | 0.00% |
|  |  |  | AIKASPKANK | DRB1_13 | 0.00% |
|  |  |  | AIKASPKANK | DRB1_14 | 3.71% |
|  |  |  | KIPVAIKASPK | DRB1_08 | 0.00% |
|  |  |  | KIPVAIKASPK | DRB1_11 | 0.00% |
|  |  |  | KIPVAIKASPK | DRB1_13 | 0.00% |
|  |  |  | KIPVAIKASPK | DRB1_14 | 0.00% |
|  |  |  | VKIPVAIKASP | DRB1_08 | 0.00% |
|  |  |  | VKIPVAIKASP | DRB1_12 | 0.00% |
|  |  |  | VKIPVAIKASP | DRB1_13 | 0.00% |
|  |  |  | VKIPVAIKASP | DRB1_14 | 0.00% |
|  |  |  | PVAIKASPKA | DRB1_08 | 0.00% |
|  |  |  | PVAIKASPKA | DRB1_13 | 0.00% |
|  |  |  | PVAIKASPKA | DRB1_14 | 0.00% |
|  |  |  | VAIKASPKAN | DRB1_08 | 0.00% |
|  |  |  | VAIKASPKAN | DRB1_13 | 0.00% |
|  |  |  | VAIKASPKAN | DRB1_14 | 0.00% |
|  |  |  | VKIPVAIKAS | DRB1_08 | 0.00% |
|  |  |  | VKIPVAIKAS | DRB1_13 | 0.00% |
|  |  |  | VKIPVAIKAS | DRB1_14 | 0.00% |
|  |  |  | IKASPKANKEI | DRB1_08 | 0.00% |
|  |  |  | IKASPKANKEI | DRB1_13 | 0.00% |
|  |  |  | IKASPKANKEI | DRB1_14 | 0.00% |
|  |  |  | KIPVAIKASP | DRB1_08 | 0.00% |
|  |  |  | IPVAIKASPK | DRB1_08 | 0.00% |
|  |  |  | VAIKASPKA | DRB1_08 | 0.00% |
|  |  |  | IKASPKANKE | DRB1_08 | 0.00% |
| Total |  | 36.97% |  |  | 64.00% |
