# Supplementary material for: Immunogenicity of Del19 EGFR mutations in Chinese patients affected by lung adenocarcinoma
Source: BMC Immunol. 2019 Nov 13;20:43. doi: 10.1186/s12865-019-0320-1 (PMC6854806; doi:10.1186/s12865-019-0320-1)
Supplement: Supplementary file 10 — Additional file 10. Predicted HLA binding epitopes for EGFR delL747_P753. [file 12865_2019_320_MOESM10_ESM.doc]

**Supplemental Table 10, Predicted HLA binding epitopes for EGFR delL747_P753 by Chinese NSCLC patients as predicted by NetMHC4.0.** The percentages are the total frequencies of HLA alleles which may present a mutant EGFR.

| Class I | | | Class II | | |
| --- | --- | --- | --- | --- | --- |
| Neopeptide | HLA alleles | Frequency | Neopeptide | HLA alleles | Frequency |
| KVKIPVAIKEK | HLA-A*03 | 2.88% | GEKVKIPVAIKEKA | DRB1_01 | 2.02% |
| KVKIPVAIKEK | HLA-A*11 | 0.46% | GEKVKIPVAIKEKA | DRB1_08 | 4.92% |
| KVKIPVAIKEK | HLA-A*30 | 7.56% | GEKVKIPVAIKEKA | DRB1_09 | 0.00% |
| KVKIPVAIKEK | HLA-A*31 | 0.00% | GEKVKIPVAIKEKA | DRB1_11 | 8.11% |
| KIPVAIKEK | HLA-A*03 | 0.00% | GEKVKIPVAIKEKA | DRB1_12 | 1.90% |
| KIPVAIKEK | HLA-A*11 | 24.34% | GEKVKIPVAIKEKA | DRB1_13 | 0.00% |
| KIPVAIKEK | HLA-A*30 | 7.56% | GEKVKIPVAIKEKA | DRB1_14 | 13.50% |
| KIPVAIKEK | HLA-A*68 | 0.10% | GEKVKIPVAIKEKA | DRB1_16 | 0.00% |
| KEKANKEIL | HLA-B*40 | 4.13% | EKVKIPVAIKEKAN | DRB1_01 | 2.02% |
| KEKANKEIL | HLA-B*404 | 0.00% | EKVKIPVAIKEKAN | DRB1_08 | 4.92% |
| KEKANKEIL | HLA-B*407 | 0.00% | EKVKIPVAIKEKAN | DRB1_11 | 8.11% |
| KEKANKEIL | HLA-B*401 | 0.00% | EKVKIPVAIKEKAN | DRB1_12 | 1.90% |
| KEKANKEIL | HLA-B*409 | 0.00% | EKVKIPVAIKEKAN | DRB1_13 | 0.00% |
| KEKANKEIL | HLA-B*400 | 0.00% | EKVKIPVAIKEKAN | DRB1_14 | 13.50% |
| KEKANKEIL | HLA-B*402 | 0.00% | EKVKIPVAIKEKA | DRB1_01 | 2.02% |
| KEKANKEIL | HLA-B*406 | 0.00% | EKVKIPVAIKEKA | DRB1_08 | 4.92% |
| KEKANKEIL | HLA-B*405 | 0.00% | EKVKIPVAIKEKA | DRB1_11 | 8.11% |
| KEKANKEIL | HLA-B*41 | 0.09% | EKVKIPVAIKEKA | DRB1_12 | 1.90% |
| IPVAIKEKA | HLA-B*54 | 3.16% | EKVKIPVAIKEKA | DRB1_13 | 0.00% |
| IPVAIKEKA | HLA-B*55 | 3.15% | EKVKIPVAIKEKA | DRB1_14 | 13.50% |
| IPVAIKEKA | HLA-B*56 | 0.84% | KVKIPVAIKEKANK | DRB1_01 | 2.02% |
| VAIKEKANK | HLA-A*68 | 0.10% | KVKIPVAIKEKANK | DRB1_08 | 4.92% |
|  |  |  | KVKIPVAIKEKANK | DRB1_11 | 2.57% |
|  |  |  | KVKIPVAIKEKANK | DRB1_12 | 1.90% |
|  |  |  | KVKIPVAIKEKANK | DRB1_13 | 0.00% |
|  |  |  | KVKIPVAIKEKANK | DRB1_14 | 12.37% |
|  |  |  | KVKIPVAIKEKAN | DRB1_01 | 2.02% |
|  |  |  | KVKIPVAIKEKAN | DRB1_08 | 4.92% |
|  |  |  | KVKIPVAIKEKAN | DRB1_11 | 2.57% |
|  |  |  | KVKIPVAIKEKAN | DRB1_12 | 1.90% |
|  |  |  | KVKIPVAIKEKAN | DRB1_13 | 0.00% |
|  |  |  | KVKIPVAIKEKAN | DRB1_14 | 12.37% |
|  |  |  | KVKIPVAIKEKA | DRB1_01 | 0.00% |
|  |  |  | KVKIPVAIKEKA | DRB1_08 | 4.92% |
|  |  |  | KVKIPVAIKEKA | DRB1_11 | 2.57% |
|  |  |  | KVKIPVAIKEKA | DRB1_12 | 1.90% |
|  |  |  | KVKIPVAIKEKA | DRB1_13 | 0.00% |
|  |  |  | KVKIPVAIKEKA | DRB1_14 | 5.38% |
|  |  |  | IPVAIKEKANKEIL | DRB1_01 | 0.00% |
|  |  |  | IPVAIKEKANKEIL | DRB1_08 | 0.00% |
|  |  |  | IPVAIKEKANKEIL | DRB1_11 | 2.57% |
|  |  |  | IPVAIKEKANKEIL | DRB1_12 | 1.90% |
|  |  |  | IPVAIKEKANKEIL | DRB1_13 | 0.00% |
|  |  |  | IPVAIKEKANKEIL | DRB1_14 | 5.38% |
|  |  |  | KIPVAIKEKANKEI | DRB1_08 | 0.00% |
|  |  |  | KIPVAIKEKANKEI | DRB1_11 | 2.57% |
|  |  |  | KIPVAIKEKANKEI | DRB1_12 | 1.90% |
|  |  |  | KIPVAIKEKANKEI | DRB1_13 | 0.00% |
|  |  |  | KIPVAIKEKANKEI | DRB1_14 | 5.38% |
|  |  |  | VKIPVAIKEKANKE | DRB1_08 | 0.00% |
|  |  |  | VKIPVAIKEKANKE | DRB1_11 | 2.57% |
|  |  |  | VKIPVAIKEKANKE | DRB1_12 | 1.90% |
|  |  |  | VKIPVAIKEKANKE | DRB1_13 | 0.00% |
|  |  |  | VKIPVAIKEKANKE | DRB1_14 | 5.38% |
|  |  |  | VKIPVAIKEKANK | DRB1_08 | 0.00% |
|  |  |  | VKIPVAIKEKANK | DRB1_11 | 2.57% |
|  |  |  | VKIPVAIKEKANK | DRB1_12 | 1.90% |
|  |  |  | VKIPVAIKEKANK | DRB1_13 | 0.00% |
|  |  |  | VKIPVAIKEKANK | DRB1_14 | 5.38% |
|  |  |  | KIPVAIKEKANKE | DRB1_08 | 0.00% |
|  |  |  | KIPVAIKEKANKE | DRB1_11 | 2.57% |
|  |  |  | KIPVAIKEKANKE | DRB1_12 | 0.00% |
|  |  |  | KIPVAIKEKANKE | DRB1_13 | 0.00% |
|  |  |  | KIPVAIKEKANKE | DRB1_14 | 5.38% |
|  |  |  | IPVAIKEKANKEI | DRB1_08 | 0.00% |
|  |  |  | IPVAIKEKANKEI | DRB1_11 | 2.57% |
|  |  |  | IPVAIKEKANKEI | DRB1_12 | 0.00% |
|  |  |  | IPVAIKEKANKEI | DRB1_13 | 0.00% |
|  |  |  | IPVAIKEKANKEI | DRB1_14 | 5.38% |
|  |  |  | PVAIKEKANKEIL | DRB1_01 | 0.00% |
|  |  |  | PVAIKEKANKEIL | DRB1_08 | 0.00% |
|  |  |  | PVAIKEKANKEIL | DRB1_11 | 2.57% |
|  |  |  | PVAIKEKANKEIL | DRB1_12 | 0.00% |
|  |  |  | PVAIKEKANKEIL | DRB1_13 | 0.00% |
|  |  |  | PVAIKEKANKEIL | DRB1_14 | 5.38% |
|  |  |  | KIPVAIKEKANK | DRB1_08 | 0.00% |
|  |  |  | KIPVAIKEKANK | DRB1_11 | 2.57% |
|  |  |  | KIPVAIKEKANK | DRB1_12 | 0.00% |
|  |  |  | KIPVAIKEKANK | DRB1_13 | 0.00% |
|  |  |  | KIPVAIKEKANK | DRB1_14 | 5.38% |
|  |  |  | PVAIKEKANKEILD | DRB1_08 | 0.00% |
|  |  |  | PVAIKEKANKEILD | DRB1_11 | 2.57% |
|  |  |  | PVAIKEKANKEILD | DRB1_12 | 0.00% |
|  |  |  | PVAIKEKANKEILD | DRB1_13 | 0.00% |
|  |  |  | PVAIKEKANKEILD | DRB1_14 | 5.38% |
|  |  |  | VKIPVAIKEKAN | DRB1_08 | 0.00% |
|  |  |  | VKIPVAIKEKAN | DRB1_11 | 2.57% |
|  |  |  | VKIPVAIKEKAN | DRB1_12 | 0.00% |
|  |  |  | VKIPVAIKEKAN | DRB1_13 | 0.00% |
|  |  |  | VKIPVAIKEKAN | DRB1_14 | 5.38% |
|  |  |  | VAIKEKANKEIL | DRB1_08 | 0.00% |
|  |  |  | VAIKEKANKEIL | DRB1_11 | 0.00% |
|  |  |  | VAIKEKANKEIL | DRB1_12 | 0.00% |
|  |  |  | VAIKEKANKEIL | DRB1_13 | 0.00% |
|  |  |  | VAIKEKANKEIL | DRB1_14 | 5.38% |
|  |  |  | VAIKEKANKEILD | DRB1_08 | 0.00% |
|  |  |  | VAIKEKANKEILD | DRB1_11 | 0.00% |
|  |  |  | VAIKEKANKEILD | DRB1_12 | 0.00% |
|  |  |  | VAIKEKANKEILD | DRB1_13 | 0.00% |
|  |  |  | VAIKEKANKEILD | DRB1_14 | 5.38% |
|  |  |  | VAIKEKANKEILDE | DRB1_08 | 0.00% |
|  |  |  | VAIKEKANKEILDE | DRB1_11 | 0.00% |
|  |  |  | VAIKEKANKEILDE | DRB1_12 | 0.00% |
|  |  |  | VAIKEKANKEILDE | DRB1_13 | 0.00% |
|  |  |  | VAIKEKANKEILDE | DRB1_14 | 1.67% |
|  |  |  | VKIPVAIKEKA | DRB1_08 | 0.00% |
|  |  |  | VKIPVAIKEKA | DRB1_11 | 0.00% |
|  |  |  | VKIPVAIKEKA | DRB1_12 | 0.00% |
|  |  |  | VKIPVAIKEKA | DRB1_13 | 0.00% |
|  |  |  | VKIPVAIKEKA | DRB1_14 | 1.67% |
|  |  |  | PVAIKEKANKEI | DRB1_08 | 0.00% |
|  |  |  | PVAIKEKANKEI | DRB1_11 | 0.00% |
|  |  |  | PVAIKEKANKEI | DRB1_12 | 0.00% |
|  |  |  | PVAIKEKANKEI | DRB1_13 | 0.00% |
|  |  |  | PVAIKEKANKEI | DRB1_14 | 0.00% |
|  |  |  | IPVAIKEKANKE | DRB1_08 | 0.00% |
|  |  |  | IPVAIKEKANKE | DRB1_11 | 0.00% |
|  |  |  | IPVAIKEKANKE | DRB1_12 | 0.00% |
|  |  |  | IPVAIKEKANKE | DRB1_13 | 0.00% |
|  |  |  | IPVAIKEKANKE | DRB1_14 | 0.00% |
|  |  |  | KIPVAIKEKAN | DRB1_08 | 0.00% |
|  |  |  | KIPVAIKEKAN | DRB1_12 | 0.00% |
|  |  |  | KIPVAIKEKAN | DRB1_13 | 0.00% |
|  |  |  | KIPVAIKEKAN | DRB1_14 | 0.00% |
|  |  |  | IPVAIKEKANK | DRB1_08 | 0.00% |
|  |  |  | VAIKEKANKEI | DRB1_08 | 0.00% |
|  |  |  | IPVAIKEKANK | DRB1_13 | 0.00% |
|  |  |  | VAIKEKANKEI | DRB1_13 | 0.00% |
|  |  |  | IPVAIKEKANK | DRB1_14 | 0.00% |
|  |  |  | AIKEKANKEIL | DRB1_08 | 0.00% |
|  |  |  | AIKEKANKEIL | DRB1_13 | 0.00% |
|  |  |  | AIKEKANKEILD | DRB1_08 | 0.00% |
|  |  |  | AIKEKANKEILDE | DRB1_08 | 0.00% |
|  |  |  | AIKEKANKEILDEA | DRB1_08 | 0.00% |
|  |  |  | AIKEKANKEILD | DRB1_13 | 0.00% |
|  |  |  | AIKEKANKEILDE | DRB1_13 | 0.00% |
|  |  |  | AIKEKANKEILDEA | DRB1_13 | 0.00% |
|  |  |  | KIPVAIKEKA | DRB1_08 | 0.00% |
|  |  |  | PVAIKEKANKE | DRB1_08 | 0.00% |
|  |  |  | EKANKEILDEAYVM | DRB1_01 | 0.00% |
|  |  |  | IPVAIKEKAN | DRB1_08 | 0.00% |
| Total |  | 46.25% |  |  | 30.46% |
