# Supplementary material for: Immunogenicity of Del19 EGFR mutations in Chinese patients affected by lung adenocarcinoma
Source: BMC Immunol. 2019 Nov 13;20:43. doi: 10.1186/s12865-019-0320-1 (PMC6854806; doi:10.1186/s12865-019-0320-1)
Supplement: Supplementary file 11 — Additional file 11. Predicted HLA binding epitopes for EGFR delS752_I759. [file 12865_2019_320_MOESM11_ESM.doc]

**Supplemental Table 11, Predicted HLA binding epitopes for EGFR delS752_I759 by Chinese NSCLC patients as predicted by NetMHC4.0.** The percentages are the total frequencies of HLA alleles which may present a mutant EGFR.

| Class I | | | Class II | | |
| --- | --- | --- | --- | --- | --- |
| Neopeptide | HLA alleles | Frequency | Neopeptide | HLA alleles | Frequency |
| TLDEAYVMA | HLA-A*02 | 23.52% | IPVAIKELREATLD | DRB1_01 | 6.04% |
| TLDEAYVMASV | HLA-A*02 | 23.45% | IPVAIKELREATLD | DRB1_08 | 4.92% |
| TLDEAYVMASV | HLA-A*68 | 0.07% | IPVAIKELREATLD | DRB1_11 | 2.57% |
| ATLDEAYVM | HLA-A*02 | 5.20% | IPVAIKELREATLD | DRB1_12 | 1.90% |
| ATLDEAYVM | HLA-B*15 | 1.27% | IPVAIKELREATLD | DRB1_14 | 5.38% |
| ATLDEAYVM | HLA-B*35 | 0.33% | IPVAIKELREATLD | DRB1_15 | 0.00% |
| ATLDEAYVM | HLA-B*46 | 0.00% | PVAIKELREATLD | DRB1_01 | 6.04% |
| ATLDEAYVM | HLA-B*58 | 0.00% | PVAIKELREATLDE | DRB1_01 | 6.04% |
| ATLDEAYVM | HLA-C*02 | 0.00% | PVAIKELREATLD | DRB1_08 | 0.00% |
| ATLDEAYVM | HLA-C*03 | 25.32% | PVAIKELREATLDE | DRB1_08 | 0.00% |
| ATLDEAYVM | HLA-C*05 | 0.00% | PVAIKELREATLD | DRB1_11 | 2.57% |
| ATLDEAYVM | HLA-C*08 | 0.00% | PVAIKELREATLDE | DRB1_11 | 2.57% |
| ATLDEAYVM | HLA-C*12 | 2.18% | PVAIKELREATLD | DRB1_12 | 1.90% |
| ATLDEAYVM | HLA-C*14 | 0.00% | PVAIKELREATLDE | DRB1_12 | 1.90% |
| ATLDEAYVM | HLA-C*15 | 3.44% | PVAIKELREATLD | DRB1_14 | 5.38% |
| ATLDEAYVM | HLA-C*16 | 0.32% | PVAIKELREATLDE | DRB1_14 | 5.38% |
| REATLDEAY | HLA-B*13 | 0.00% | VAIKELREATLD | DRB1_01 | 6.04% |
| REATLDEAY | HLA-B*15 | 1.45% | VAIKELREATLD | DRB1_08 | 0.00% |
| REATLDEAY | HLA-B*18 | 0.00% | VAIKELREATLD | DRB1_11 | 2.57% |
| REATLDEAY | HLA-B*35 | 0.44% | VAIKELREATLD | DRB1_12 | 1.90% |
| REATLDEAY | HLA-B*40 | 1.84% | VAIKELREATLD | DRB1_14 | 5.38% |
| REATLDEAY | HLA-B*44 | 4.25% | VAIKELREATLDEA | DRB1_01 | 6.04% |
| REATLDEAY | HLA-B*45 | 0.00% | VAIKELREATLDEA | DRB1_08 | 0.00% |
| REATLDEAY | HLA-B*47 | 0.00% | VAIKELREATLDEA | DRB1_11 | 2.57% |
| REATLDEAY | HLA-B*48 | 0.11% | VAIKELREATLDEA | DRB1_12 | 1.90% |
| REATLDEAYVM | HLA-B*13 | 0.00% | VAIKELREATLDEA | DRB1_14 | 5.38% |
| REATLDEAYVM | HLA-B*15 | 0.50% | VAIKELREATLDE | DRB1_01 | 6.04% |
| REATLDEAYVM | HLA-B*18 | 0.00% | VAIKELREATLDE | DRB1_08 | 0.00% |
| REATLDEAYVM | HLA-B*35 | 0.00% | VAIKELREATLDE | DRB1_11 | 2.57% |
| REATLDEAYVM | HLA-B*39 | 0.00% | VAIKELREATLDE | DRB1_12 | 1.90% |
| REATLDEAYVM | HLA-B*40 | 13.92% | VAIKELREATLDE | DRB1_14 | 5.38% |
| REATLDEAYVM | HLA-B*41 | 0.09% | TLDEAYVMASVDNP | DRB1_01 | 6.04% |
| REATLDEAYVM | HLA-B*44 | 0.00% | TLDEAYVMASVDNP | DRB1_04 | 4.08% |
| REATLDEAYVM | HLA-B*48 | 0.11% | TLDEAYVMASVDNP | DRB1_07 | 0.00% |
| REATLDEAYV | HLA-B*13 | 0.00% | TLDEAYVMASVDNP | DRB1_08 | 0.00% |
| REATLDEAYV | HLA-B*15 | 0.00% | TLDEAYVMASVDNP | DRB1_09 | 0.00% |
| REATLDEAYV | HLA-B*35 | 0.00% | TLDEAYVMASVDNP | DRB1_12 | 0.00% |
| REATLDEAYV | HLA-B*40 | 3.78% | AIKELREATLDEAY | DRB1_01 | 2.02% |
| REATLDEAYV | HLA-B*41 | 0.18% | AIKELREATLDEAY | DRB1_08 | 0.00% |
| REATLDEAYV | HLA-B*44 | 0.00% | AIKELREATLDEAY | DRB1_11 | 0.00% |
| REATLDEAYV | HLA-B*45 | 0.00% | AIKELREATLDEAY | DRB1_12 | 1.90% |
| LREATLDEAY | HLA-B*15 | 0.53% | AIKELREATLDEAY | DRB1_14 | 5.38% |
| LREATLDEAY | HLA-B*18 | 0.00% | AIKELREATLDEA | DRB1_01 | 2.02% |
| LREATLDEAY | HLA-B*35 | 0.33% | AIKELREATLDEA | DRB1_08 | 0.00% |
| LREATLDEAY | HLA-B*40 | 0.95% | AIKELREATLDEA | DRB1_11 | 0.00% |
| LREATLDEAY | HLA-B*44 | 0.07% | AIKELREATLDEA | DRB1_12 | 1.90% |
| LREATLDEAY | HLA-B*48 | 0.11% | AIKELREATLDEA | DRB1_14 | 5.38% |
| KELREATL | HLA-B*40 | 2.60% | REATLDEAYVMASV | DRB1_01 | 6.04% |
| KELREATL | HLA-B*41 | 0.09% | REATLDEAYVMASV | DRB1_04 | 0.00% |
| ATLDEAYVMA | HLA-A*02 | 0.21% | REATLDEAYVMASV | DRB1_08 | 0.00% |
| ELREATLDEAY | HLA-A*26 | 0.05% | REATLDEAYVMASV | DRB1_12 | 0.00% |
| ELREATLDEAY | HLA-B*15 | 6.05% | EATLDEAYVMASV | DRB1_01 | 6.04% |
| ELREATLDEAY | HLA-B*35 | 0.00% | EATLDEAYVMASV | DRB1_08 | 0.00% |
| ELREATLDEAY | HLA-B*40 | 0.07% | EATLDEAYVMASV | DRB1_12 | 0.00% |
| EATLDEAYV | HLA-A*68 | 0.07% | ATLDEAYVMASVDN | DRB1_01 | 6.04% |
| EATLDEAYV | HLA-A*69 | 0.26% | ATLDEAYVMASVDN | DRB1_04 | 0.00% |
| EATLDEAYVM | HLA-A*26 | 0.05% | ATLDEAYVMASVDN | DRB1_08 | 0.00% |
| EATLDEAYVM | HLA-B*35 | 0.00% | ATLDEAYVMASVDN | DRB1_09 | 0.00% |
| AIKELREATL | HLA-B*08 | 0.00% | ATLDEAYVMASVDN | DRB1_12 | 0.00% |
| ATLDEAYV | HLA-A*02 | 0.00% | EATLDEAYVMASVD | DRB1_01 | 6.04% |
|  |  |  | EATLDEAYVMASVD | DRB1_08 | 0.00% |
|  |  |  | EATLDEAYVMASVD | DRB1_12 | 0.00% |
|  |  |  | AIKELREATLD | DRB1_01 | 2.02% |
|  |  |  | AIKELREATLD | DRB1_08 | 0.00% |
|  |  |  | AIKELREATLD | DRB1_11 | 0.00% |
|  |  |  | AIKELREATLD | DRB1_12 | 0.00% |
|  |  |  | AIKELREATLD | DRB1_14 | 1.67% |
|  |  |  | ATLDEAYVMASV | DRB1_01 | 6.04% |
|  |  |  | ATLDEAYVMASV | DRB1_08 | 0.00% |
|  |  |  | ATLDEAYVMASV | DRB1_12 | 0.00% |
|  |  |  | AIKELREATLDE | DRB1_01 | 2.02% |
|  |  |  | AIKELREATLDE | DRB1_08 | 0.00% |
|  |  |  | AIKELREATLDE | DRB1_11 | 0.00% |
|  |  |  | AIKELREATLDE | DRB1_12 | 0.00% |
|  |  |  | AIKELREATLDE | DRB1_14 | 1.67% |
|  |  |  | LREATLDEAYVMAS | DRB1_01 | 2.02% |
|  |  |  | LREATLDEAYVMAS | DRB1_12 | 0.00% |
|  |  |  | ATLDEAYVMASVD | DRB1_01 | 2.02% |
|  |  |  | REATLDEAYVMAS | DRB1_01 | 0.00% |
|  |  |  | ATLDEAYVMASVD | DRB1_08 | 0.00% |
|  |  |  | REATLDEAYVMAS | DRB1_12 | 0.00% |
|  |  |  | ATLDEAYVMASVD | DRB1_12 | 0.00% |
|  |  |  | TLDEAYVMASVDN | DRB1_01 | 2.02% |
|  |  |  | ELREATLDEAYVMA | DRB1_01 | 0.00% |
|  |  |  | IKELREATLDEAYV | DRB1_01 | 0.00% |
|  |  |  | IKELREATLDEAYV | DRB1_08 | 0.00% |
|  |  |  | TLDEAYVMASVDN | DRB1_08 | 0.00% |
|  |  |  | IKELREATLDEAYV | DRB1_12 | 0.00% |
|  |  |  | ELREATLDEAYVMA | DRB1_12 | 0.00% |
|  |  |  | IKELREATLDEAY | DRB1_01 | 0.00% |
|  |  |  | LREATLDEAYVMA | DRB1_01 | 0.00% |
|  |  |  | IKELREATLDEAY | DRB1_08 | 0.00% |
|  |  |  | IKELREATLDEAY | DRB1_12 | 0.00% |
|  |  |  | LREATLDEAYVMA | DRB1_12 | 0.00% |
|  |  |  | EATLDEAYVMAS | DRB1_01 | 0.00% |
|  |  |  | IKELREATLDEA | DRB1_08 | 0.00% |
|  |  |  | IKELREATLDEA | DRB1_12 | 0.00% |
|  |  |  | REATLDEAYVMA | DRB1_01 | 0.00% |
|  |  |  | ATLDEAYVMAS | DRB1_01 | 0.00% |
|  |  |  | TLDEAYVMASV | DRB1_01 | 0.00% |
|  |  |  | TLDEAYVMASVD | DRB1_01 | 0.00% |
|  |  |  | IKELREATLD | DRB1_08 | 0.00% |
|  |  |  | IKELREATLDE | DRB1_08 | 0.00% |
| Total |  | 82.07% |  |  | 24.89% |
