# Supplementary material for: Immunogenicity of Del19 EGFR mutations in Chinese patients affected by lung adenocarcinoma
Source: BMC Immunol. 2019 Nov 13;20:43. doi: 10.1186/s12865-019-0320-1 (PMC6854806; doi:10.1186/s12865-019-0320-1)
Supplement: Supplementary file 12 — Additional file 12. Comparison between EGFR exon Del 19 and EGFR L858R derived peptides. [file 12865_2019_320_MOESM12_ESM.doc]

**Supplemental Table 12, Comparison between EGFR exon Del 19 and EGFR L858R derived peptides**

|  | No. of neoepitope for MHC I | No. of neoepitope for MHC II |
| --- | --- | --- |
| EGFR 19DEL | 155 | 1363 |
| EGFR L858R | 17 | 78 |
